# Image-seq: spatially resolved single-cell sequencing guided by in situ and in vivo imaging

**DOI:** 10.1038/s41592-022-01673-2

**Published:** 2022-11-24

**Authors:** Christa Haase, Karin Gustafsson, Shenglin Mei, Shu-Chi Yeh, Dmitry Richter, Jelena Milosevic, Raphaël Turcotte, Peter V. Kharchenko, David B. Sykes, David T. Scadden, Charles P. Lin

**Affiliations:** 1 Wellman Center for Photomedicine, Massachusetts General Hospital, Boston, MA USA; 2 Center for Systems Biology, Massachusetts General Hospital, Boston, MA USA; 3 Center for Regenerative Medicine, Massachusetts General Hospital, Boston, MA USA; 4 Harvard Stem Cell Institute, Cambridge, MA USA; 5 Department of Stem Cell and Regenerative Biology, Harvard University, Cambridge, MA USA; 6 Department of Biomedical Informatics, Harvard Medical School, Boston, MA USA; 7 Present Address: Department of Orthopaedics, Center for Musculoskeletal Research, University of Rochester Medical Center, Rochester, NY USA; 8 Present Address: Altos Labs, San Diego, CA USA

**Keywords:** Multiphoton microscopy, Cancer stem cells, RNA sequencing, Optical imaging, Cancer imaging

## Abstract

Tissue function depends on cellular organization. While the properties of individual cells are increasingly being deciphered using powerful single-cell sequencing technologies, understanding their spatial organization and temporal evolution remains a major challenge. Here, we present Image-seq, a technology that provides single-cell transcriptional data on cells that are isolated from specific spatial locations under image guidance, thus preserving the spatial information of the target cells. It is compatible with in situ and in vivo imaging and can document the temporal and dynamic history of the cells being analyzed. Cell samples are isolated from intact tissue and processed with state-of-the-art library preparation protocols. The technique therefore combines spatial information with highly sensitive RNA sequencing readouts from individual, intact cells. We have used both high-throughput, droplet-based sequencing as well as SMARTseq-v4 library preparation to demonstrate its application to bone marrow and leukemia biology. We discovered that DPP4 is a highly upregulated gene during early progression of acute myeloid leukemia and that it marks a more proliferative subpopulation that is confined to specific bone marrow microenvironments. Furthermore, the ability of Image-seq to isolate viable, intact cells should make it compatible with a range of downstream single-cell analysis tools including multi-omics protocols.

## Abstract (web summary)

Image-seq isolates cells from specific tissue locations under image guidance for analysis by single-cell RNA sequencing. The technique can be combined with in vivo imaging to document the temporal and dynamic history of the cells prior to sequencing.

## Main

Spatial transcriptomics is a rapidly advancing field that encompasses a range of different technologies capable of spatially resolved gene expression analysis^1–9^. In contrast to single-cell RNA sequencing (scRNA-seq), which provides deep insights into heterogeneities within cell populations but does not preserve the spatial relationships between individual cells, techniques such as MERFISH^9^, seqFISH^4^ and Slide-seq^8^ can link these heterogeneities to differences in spatial composition and cellular proximity. However, apart from Niche-seq^7^, they have been applied only in vitro or they have relied on the generation of tissue sections^3–5,8,10^, which confines their applicability to tissues that are easily sectioned. They map the gene expression profiles onto two-dimensional (2D) images, and extrapolation to three-dimensional (3D) architecture of intact tissues is still limited. For example, 3D expression profiles and cell segmentation have been demonstrated in tissue sections^11,12^ but the section thickness is limited by messenger RNA probe diffusion. In addition, tissue sections can provide only static images, necessitating the use of indirect methods such as pseudo-time analysis to infer cellular trajectories over time^13,14^, and to our knowledge none of the currently available spatial transcriptomics technologies has been combined with in vivo imaging.

Here, we present Image-seq, a new platform that enables image-guided cell isolation for scRNA-seq. The core of the Image-seq platform is a multiphoton microscope with two optical paths, one for imaging and one for laser micromachining that creates an access channel in tissue through which a micropipette is brought to the target location to aspirate cells under image guidance. Because it captures viable cells it can be combined with state-of-the-art library preparation protocols, leading to higher mRNA detection efficiencies and broader transcript coverage than other spatial sequencing technologies. In addition, standard computational tools can be used for data analysis.

We use Image-seq to study the bone marrow, the primary site of hematopoiesis, where hematopoietic stem cells give rise to all of the blood cells in the body. The bone marrow is also the site where malignant cells can either originate (such as leukemia) or preferentially metastasize to (such as prostate or breast cancer). Although the functional organization of hematopoietic cells in the bone marrow has been extensively characterized^15^, their 3D spatial organization has been difficult to assess due to their complexity and location deep inside the bone matrix^16^. To demonstrate Image-seq’s versatility and high sensitivity, we used high-throughput, droplet-based sequencing with the 10x Chromium platform to profile bone marrow hematopoietic cells, as well as the SMARTseq-v4 protocol to profile rare (<0.01% leukemic burden) acute myeloid leukemia (AML) cells and bone marrow stromal cells. Specifically, we tracked AML progression using intravital microscopy, and observed pronounced spatial heterogeneity in the earliest stage of expansion from single AML cells seeded in the bone marrow. This highlighted the need to capture these cells from distinct bone marrow locations where they either proliferate or remain quiescent, to identify the factors that regulate leukemia progression in the earliest stage of the disease development. Using this approach we identified DPP4 (dipeptidyl peptidase 4) as a key upregulated gene in AML cells from the more proliferative bone marrow compartments. Strikingly, DPP4 was not expressed on the same cells cultured in vitro, suggesting that DPP4 was specifically activated in vivo and was correlated with disease progression.

## Results

### Cell isolation under image guidance

The core of the Image-seq platform is a confocal or multiphoton microscope with an additional laser beam capable of tissue ablation. The requirement for the ablation laser is that it needs to have sufficient pulse energy (>10 nJ per pulse, or approximately 10-fold the pulse energy typically used in multiphoton microscopy) to generate a plasma through multiphoton ionization at the laser focus. To minimize thermal damage it also needs to have a low average power, which can be accomplished by reducing the pulse repetition frequency from the ~80 MHz typically used for multiphoton microscopy to a few MHz to compensate for the higher pulse energy needed for tissue ablation. These requirements are readily fulfilled with commercial or industrial femtosecond fiber lasers for micromachining or ophthalmic microsurgery^17^. The plasma is used to ablate or etch away bone^18,19^ with minimal collateral damage to the surrounding tissue^18^. A small, ~50 × 100 µm channel is created, through which a micropipette, controlled by a micromanipulator, is inserted to aspirate live bone marrow cells under image guidance. The sample is expelled from the micropipette, which directly generates a single-cell suspension.

**Fig. 1:**
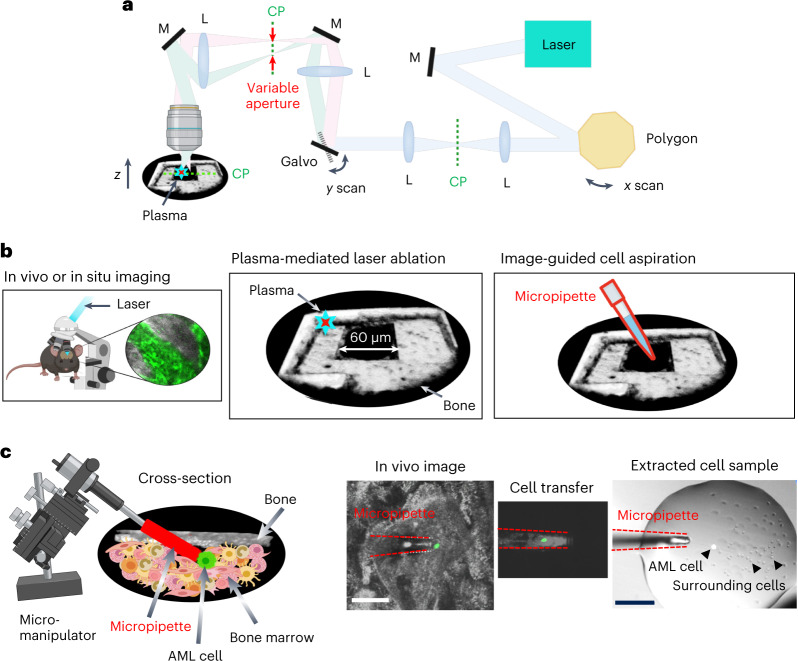
Optical set-up and cell aspiration procedure. **a**, Schematic diagram of integral components of the combined multiphoton microscopy and laser ablation set-up (full diagram in Extended Data Fig. 1). CP, conjugate plane; galvo, scanning galvanometer mirror; L, lens; M, mirror. **b**, Cell aspiration procedure that includes multiphoton microscopy, plasma-mediated laser ablation and insertion of the micropipette into the bone marrow for cell aspiration. **c**, Positioning of the micropipette next to the target cell using a micromanipulator, and cell aspiration with the micropipette (red) all under image guidance. The extracted cell sample (target cell with ~100 surrounding cells) is shown after transfer to a droplet on a glass slide and visualized with a conventional fluorescence microscope. A total of 21 biological replicates of in vivo cell aspiration were performed in 10 independent mice. Scale bars, 50 μm.

**Extended Data Fig. 1:**
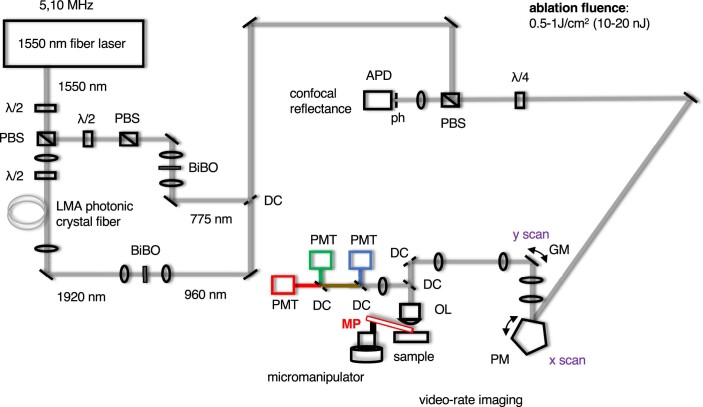
Full optical diagram of Image-seq set-up. Radiation at 775 nm is generated by frequency doubling the 1550 nm output from a fiber laser. Radiation at 920-980 nm is generated by soliton self-frequency shift of 1550 nm radiation to 1840–1960nm in a LMA photonic crystal fiber, which is subsequently frequency doubled to obtain the imaging wavelength. A scanning GM (y scan) and scanning PM (x scan) are conjugated to one another, along with the back aperture of the OL so that a scan along the x and y dimensions is achieved in the image plane. Multiphoton emission wavelengths are collected with a combination of DCs and PMTs to generate an image. A confocal reflectance channel that utilizes an APD and 20µm-size pinhole can also be used to generate images. 𝛌/2: 𝛌/2-plate. PBS: polarization beam splitter. BiBO: BiBO3 - Bismuth orthoborate crystal. LMA: large mode-area. APD: avalanche photodiode. ph: pinhole. DC: dichroic mirror. GM: galvo mirror. PM: polygonal mirror. PMT: photomultiplier tube. MP: micropipette. OL: objective lens.

In our implementation the imaging arm and the ablation arm are powered by a single femtosecond fiber laser operating at 5 MHz repetition frequency. This repetition frequency enables full-field (500 × 500 pixels) image acquisition at 15 frames per second (half the video rate) with a single laser pulse per pixel. However, it is also possible to integrate an ablation capability into an existing confocal or multiphoton microscope by adding an ablation laser that fulfills the requirements described above. A detailed optical design is shown in Extended Data Fig. 1. The output of a 1,550 nm femtosecond fiber laser is frequency doubled to 775 nm and used for both imaging and plasma-mediated laser ablation by adjusting the pulse energy between 1 and 20 nJ. An additional imaging wavelength that is tunable between 940 and 980 nm is provided through soliton generation in a photonic crystal fiber^20^. A revolving polygon and scanning galvanometer mirror, conjugated to one another and the back aperture of the objective lens, steer the ablation and imaging beams across the field of view in the imaging plane (Fig. 1a). A variable aperture at the intermediate image plane controls the 2D ablation geometry along the *x* and *y* dimensions (Fig. 1b) while the sample is translated along the *z* dimension to either ablate or image a 3D tissue volume. By etching through bone a small opening (~50 µm × 100 µm) is created, and a micromanipulator is used to insert a fluorescently coated micropipette through the opening at a 20° angle into the bone marrow with micrometer spatial precision (Fig. 1b). Prior to making the opening, a larger area of bone is thinned (~200 × 300 µm) to improve micropipette accessibility and imaging depth. This step takes ~3 min. Target bone marrow cells are aspirated into the micropipette by suction with an Air Syringe, with the displaced volume controlling the total number of aspirated cells (from a few to a few thousand). With this design we were able to aspirate bone marrow cells from freshly collected bone samples as well as from live animals while tracking the process in real time (Supplementary Videos 1–3). Figure 1c shows the bone marrow aspirate containing the target cell from the in vivo image after transfer to a glass slide and visualization with a wide-field fluorescence microscope. Using this strategy, recovery of a single, viable target cell has an efficiency of ~70%.

#### Similarity of whole bone marrow and micropipette sample composition

**Fig. 2:**
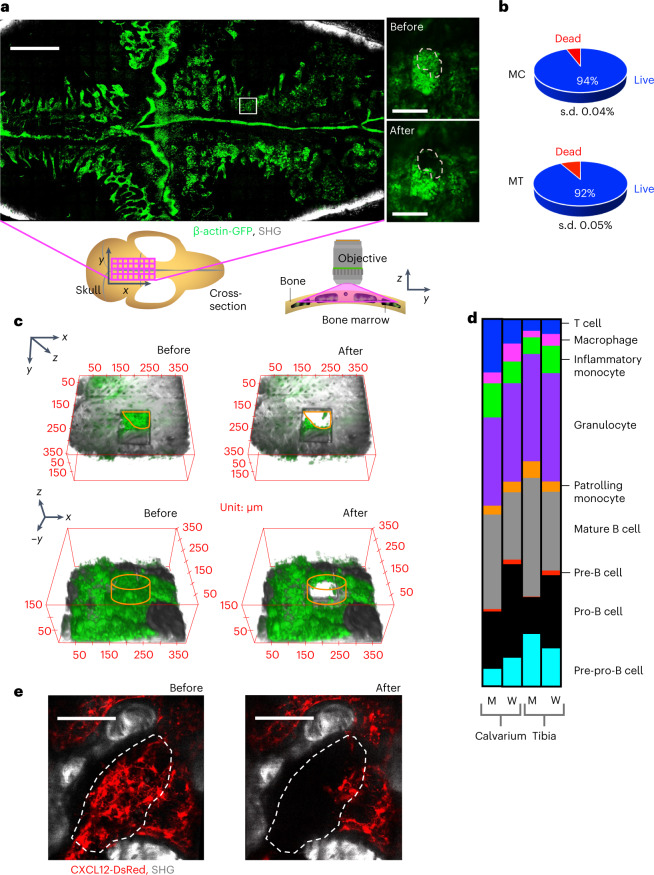
Characterization of micropipette samples. **a**, Tiled, maximum intensity projection image of a 4 × 8 mm region of skull bone marrow (tiles shown as magenta squares on the schematic of the skull below the image), obtained by stitching together individual microscope fields of view. Green, β-actin-GFP-positive bone marrow cells; gray, second-harmonic generation (SHG) bone signal. White square: region of interest that is shown in the insets before and after cell isolation. Scale bars: main image, 1 mm; insets, 100 μm. Tiled calvarium bone marrow images were obtained from three independent mice. **b**, Average cell viability for the calvarium (MC), from a total of nine micropipette samples in *n* = 3 mice. Cell viability for the tibia (MT), from four micropipette samples in *n* = 2 mice. **c**, 3D visualization of a target location before and after cell aspiration. The bottom panel is obtained by rotating the top panel by 180° around the *x* axis. Isolated cell volume is marked by an orange outline, and the extracted cell volume (diameter ~80 µm, height 50 µm) contained in a typical micropipette sample is outlined by an orange cylinder. **d**, Averaged cell proportions from flow cytometry analysis of 15 calvarial micropipette (M) and 8 whole calvarial bone marrow (W) preparations in a total of *n* = 10 mice, and 14 tibia micropipette (M) and 5 whole tibia bone marrow (W) preparations in a total of *n* = 7 mice. Cells were classified based on their cell surface properties as T cells: Ter119^−^CD71^−^, CD11b^−^, CD3^+^; mature B cells: Ter119^−^CD71^−^, CD3^−^, CD11b^−^, B220^+^, CD19^+^, cKit^−^ IgM^+^; pro-B cells: Ter119^−^CD71^−^, CD3^−^, CD11b^−^, B220^+^, CD19^+^, IgM^−^, cKit^+^; pre-B cells: Ter119^−^CD71^−^, CD3^−^, CD11b^−^, B220^+^, CD19^+^, IgM^−^, cKit^−^; Pre-Pro-B cells: Ter119^−^CD71^−^, CD3^−^, CD11b^−^, CD19^−^, B220^+^; macrophages: Ter119^−^CD71^−^, CD3^−^, CD11b^+^, Ly6C^−^, F4/80^+^; inflammatory monocytes: Ter119^−^CD71^−^, CD3^−^, CD11b^+^, F4/80^−^, Ly6CHigh; patrolling monocytes: Ter119^−^CD71^−^, CD3^−^, CD11b^+^, F4/80^−^, Gr1^−^, Ly6CLow; granulocytes: Ter119^−^CD71^−^, CD3^−^, CD11b^+^, F4/80^−^, Ly6CLow, Gr1^+^. See Extended Data Fig. 2 for the gating strategy. **e**, Maximum intensity projection image of a bone marrow region before and after stromal cell aspiration with the micropipette. Red, CXCL12-DsRed bone marrow cells; gray, SHG bone signal. Scale bar, 100 µm. Fifty biological replicates of stromal cell extraction were performed in 14 independent mice. Source data

**Extended Data Fig. 2:**
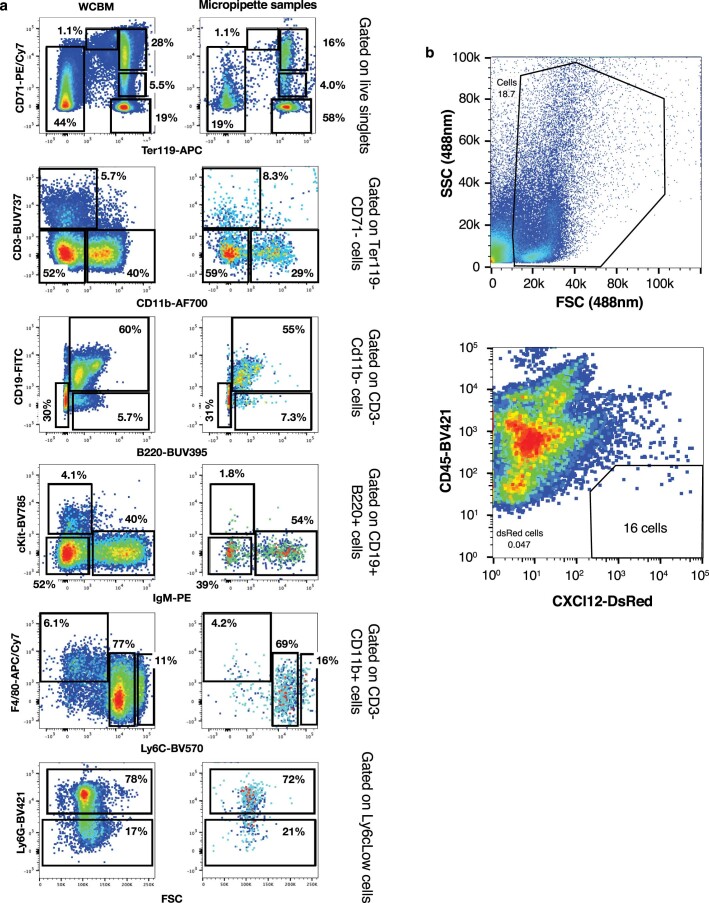
Gating strategy for flow cytometry analysis of samples from Fig. 2. a) Gating strategy for the analysis of various leukocyte populations in a whole calvarium bone marrow preparation (WCBM) and concatenated data from all micropipette samples. Analyzed cells were gated on singlet live cells and percentages refer to percent of parent gate. The same gating strategy was used for the analysis of whole tibia and tibia micropipette samples. b) Gating strategy for flow cytometry analysis of CXCl12^+^, CD45^−^ stromal cells from 5 concatenated micropipette samples.

**Extended Data Fig. 3:**
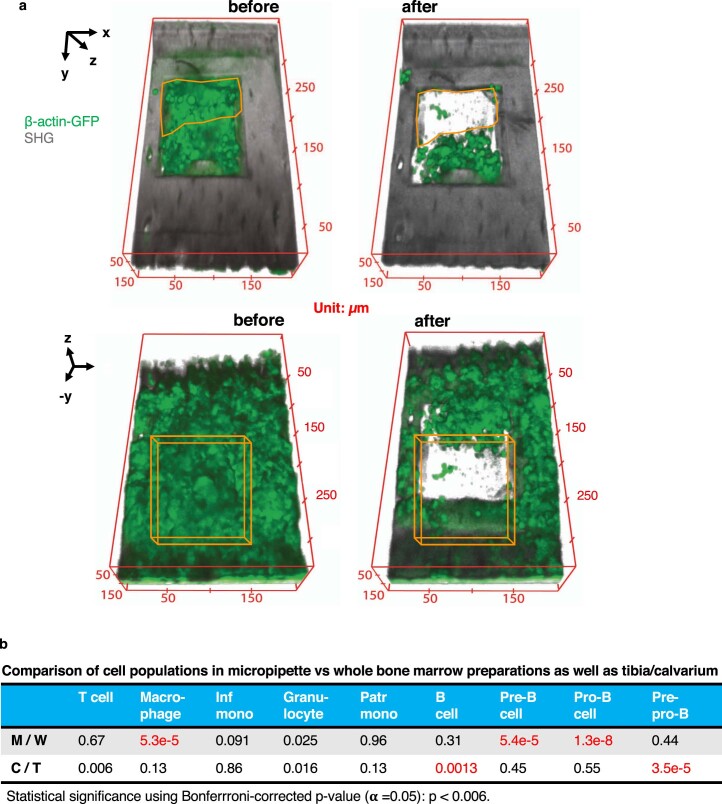
Long bone cell extraction. a) 3D visualization of a target location in a β-actin-GFP mouse before and after cell aspiration. The bottom panel is obtained by rotating the top panel by 180° around the x axis. The isolated cell volume is marked by an orange outline. b) Comparison of micropipette and whole bone marrow preparations from the calvarium and tibia. Statistical analysis was performed using the two-way ANOVA for each individual cell population, with the two independent variables being bone type (tibia vs calvarium) and procedure (micropipette vs whole bone marrow preparations). Listed p values were not adjusted for multiple comparisons. Using a Bonferroni correction, statistical significance is achieved for p < 0.006 (𝛂=0.05); significant p values are highlighted in red. Source data

To further demonstrate our instrument’s ability to isolate live cells from defined anatomic regions of murine bone marrow, we used it to isolate bone marrow samples from β-actin-green fluorescent protein (β-actin-GFP) mice. Figure 2a shows a 4 × 8 mm, tiled, maximum intensity projection image of the calvarium bone marrow, along with a region of interest from which cells were aspirated both before and after cell extraction. To minimize sample loss we eliminated post-extraction sample processing by performing transcardial perfusion prior to cell isolation. This reduced the amount of red blood cells in our samples, and we directly generated a single-cell suspension by expelling samples from the micropipette. There is a notable variation in the CD71^−^Ter119^+^ cell compartment that we attribute to slight differences in perfusion quality (Extended Data Fig. 2a). A total of nine calvarial bone marrow micropipette samples and four tibia bone marrow micropipette samples, containing an average of 2,000 cells each, were isolated from a total of five mice, yielding an average viability of 94% and 92%, respectively (Fig. 2b). A 3D visualization of a representative volume of calvarial bone marrow before and after cell extraction is shown in Fig. 2c. Similarly, Extended Data Fig. 3a shows a representative volume of tibia bone marrow both before and after cell extraction. Both the view from above the bone surface and that achieved by rotation of 180° around the *x* axis are shown. We additionally analyzed extracted cell samples for their cell surface marker expression by flow cytometry and were able to detect the major hematopoietic cell populations in our pooled micropipette samples with similar frequency to those in whole bone marrow control samples (Fig. 2d and Extended Data Figs. 2a,3b). Generally, the cell proportions were in good agreement, although we did observe statistically significant differences between tibia and calvarium bone marrow for the B-cell and pre-pro-B-cell populations. We also observed significant differences between the micropipette and whole bone marrow preparations for macrophage, pre-B-cell and pro-B-cell populations. It is unclear whether the differences between micropipette and whole bone marrow preparations are due to technical effects or the fact that we typically collected micropipette samples from within ~80 µm of the endosteal surface.

By perfusing with enzymatic digestion buffer after the initial perfusion (to reduce the number of red blood cells) and incubating the bone sample for ~20 min (see Methods for details), we were able to establish a protocol for the isolation of stromal cells. Figure 2e shows a maximum intensity projection image of a volume of CXCL12 (C-X-C motif chemokine ligand 12)-DsRed-expressing bone marrow cells both before and after cell extraction. Extended Data Fig. 2b shows the gating strategy used for quantification of CD45^−^CXCL12^+^ stromal cells in our micropipette samples: we detected 3–4 cells in a typical aspirate.

#### Validation of Image-seq technology

**Fig. 3:**
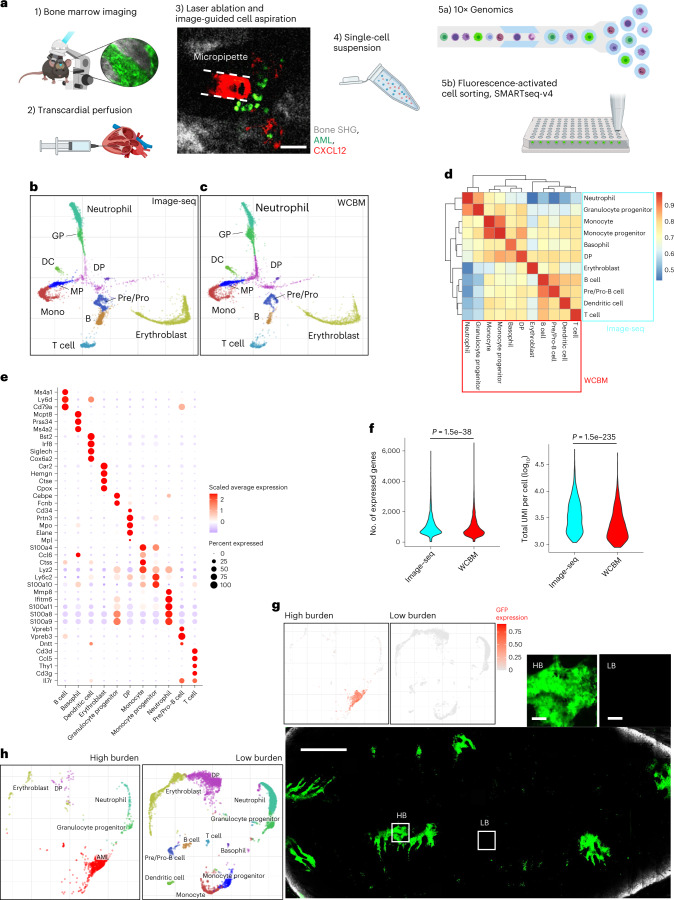
Image-seq analysis of calvarium bone marrow using the 10x Genomics Chromium platform. **a**, scRNA-seq workflow. For the experiments in this fig., step 5a was chosen as the final step. Scale bar, 50 µm. **b**,**c**, UMAP embedding of joint alignment of all Image-seq data (**b**) and WCBM data (**c**), color coded by the major cell populations. B, B cell; DC, dendritic cell; DP, diverse progenitors; GP, granulocyte progenitor; MP, monocyte progenitor; Pre/Pro, Pre- and Pro-B cell; Mono, monocyte. **d**, Heatmap showing Spearman correlation coefficients of gene average expression level between WCBM and Image-seq for each cell type. **e**, Dot plots showing selected marker gene expression across major cell populations for the combined Image-seq and WCBM data. The color represents scaled average expression of marker genes in each cell type, and the dot size indicates the proportion of cells expressing the individual marker gene. **f**, Violin plots comparing the number of expressed genes and total unique molecular identifiers (UMIs) per cell between WCBM and Image-seq samples. Statistical significance was determined using a two-sided Wilcoxon rank sum test. **g**, Tiled, maximum intensity projection image of leukemic bone marrow at day 10 after transplantation; examples of high-burden (HB) and low-burden (LB) regions marked by white squares and shown in the inset. Green, AML-GFP; gray, bone SHG. Scale bars: main image, 1 mm; insets, 50 μm. Also included is a distribution of GFP expression in UMAP embeddings of scRNA-seq data obtained from HB and LB regions. Tilescans were obtained from *n* = 5 independent mice. A total of *n* = 4 biological replicates of cell extraction from HB and *n* = 4 biological replicates of cell extraction from LB regions were performed in six independent mice. A total of four HB and LB cell extractions were performed in the same mice from which we also obtained tilescans. Sequencing was performed on one LB and one HB sample. **h**, UMAP embeddings of HB and LB samples color coded by cell type, and using the annotations given in **b**. Source data

An outline of the Image-seq workflow is shown in Fig. 3a. Regions of interest are identified by multiphoton or confocal imaging, mice are perfused, and laser ablation, combined with cell aspiration by micropipette, is then used to isolate cell samples. The entire procedure takes ~20 min per location.

A single-cell suspension is generated by expelling the sample from the micropipette and into a tube that is transferred directly to the 10x Chromium chip for high-throughput characterization of the entire cell sample. Alternatively, samples can be stained and individual cell populations sorted into wells by flow cytometry for subsequent library preparation and sequencing using the SMARTseq-v4 protocol. To validate the technology we collected 11 Image-seq samples from a total of *n* = 5 C57Bl6 mice, along with three whole calvarium bone marrow (WCBM) samples (*n* = 3 mice) for single-cell isolation and library preparation using the 10x Chromium platform (see Methods for details). After sequencing and read alignment we used the Conos package in R (ref. ^21^) to integrate our multiple scRNA-seq datasets and align them with other public scRNA-seq data^22^. We used Leiden clustering to determine joint cell clusters across the entire dataset, identifying most of the major hematopoietic cell populations, and visualizing them by UMAP (uniform manifold approximation and projection) embedding (Fig. 3b,c). The population at the plot center expresses multi-lineage progenitor markers such as *Cd34*, *Cebpa*, *Hoxa9* and *Meis1* (refs. ^23,24^), as well as genes associated with granulocytic and monocytic priming (*Mpo*, *Elane*, *Ctsg*, *Prtn3* (refs. ^23–25^) and with megakaryocytic priming (*Mpl*, *Mycn*^24,25^), and was therefore termed 'diverse progenitors'. From here, lymphoid (marked by expression of *Vpreb1-3*, *Rag1-2*, *Cd19*, *Dntt*, *Mzb1*, *Ebf1* (refs. ^3,22,26^)), erythroid (marked by expression of *Car1-2*, *Hemgn*, *Ctse*, *Cpox,* *Smim1* (refs. ^22,24,26^)), granulocytic (*Cebpe*, *Fcnb*, *S100a8*, *S100a9*, *Camp*^23–25^) and monocytic (*Irf8*, *Klf1*, *F13a1*, *S100a4*, *S100a10*, *Ly6c2* (refs. ^22–25^)) lineages branch off in different directions. Dot plots of selected marker genes for each cell population are shown in Fig. 3e. A list of differentially expressed genes in each cluster is given in Supplementary Table 1. Here, we pooled 11 Image-seq samples to characterize the distribution of cell populations in the limit of a large number of samples (for which we would expect the same distribution as for WCBM samples if there were no technical differences between the two sampling strategies).

**Extended Data Fig. 4:**
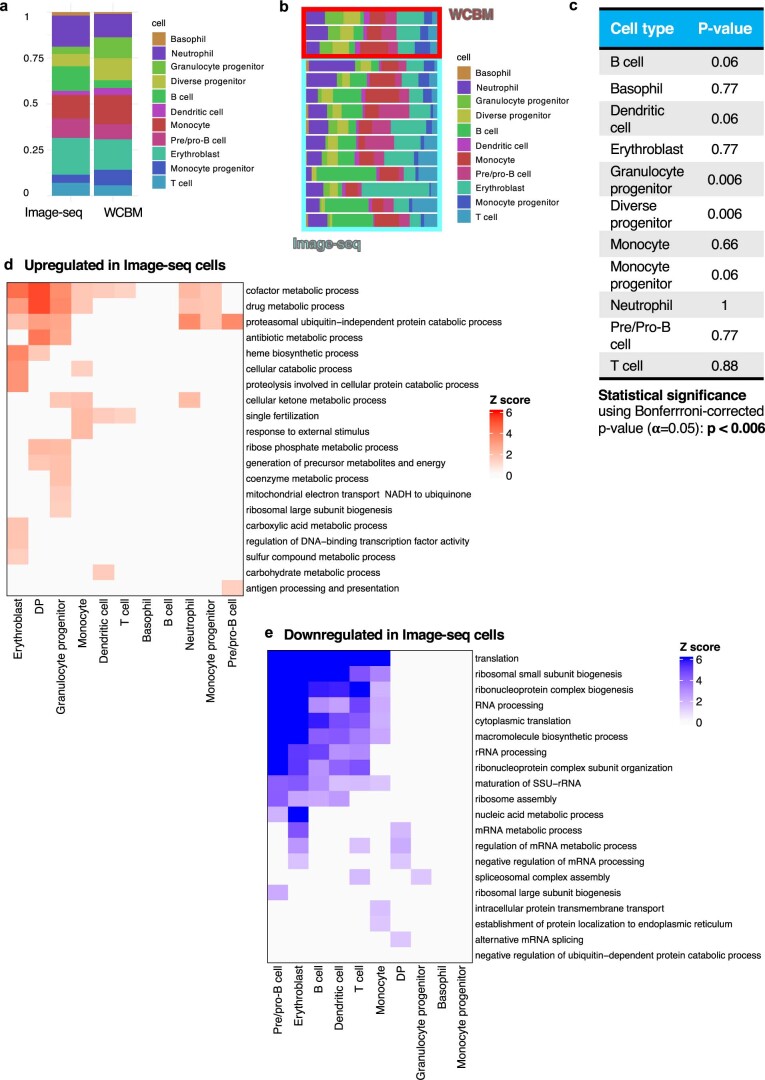
Distribution of cell proportions in Image-seq samples along with GO analysis of differentially expressed genes. a) Distribution of cell proportions for the major hematopoietic cell populations from Fig. 3b,c, shown as averaged proportions over all Image-seq vs all WCBM samples. b) Breakdown of (a) showing the distribution of cell proportions for each sample. WCBM samples outlined by red border, Image-seq samples outlined by blue border. c) Table with p values for differences in cell proportions between WCBM and Image-seq samples (obtained using a two-sided Wilcoxon rank sum test). d) Top GO terms obtained from upregulated genes in Image-seq samples, broken down by cell type. e) Top GO terms obtained from downregulated genes in Image-seq samples, broken down by cell type. WCBM: whole calvarial bone marrow. GO: gene ontology. Pre/Pro-B cell: Pre and pro B cell. DP: Diverser progenitor. Source data

**Extended Data Fig. 5:**
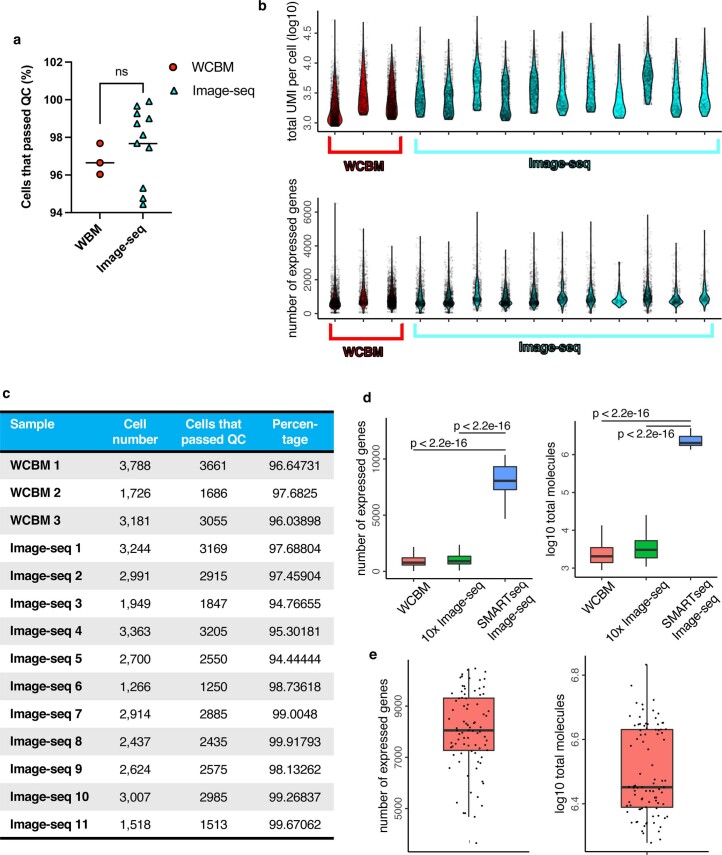
Quality control metrics for the single-cell RNA-seq data from Figs. 3, 4 and Extended Data Fig. 6. a) Percentage of cells that passed quality control after sequencing in each 10x run (WCBM N = 3, Image-seq N = 11 biological replicates). A two-sided Mann–Whitney test was used to assess statistical significance. 3 (WCBM) and 5 (Image-seq) independent mice were used, respectively. b) Number of unique molecular identifiers and expressed genes per cell for each Image-seq and whole calvarium bone marrow (WCBM) sample. c) Table that summarizes the number of cells sequenced from each 10x run, along with the number of cells and percentage of cells that passed quality control. d) Comparison of the number of expressed genes and total number of molecules (= total UMI) between our WCBM, Image-seq and AML SMARTseq-v4 data. Statistical significance was determined using a two-sided Wilcoxon rank sum test. e) Number of expressed genes per cell and total mapped reads (= molecules) per cell for the AML SMARTseq-v4 data, including scatter plot. d),e) WCBM N = 5295 (3 independent mice), Image-seq N = 8078 (5 independent mice), SMARTseq N = 84 cells (11 independent mice). Boxplot represents the interquartile range (IQR, the range between the 25th and 75th percentile), the mid-point of the data is indicated as a line within. Whiskers indicate the upper and lower value that is within 1.5 times the IQR. Source data

As expected for sampling small volumes from different locations, we observed significant sample-to-sample variations (a detailed breakdown is given in Extended Data Fig. 4b). However, we did not observe statistically significant differences in the proportion of any cell populations when comparing Image-seq (Fig. 3b) and WCBM (Fig. 3c) preparations in aggregated form (Extended Data Fig. 4c). We further examined the transcriptional similarity of the major cell types from WCBM and Image-seq samples. Correlation coefficients of average gene expression levels between WCBM and Image-seq samples (Fig. 3d) show a strong correlation between individual cell populations collected using the two methodologies. Gene ontology analysis of differentially expressed genes between Image-seq and WCBM samples for the most part showed very general terms that were not associated with cell damage or cell stress (Extended Data Fig. 4d,e), although there was a notable downregulation of ribosomal terms in Image-seq samples, which could indicate lower cell stress and could be related to the overall higher number of unique molecular identifiers and genes per cell that we observed in the Image-seq samples (Fig. 3f). We additionally assessed the percentage of cells that passed quality control using the two experimental strategies (Extended Data Fig. 5a) and found no statistically significant differences. We plotted the distribution of unique molecular identifiers and genes per cell for each sample that we collected (Extended Data Fig. 5b), along with a table that summarizes the overall number of cells in each sample and the ones that passed quality control (Extended Data Fig. 5c). Again, although some variations were observed between samples, the data as a whole led us to conclude that Image-seq produces scRNA-seq data with a quality that is comparable to or exceeds that of conventional cell collection protocols.

**Extended Data Fig. 6:**
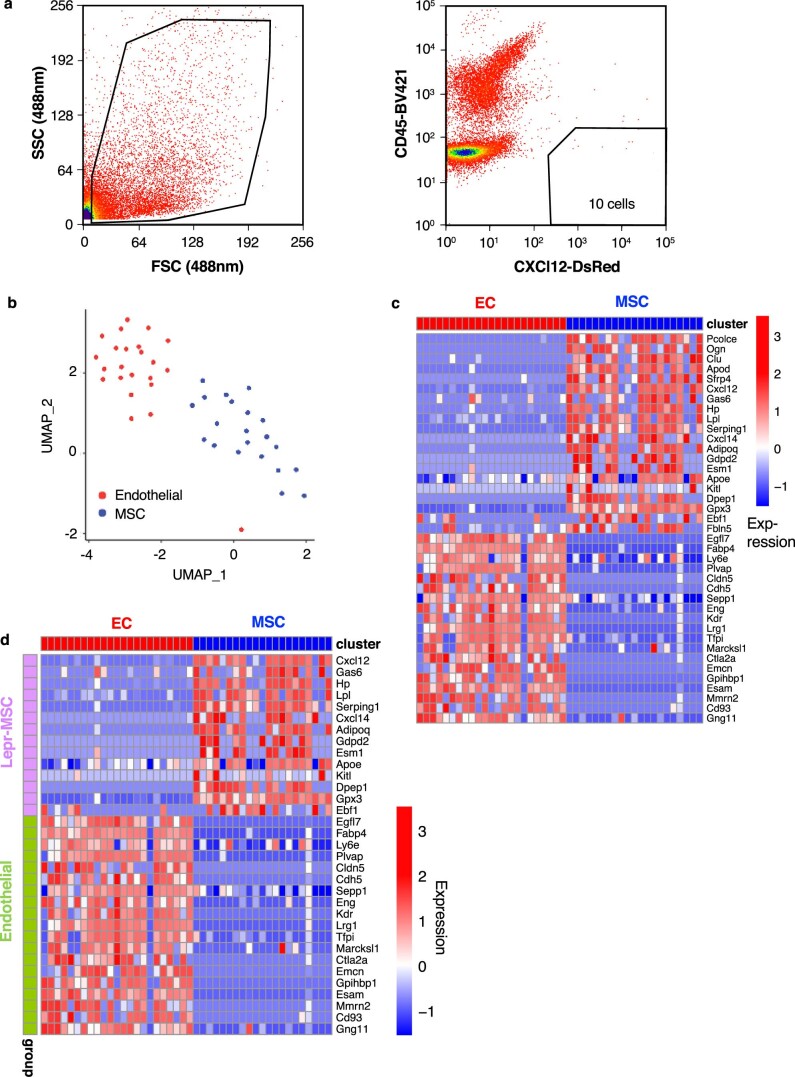
Image-seq of stromal cells. a) Example of the gating strategy used to isolate CXCl12^+^, CD45^−^ stromal cells from a micropipette sample for cell lysis and SMARTseq-v4 library preparation. Sorted cells were gated on singlet live cells (DAPI-). b) UMAP embedding of the stroma SMARTseq-v4 data using the top 30 principal components, color coded by cell population. c) Heatmap of scaled normalized expression for the 20 most highly expressed genes within each cell cluster. d) Heatmap of scaled normalized expression for Lepr-MSC and Endothelial cell marker genes from the literature^27^. UMAP: uniform manifold approximation and projection. MSC: Mesenchymal stromal cells, EC: endothelial cells. Source data

To demonstrate the technology’s ability to obtain spatially resolved single-cell transcriptional data of stromal cells (Extended Data Fig. 6), we isolated 43 CD45^−^CXCL12^+^ cells by micropipette and flow cytometry. After library preparation and sequencing using SMARTseq-v4, we were able to construct a shared nearest neighbors graph and UMAP embedding of these cells (Extended Data Fig. 6b), and identified two separate clusters. We annotated these as mesenchymal stromal and endothelial cells (Extended Data Fig. 6d) based on literature marker genes^27^. A heatmap of differentially expressed genes within these clusters is shown in Extended Data Fig. 6c.

To further validate the spatial selectivity of our technology we imaged an MLL-AF9 mouse model of AML in which leukemia cells express GFP under the ubiquitin promoter at day 10 after transplantation. As shown previously^28^, we found regions of high leukemic burden to be interspersed with regions of low leukemic burden (Fig. 3g). We used Image-seq to isolate cells from a high-burden region and a low-burden region, and processed them separately for scRNA-seq using the 10x Chromium protocol (see Methods for details). UMAP embeddings of both samples show that the high-burden sample was composed primarily of AML cells, whereas the low-burden sample did not include leukemia cells (Fig. 3h). Furthermore, strong levels of *Gfp* expression were detected in the AML cell cluster from the high-burden sample (Fig. 3g), highlighting the technique’s spatial selectivity and high sensitivity.

#### Image-seq analysis of early leukemia expansion

**Fig. 4:**
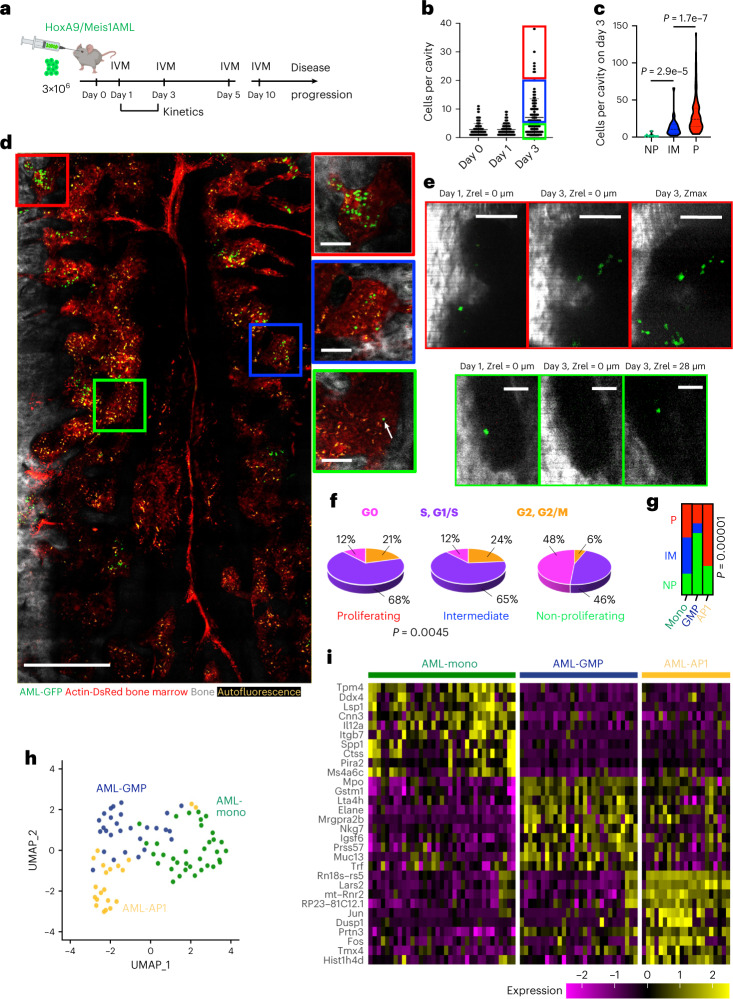
Early leukemia progression by Image-seq. **a**, In vivo imaging timepoints. IVM, intravital microscopy. **b**, Cell counts per bone marrow cavity on day 0 (*n* = 143 cavities, *n* = 6 independent mice), day 1 (*n* = 103 cavities, *n* = 6 independent mice) and day 3 (*n* = 132 cavities, *n* = 7 independent mice) after transplantation. Error bars represent the standard deviation and are centered at the arithmetic mean. The 99.9% confidence interval for proliferating (P) cells is shown in red, that for intermediate (IM) cells in blue and that for non-proliferating (NP) cells in green. **c**, Number of cells per cavity on day 3 plotted for P, IM, and NP regions. The dashed line represents the median and the dotted line, the quartiles in each distribution. Statistical significance was determined using Welch’s ANOVA test and the adjusted *P* values were determined using Dunnett’s multiple comparisons test. NP, 10 biological replicates, *n* = 3 independent mice; IM, 32 biological replicates, *n* = 3 independent mice; P, 107 biological replicates, *n* = 3 independent mice. **d**, Tiled, maximum intensity projection image of leukemic bone marrow on day 3 after transplantation. Red square and inset, P cells; blue square and inset, IM cells; green square and inset, NP cell (marked by white arrow). Scale bars: main image, 500 μm; insets, 100 μm. Bone, bone SHG. Imaging of the entire calvarium bone marrow at day 3 was performed in 17 independent mice, including 122 biological replicates with P, 35 biological replicates with IM, and 79 biological replicates with NP cells. **e**, Images of proliferating (top, red border; scale bars, 100 μm) and non-proliferating (bottom, green border; scale bars, 50 μm) AML cells imaged longitudinally in the same animal and bone cavity on day 1 and 3 after transplantation. Bone SHG signal is shown in grey, AML-GFP signal is shown in green. The same depth along the z dimension is shown (Zrel = 0) on both days, along with a maximum intensity projection for P cells. For the NP cell a single Z slice (Zrel = 28 µm) shows that the AML cell has moved deeper into the bone marrow on day 3. A total of 107 biological replicates with P, 10 biological replicates with NP and 32 biological replicates with IM cells (*n* = 3 independent mice, respectively) were identified by longitudinal imaging (definition of P, NP, IM based on the fold-change difference between day 1 and day 3). **f**, Distribution of the cell cycle in the three imaging phenotypes (*P* value obtained using a two-sided Fisher’s exact test). **g**, Bar-plot representing the fraction of P, NP, and IM cells in each AML cluster. AP1, AML-AP1; GMP, AML-GMP; Mono, AML-mono (*P* value obtained using a two-sided Fisher’s exact test). **h**, Leiden clustering of 84 AML cells isolated by Image-seq after regression of cell cycle genes using Seurat, visualized as UMAP embedding. **i**, Heatmap of scaled normalized expression for the 10 most strongly upregulated genes (based on logFoldChange) in each AML cluster. Source data

**Extended Data Fig. 7:**
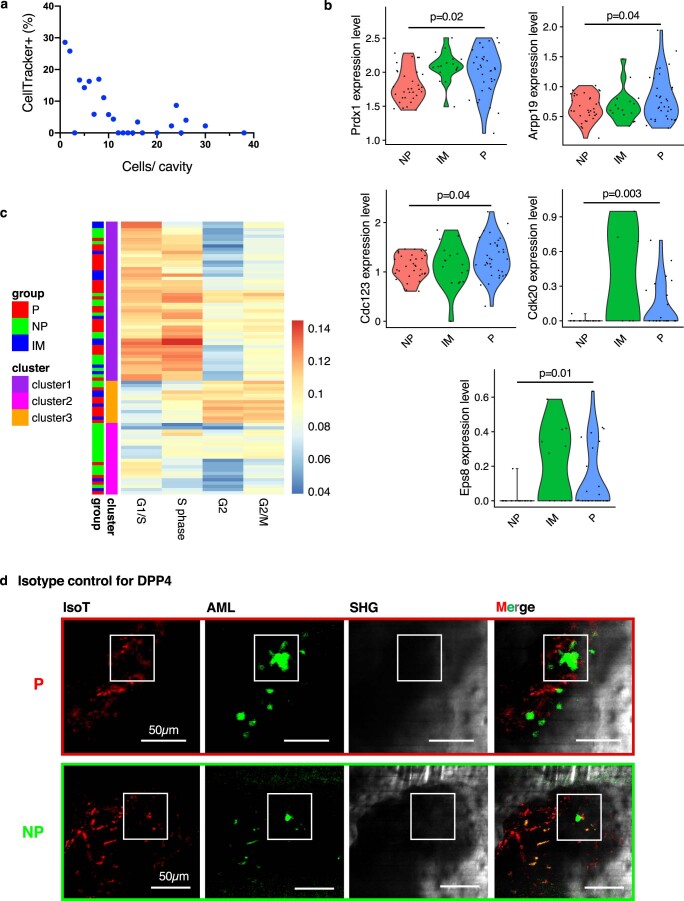
Cell cycle analysis of AML cells from Fig. 4, along with selected intravital microscopy data. a) Percentage of AML cells that are CellTracker Red+ on day 3 (CellTracker + (%)) as a function of the number of cells per cavity. b) Violin plots of selected cell cycle relevant genes from Supplementary Table 4. Statistical significance was determined using a two-sided Student’s t-test (Sample size: NP = 33, IM = 17, P = 34 cells, 11 independent mice). c) Heatmap showing scaled average expression of gene sets in distinct phase of cell cycle, each column indicates a cell. AML cells are clustered into three groups by hierarchical clustering based on cell cycle genes. d) Representative images of P and NP cells from mice injected with rat IgG2a,κ-AF568 antibody (DPP4 isotype control). Imaging of DPP4 isotype control labeling was performed in 14 biological replicates with NP cells and 6 biological replicates with P cells (1 independent mouse). Definition of P/ NP/IM based on cell number at day 3. Source data

We next turned our focus to early leukemia progression in a Hoxa9/Meis1-Ubiquitin-c-GFP (HA9M1) mouse model of AML, performing intravital imaging of the calvarium bone marrow between 1 and 3 days after transplanting 3 × 10^6^ cells into non-irradiated recipients (Fig. 4a). We found that starting from day 3, the number of leukemia cells per bone marrow cavity (concave endosteum) rapidly increases (Fig. 4b), and chose this timepoint to study the mechanisms of early leukemia expansion. We used longitudinal imaging of the same cavities in the same animals to quantify proliferation dynamics (see Fig. 4e for examples of longitudinal images) and classify cells as either proliferating (P, average of 30 cells per cavity on day 3), intermediate (IM, average of 13 cells per cavity on day 3) or non-proliferating (NP, average of 2 cells per cavity on day 3), as shown in Fig. 4c, where the median and quartile of the distributions are given (see Methods for details). We then used the 99.9% confidence interval of AML cells per cavity on day 3 to identify sites for cell extraction and sequencing. Prior to the sequencing experiments we quantified label dilution on day 3 after transplantation with CellTracker-labeled AML cells. The percentage of CellTracker-positive cells per bone marrow cavity decreased as the overall number of cells increased (Extended Data Fig. 7a), further verifying our imaging-based classification. Surprisingly, these proliferative phenotypes were distributed throughout the calvarium bone marrow in a spatially heterogeneous manner (Fig. 4d), further underlining the need for a spatially resolved analysis by scRNA-seq.

We identified P, NP and IM AML cells by imaging and aspirated them by micropipette using our Image-seq platform. Subsequently, we separated the AML cells from the ~100–400 surrounding hematopoietic cells by sorting them into individual wells of a 96-well plate by flow cytometry and gating for GFP (see Supplementary Fig. 1 for examples of the gating strategy used to isolate an NP, as well as several P cells, from a single micropipette sample). By choosing this strategy (shown schematically in Fig. 3a as steps 1, 2, 3, 4 and 5b), we optimized the yield of the rare AML cells and avoided the ~30–40% cell loss that occurs during cell encapsulation using the 10x Genomics platform. In total, we collected ~40 cells from four different locations in the P category, ~40 cells from ~30 different locations in the NP category, as well as ~20 cells from three different locations in the IM category from *n* = 11 mice. Experimental throughput was limited by the extremely rare NP cells, which made up only 0.7% of AML cells on day 3, with the overall leukemic burden already <0.01%. To minimize the issue of dropout that is often observed in scRNA-seq data, we used the SMARTseq-v4 protocol for reverse transcription and library construction. Because of the SMARTseq protocol’s unique chemistry, the number of genes and transcripts detected per cell are higher than for any other scRNA-seq technology (Extended Data Fig. 5d and ref. ^29^). Used in combination with the SMARTseq-v4 protocol, Image-seq therefore has the highest sensitivity and transcript coverage of any currently available spatial sequencing technology, with an average of 3,314,087 total mapped reads or 8,053 genes per cell. Of the 97 cells that were sequenced, a total of 84 cells passed quality control.

After sequencing and read alignment we identified cells that were in the G0 phase by adapting methodologies from refs. ^30,31^ and performed hierarchical clustering based on cell cycle genes, identifying three separate clusters (shown in the heatmap of Extended Data Fig. 7c). Cluster 1 was enriched in genes related to the S and G1/S phases, cluster 3 was enriched in genes related to the G2 and G2/M phases, and cluster 2 lacked expression of cycle genes and was therefore classified as quiescent cells (G0 phase). Interestingly, the distribution of cell cycle in the P and IM populations was very similar, with the largest proportion of cells in the G1/S and S phases, and the second largest proportion in G2 and G2/M (Fig. 4f). For the NP population, however, the major contribution came from the G0 phase, and the second largest from the G1/S and S phases. These results highlight the validity of our imaging-based classification, as well as our technology’s ability to distinguish subtle phenotypic differences through imaging.

**Extended Data Fig. 8:**
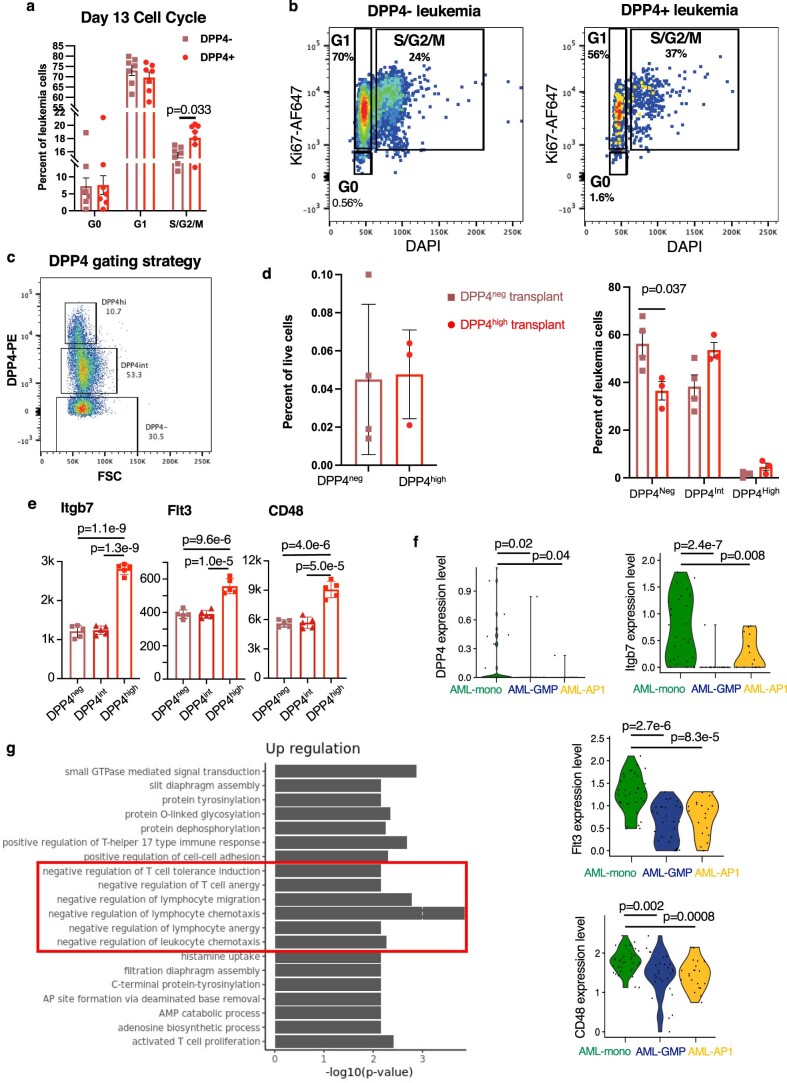
Gating strategy and DPP4-correlated genes by flow cytometry. a) Ki-67 flow cytometry analysis of HA9M1 cells 13 days after transplantation (N = 7 biological replicates, 2 independent experiments). Statistical significance was determined by unpaired, two-sided Student’s t-test, error bars denote standard error of the mean. b) Ki-67 cell cycle gating strategy. c) Gating strategy used for identifying DPP4*high*, DPP4*int* and DPP4*neg* cells. d) (Left) Day 10 leukemia burden in mice transplanted with 1,000 DPP4high (N = 3) or DPP4neg (N = 4) HA9M1 cells (2 independent experiments). Error bars denote standard deviation. (Right) Percentage of DPP4high-int-neg cells isolated from recipients transplanted with DPP4high (N = 3, red bars) or DPP4neg (N = 4, beige bars) HA9M1 cells (2 independent experiments). An unpaired, two-sided Student’s t-test was used for statistical analysis, error bars denote standard deviation. e) Mean fluorescence intensity of Itgb7, Flt3 and CD48 detected in DPP4*high*-*neg* HA9M1 cells by cell surface staining and flow cytometry 3 weeks after transplantation (N = 5 biological replicates, 2 independent experiments). Statistical significance was determined by one-way ANOVA followed by Tukey’s post-hoc test, error bars denote standard deviation. f) DPP4, Itgb7, Flt3 and CD48 expression level for the AML clusters from Fig. 4h. A two-sided Wilcoxon rank sum test was used for statistical analysis (AML-AP1 N = 21, AML-GMP N = 28, AML-mono N = 35 cells, 11 independent mice). g) GO analysis of top 300 up-regulated genes comparing P and NP cells. The statistical analysis was performed by hypergeometric test. Terms related to the inhibition of T cells are highlighted by the red rectangle. HA9M1: Hoxa9/Meis1-Ubiquitin-c-GFP. GO: gene ontology. Source data

Next, AML cells were sub-clustered after regressing out cell cycle genes using Seurat^32^, with the resulting UMAP embedding shown in Fig. 4h. Three major clusters were observed, and the heatmap in Fig. 4i shows the top 10 differentially expressed genes in each of them. The dark-green cluster (AML-mono) was enriched in progenitor cell markers of the monocytic lineage such as *Ctss*, *Cnn3*, *Ms4a6c*, *Pira2* and *Lsp1* (refs. ^3,26,33^), as well as *Itgb7* and *Flt3* (Fig. 4i, Extended Data Fig. 8f and Supplementary Table 2), which have been associated with stronger leukemia-initiating capacity in Hoxa9/Meis1 AML^34^. AML-GMP expressed granulocyte–monocyte progenitor markers such as *Mpo*, *Elane*, *Prss57* and *Ctsg*^22,26,33^, along with *Gstm1*, which has been associated with quiescent, therapy-resistant, leukemia-initiating cells in AML^35–37^. AML-AP1 expressed *Prtn3*, which is a marker of the monocytic lineage^22^, along with *Jun* and *Fos*, which comprise the AP1 transcription complex^38^, as well as *Dusp1*, which are all part of the MAP-kinase signaling pathway (https://www.kegg.jp/pathway/mmu04010) and are associated with cell proliferation. Cluster AML-GMP consisted primarily of cells that were classified as NP by imaging, AML-AP1 consisted primarily of P cells, and AML-mono consisted primarily of P and IM cells (a detailed breakdown is given in Fig. 4g).

#### Increased proliferation of DPP4-positive cells in mouse and human AML

**Fig. 5:**
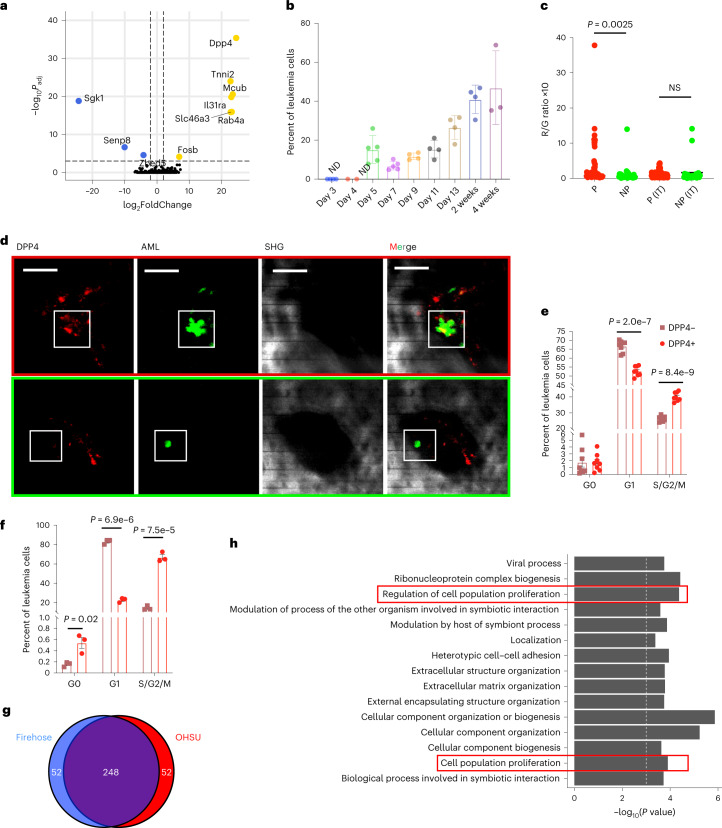
DPP4 is the most strongly differentially expressed gene between P and NP cells. **a**, Differentially expressed genes between P and NP cells calculated using DESeq2 (ref. ^60^), which determines statistical significance using the Wald test and uses an interpretation of the Benjamini–Hochberg method to control the false discovery rate. The vertical dashed lines show the cut-off for gene filtering (log_2_FoldChange 2 and −2) and the horizontal dashed line signifies an adjusted *P* value of 0.01. **b**, Percentage of leukemia cells expressing DPP4 during disease progression, as analyzed using cell surface staining and flow cytometry. DPP4^+^ cells are defined as DPP4high and DPP4int cells according to the gating strategy in Extended Data Fig. 8c (day 4 *n* = 2, day 3 and 4 weeks *n* = 3, days 9–13 and 2 weeks *n* = 3, days 5–7 *n* = 5 biological replicates). Error bars denote standard deviation. Data were collected from three independent experiments. **c**, DPP4 antibody labeling detected using intravital microscopy after i.v. injection of DPP4-AF568 antibody. Ratio of red (that is, DPP4-AF568) to green (that is, AML-GFP) fluorescence is plotted for P (5 biological replicates) and NP cells (25 biological replicates), as well as control P (6 biological replicates) and NP (14 biological replicates) cells labeled with isotype antibody (IT). Statistical significance was determined using a two-tailed Mann–Whitney test. Data were collected from three independent mice. **d**, Examples of intravital multiphoton microscopy images used for the analysis in **c**. Red (DPP4-AF568), green (HA9M1-GFP), gray (bone SHG signal) channels and merged images are shown at a single z plane. Images of P cells have red borders; images of NP cells have green borders. Scale bars, 50 μm. Imaging of DPP4 antibody staining: 25 biological replicates with NP cells and 5 biological replicates with P cells (two independent mice). For **b** and **d** the definition of P/NP/IM is based on cell number at day 3. **e**,**f**, Cell cycle analysis on day 7 after transplantation for DPP4^+^ and DPP4^−^ AML cells by Ki-67 staining in HA9M1 cells (**e**) and an MLL-AF9 leukemia model (**f**) (HA9M1, *n* = 8; MLL-AF9, *n* = 3 biological replicates). Statistical significance was determined by unpaired, two-sided Student’s *t*-test, and the error bars denote s.e.m. Data were collected from two independent experiments. **g**, Overlap of the top 300 DPP4-correlated genes in the Oregon Health and Science University (OHSU)^48^ and Firehose^49^ datasets plotted as a Venn diagram. **h**, Top GO terms for overlapping genes from **g**. The statistical analysis was done using a hypergeometric test. HA9M1, Hoxa9/Meis1-Ubiquitin-c-GFP. Source data

With the goal of identifying signals that trigger the exit of leukemia-initiating cells from the quiescent state, we investigated the differential expression of genes between P and NP cells (Fig. 5a and Supplementary Table 3) without prior regression of cell cycle genes. GO analysis identified several terms related to the negative regulation of immune response (Extended Data Fig. 8g). A list of cell cycle-related genes that were differentially expressed is given in Supplementary Table 4. The most strongly differentially expressed gene, both when comparing P with NP and the entire population of P + IM with NP cells (Supplementary Table 5), was *Dpp4*. DPP4 or CD26 is a transmembrane protein that functions as a serine protease, selectively cleaving the amino-terminal, penultimate proline, or alanine of proteins^39–41^. Among its many substrates are granulocyte–macrophage colony-stimulating factor, CXCL12, granulocyte colony-stimulating factor, interleukin 3 and erythropoietin, which are known to enhance proliferation, survival, chemotaxis, homing and engraftment of hematopoietic stem and progenitor cells^39,42^. DPP4 has also been shown to cleave CCL2, CCL3, CXCL6, CXCL9 and CXCL10, which play a role in decreasing the survival and proliferation of hematopoietic stem and progenitor cells. Several studies have implicated DPP4 as a leukemic stem cell marker in chronic myeloid leukemia^43–45^, and recent studies of human AML have linked increased DPP4 expression to a poorer overall survival^46^ and have associated AML-derived DPP4 with the suppression of normal hematopoietic progenitor cell proliferation^47^. However, DPP4 was previously not known to play a role in early AML proliferation.

To validate *DPP4* expression we assessed its protein expression using flow cytometry and found that it increases as a function of disease progression, with a maximum stable expression at ~50% by week 4 (Fig. 5b). Although DPP4 expression was too low to be detected using flow cytometry on day 3, it could be detected using in vivo imaging, and we found a statistically significant difference when DPP4 antibody labeling was compared in P versus NP cells whereas we did not observe this in the corresponding isotype control (Fig. 5c,d and Extended Data Fig. 7d). Next, we analyzed the cell cycle in DPP4-positive versus DPP4-negative AML cells using Ki-67 staining (see Extended Data Fig. 8b for gating strategy), which confirmed that DPP4-positive cells have a higher cycling rate (Fig. 5e and Extended Data Fig. 8a). To elucidate whether this phenotype is more broadly observed, we studied an MLL-AF9 model of AML and found the decrease in the G1 phase and the concomitant increase in the S, G2 and M phases in DPP4-positive cells to be even more pronounced (Fig. 5f). Note that at day 13 (Extended Data Fig. 8a) the difference in cycling rate is less pronounced than at day 7, and there is no difference in the proportion of cells in the G1 phase. This is a very aggressive model and even at day 13 more than 80% of the marrow is composed of leukemia cells, suggesting that the cells are running out of space to proliferate, which is likely to reduce the cycling rate. In keeping with this, the overall proportion of DPP4-positive and DPP4-negative cells that are actively cycling is considerably lower on day 13 than on day 7 (*P* < 0.0001), which is likely the reason that the proportion of DPP4-positive cells reaches a maximum stable value of 50%. We additionally analyzed DPP4-correlated genes in two published, human AML datasets^48,49^ (see Methods for details) and observed a high overlap between the top 300 positively correlated genes. GO analysis of the overlapping gene set (Fig. 5g) showed an enrichment of terms related to cell cycle (Fig. 5h), suggesting that the phenotype is relevant to human AML.

**Extended Data Fig. 9:**
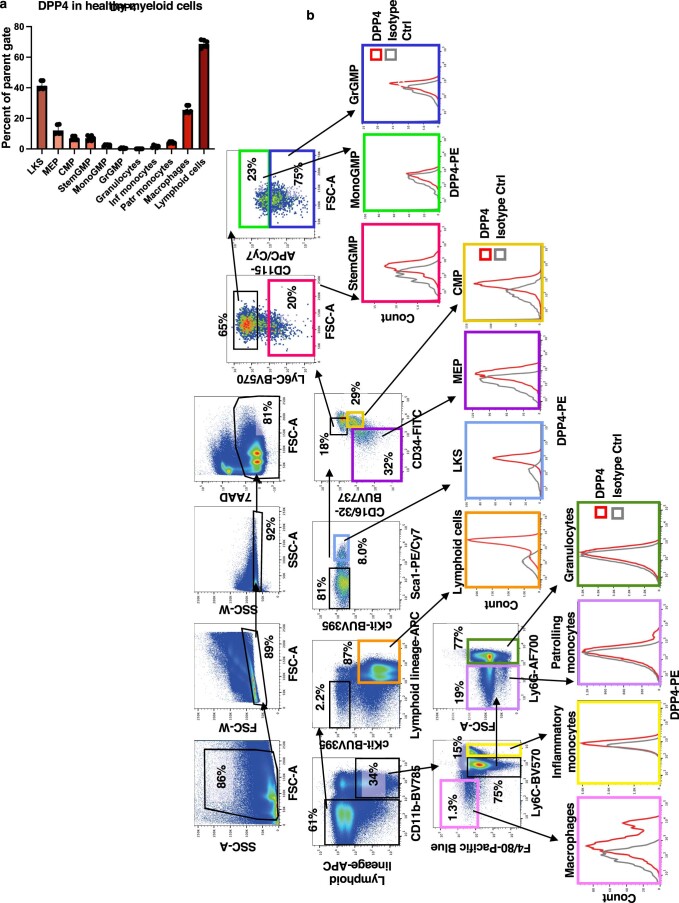
Flow cytometry analysis of DPP4 in healthy myeloid precursors. a) Percentage of DPP4 + cells in various myeloid cell populations (N = 6 biological replicates). Error bars denote standard deviation. Data were collected from two independent experiments. LKS, Lineage- cKit+ Sca-1+; MEP, megakaryocytic-erythroid progenitor; CMP, common myeloid progenitor; StemGMP, Stem-like granulocyte-monocyte progenitor; MonoGMP, monocytic granulocyte-monocyte progenitor; GrGMP, granulocytic granulocyte-monocyte progenitor; Inf monocytes, inflammatory monocytes; Patr monocytes, patrolling monocytes. b) Gating strategy for the myeloid cell populations from (a), along with DPP4 and isotype count data for each cell type. Analyzed cells were gated on singlet live cells and percentages refer to percent of parent gate. Gray curves represent the Isotype control, red curves DPP4. Source data

Because DPP4 expression was confined mainly to the AML-mono cluster, which expressed high levels of *Itgb7*, *Flt3* and *Cd48* (Extended Data Fig. 8f), we analyzed their cell surface expression using flow cytometry. We found a strong increase in Itgb7 expression, as well as a moderate increase in Flt3 and CD48 for cells that had high DPP4 expression as compared with negative and intermediate DPP4 expression (Extended Data Fig. 8c,e), suggesting that this combination of cell surface markers can be used to identify cells belonging to AML-mono. Interestingly, overexpression of Flt3 has been associated with poor prognosis in AML^50^, and combined expression of Itgb7 and Flt3 has been used to identify leukemic stem cells in Hoxa9/Meis1 AML^34^. We investigated DPP4 expression in healthy myeloid precursors, as well as lineage-specified cells, using flow cytometry (Extended Data Fig. 9a). We found higher DPP4 levels on cells that are more immature (with the exception of macrophages and lymphoid cells), which supports the notion that DPP4 expression is not due to progression along the myeloid differentiation trajectory in AML cells, or associated with priming towards a specific lineage.

To assess leukemia-initiating capacity, we isolated 1,000 DPP4high and DPP4neg AML cells 3 weeks after transplantation using flow cytometry. We transplanted them into fresh, non-irradiated recipients and compared leukemic burden and the proportion of DPP4-positive and DPP4-negative cells at day 10 (Extended Data Fig. 8d). Interestingly, a subset of DPP4-negative cells converted to being DPP4 positive and vice versa, suggesting that DPP4 expression could be induced by the microenvironment.

#### Activation of DPP4 expression by the microenvironment

**Fig. 6:**
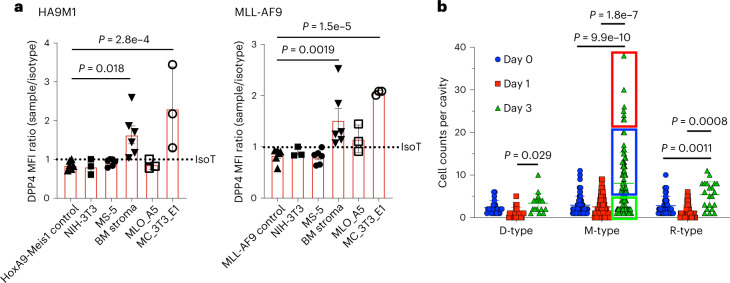
The leukemia microenvironment. **a**, DPP4 expression on AML cells, analyzed by cell surface staining and flow cytometry, after 3 day co-culture with stromal cells. BM stroma, immortalized bone marrow stroma from D.B.S.’s laboratory; MC_3T3_E1, osteoblast precursor cell line; MLO_A5, murine long bone osteocyte cell line; MS-5, mouse stroma cell line; NIH-3T3, fibroblast cell line. (Control, NIH-3T3, MS-5 and BM stroma, *n* = 6; MC_3T3 and MLO_A5, *n* = 3 biological replicates; two independent experiments). Statistical significance was calculated using one-way ANOVA followed by Šídák’s multiple comparisons test. Error bars represent the standard deviation and are centered at the arithmetic mean. **b**, Distribution of HA9M1 cells in D, M and R cavities on day 0, 1 and 3 after transplantation. Day 0: D0, *n* = 22 cavities, M0 *n* = 82 cavities, R0 *n* = 41 cavities (6 independent mice); day 1: D1 *n* = 21, M1 *n* = 84, R1 *n* = 43 cavities (6 independent mice); day 3: D3 *n* = 16, M3 *n* = 95, R3 *n* = 21 cavities (7 independent mice). Error bars represent the standard deviation and are centered at the arithmetic mean. Statistical significance was determined using an unpaired, two-sided Mann–Whitney *U*-test. The 99.9% confidence intervals for P, IM and NP cells from Fig. 4b,c are marked by red, blue and green rectangles, respectively. HA9M1, Hoxa9/Meis1-Ubiquitin-c-GFP; IsoT, isotype; MFI, mean fluorescence intensity. Source data

**Extended Data Fig. 10:**
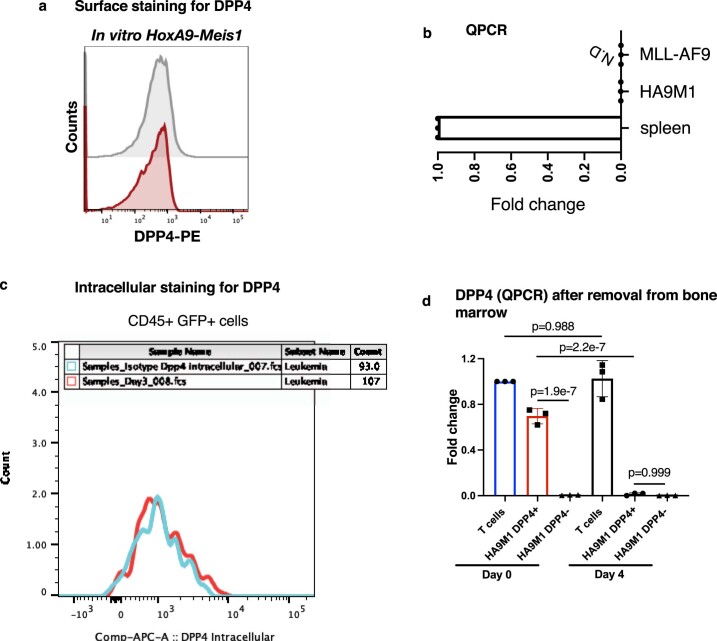
Detection of DPP4 on in vitro cultured cells. a) Cell surface antibody labeling with DPP4 in HA9M1, analyzed by flow cytometry. b) Analysis of *Dpp4* expression by QPCR on *in vitro* cultured HA9M1 and MLL-AF9 cells. Results are compared to splenic T cells, which express reproducible levels of *Dpp4*. (N = 3 biological replicates from 2 independent experiments). Error bars denote standard deviation. c) Flow cytometry analysis of DPP4 expression in HA9M1 by intracellular staining with DPP4 antibody. d) Analysis of *Dpp4* by QPCR on AML cells collected from the bone marrow 3 weeks after transplantation. DPP4^+^ and DPP4^−^ HA9M1 cells were sorted by flow cytometry and their gene expression compared to that of splenic T cells by Q-PCR. After initial analysis, the T cells and DPP4^+ /−^ HA9M1 cells were cultured for 4 days using the same media and analyzed once again for *Dpp4* expression. HA9M1: Hoxa9/Meis1-Ubiquitin-c-GFP (N = 3 biological replicates from 2 independent experiments). Statistical significance was calculated by one-way ANOVA followed by Sidak’s post-hoc test, error bars denote standard deviation. Source data

While observing DPP4 cell surface expression to be highly stable and reproducible in vivo, we were not able to detect DPP4 on AML cells cultured in vitro (Extended Data Fig. 10a). We were also unable to detect DPP4 by intracellular staining or quantitative polymerase chain reaction (qPCR) (Extended Data Fig. 10b,c). In addition, we found that although DPP4-positive HA9M1 cells expressed *Dpp4* at comparable levels to splenic T cells immediately following removal from the bone marrow environment (day 0 qPCR results in Extended Data Fig. 8d), they subsequently lost all *Dpp4* expression at the transcriptional level after 4 days of in vitro cell culture. Remarkably, co-culture of AML cells with bone marrow stromal and osteoblast precursor cells activated DPP4 cell surface expression, while co-culture with other stromal cell lines did not (Fig. 6a). Combined with the observation that DPP4 expression is upregulated in specific bone marrow compartments (Fig. 5a,c), the data suggest that different niches drive the upregulation of DPP4. Given the recent finding that hematopoietic stem cell proliferation is restricted to a subset of bone marrow cavities with mixed bone remodeling activity (M-type cavities)^51^ we reasoned that these niches could drive proliferation of AML cells. We used the dye labeling strategy developed in ref. ^51^ to distinguish deposition (D-type), resorption (R-type) and mixed (M-type) bone marrow cavities by in vivo imaging, finding that P cells are exclusively located in M-type cavities (Fig. 6b).

## Discussion

AML is an aggressive blood cancer characterized by an accumulation of immature myeloid cells in the bone marrow that are arrested in differentiation and that accumulate as immature and malignant self-renewing progenitors. Despite an initially favorable response to treatment with intensive cytotoxic chemotherapy, ~75% of patients die within 5 years of diagnosis^52^. Relapse is thought to be driven by the rare pool of leukemia-initiating cells that persist in the bone marrow following chemotherapy^53^. Arguing that an important approach to the development of new therapeutics is the targeting and exploiting of the supportive interaction as leukemia-initiating cells communicate with and seek refuge within the bone marrow microenvironment, we decided to study and identify signals that trigger the exit of leukemia-initiating cells from the quiescent state, thereby gaining mechanistic insight into disease recurrence. We further reasoned that examining the bone marrow at a stage of very low leukemic burden, comparable to the state of minimal residual disease (when the AML cells are more likely to be surrounded by normal hematopoietic and stromal cells), provides unique insights that cannot be obtained using traditional assays of relatively high leukemic burdens in which the bone marrow is crowded with malignant cells and the microenvironment is severely altered.

Here, we have shown how the combination of intravital microscopy to study the dynamics of AML disease progression, and spatially resolved scRNA-seq, provides new insights into leukemia biology. Moreover, multiphoton microscopy uniquely informs the 3D spatial context of AML cells and can be used to validate the single-cell gene expression data in a spatially resolved manner (Fig. 5c,d and Extended Data Fig. 7d), as well as characterize different microenvironments (Figs. 4b–e and 6b) that can be analyzed for their differential expression (Fig. 5a). We found that DPP4 marks a more proliferative phenotype both in murine and in human disease, which makes it a promising target for further investigations that could lead to new treatments.

In situ imaging of murine bone marrow has led to numerous insights into the basic biology of hematopoietic stem cells, as well as the spatial organization of bone marrow tissue^16,54,55^. In the skull bone marrow, intravital microscopy has been used for the study of hematopoiesis, along with hematopoietic stem cell and leukemia biology^28,51,56,57^. It has elucidated the temporal dynamics of these processes and tracked the association of individual cells with distinct bone marrow components in real time^51,58,59^. Imaging alone, however, fails to provide unbiased mechanistic insight into the observed cellular dynamics and spatial organization. It is precisely such information, however, that promises to bring new insights into hematopoietic and leukemia biology and, concomitantly, the development of new therapeutics^1^.

To date, the only spatially resolved transcriptional study of the murine bone marrow, a tissue that remains highly challenging to section, relied on bulk transcriptomic analysis of tissue blocks that were cut from formalin-fixed bone marrow sections using laser micro-dissection^3^. The cellular composition of individual blocks was then inferred computationally using a separate, scRNA-seq dataset. To our knowledge there have been no spatially resolved transcriptional studies of the leukemic bone marrow.

Image-seq represents a new experimental approach for integrating spatial and molecular information. Multiple contrast mechanisms can be used to visualize the procedure and reconstruct the 3D spatial position of the extracted cell sample, including autofluorescence, confocal reflectance (Fig. 1c and Supplementary Video 1) or labeling with a fluorescent membrane dye such as Di-8-ANEPPS (used in the experiments in Figs. 2d and 3). When combined with intravital microscopy, Image-seq additionally enables transcriptomic analysis on cells for which the position and history have been documented by intravital microscopy. Because of its utility both for high-throughput sequencing and in-depth profiling of rare cells, the Image-seq platform is highly versatile in its applications. We additionally expect that it can be applied to study fixed tissues and tissue sections (for example, using the 10x Genomics protocol for fixed tissues instead of the protocol we detailed here), although we recommend working with live cell samples when possible to achieve the highest sensitivities and transcript coverage. Currently there is a trade-off between spatial resolution and throughput. Although it is possible to isolate very few cells and obtain a spatial resolution of ~30 × 30 × 30 µm, or even a single cell if there is only one fluorescent cell in the isolated tissue volume (Fig. 1c) that can be sorted by flow cytometry, this comes at the cost of lowered throughput. Increasing the number of extracted cells, however, lowers spatial resolution. As we expand the technology we expect to overcome these shortcomings and refine its resolution and increase its throughput. We anticipate that Image-seq will be used to investigate a range of diseases and biological questions involving the bone marrow, along with other tissues that remain difficult to section. Furthermore, based on the technology’s ability to isolate viable cells, we anticipate its applicability to spatially resolved multi-omics studies in a range of biological settings.

## Methods

A detailed protocol of the Image-seq procedure is available in the Supplementary Information.

### Microscope

Intravital microscopy and plasma-mediated laser ablation were performed using a custom-built multiphoton microscope. The output of a femtosecond, 1,550 nm fiber laser (Calmar Cazadero) operating at a repetition rate of 5 MHz was split into two optical paths: one was frequency doubled with a BiBO crystal (Newlight Photonics) to obtain a wavelength of 775 nm that was used for either imaging or ablation (Extended Data Fig. 1). The other was coupled into a large mode-area photonic crystal fiber (LMA-35, NKT Photonics) where, by soliton self-frequency shift, radiation with a frequency tunable between 1,880 nm and 1,960 nm was generated. This was frequency doubled (BiBO, Newlight Photonics) to obtain imaging wavelengths between 940 and 980 nm. The imaging and ablation beams were spatially overlapped using a dichroic mirror (Zt 785 RDC, Chroma) and directed to the scanning optics, which consisted of a revolving polygon (36 facets, 14,400 r.p.m., *x* scan) conjugated to a scanning galvanometer mirror (15 Hz, *y* scan), and which were conjugated to the back aperture of the objective lens (Olympus, ×60, numerical aperture (NA) 1.0, water immersion). This served to steer the imaging and ablation beams across the microscope field of view. Typical pulse energies used for imaging were between 1 and 2 nJ and typical pulse energies for ablation were between 10 and 15 nJ. For stable plasma generation a continuous stream of PBS (ThermoFisher Scientific) was flushed across the sample at a rate of 10 ml min^−1^ to remove any gas or debris generated during the ablation procedure. The sample (mouse) was positioned in the image plane using a micromanipulator (Sutter instruments) and translated along the z dimension to image or ablate a 3D volume of tissue. Fluorescence signals were collected after excitation with 775 nm or 980 nm laser light, using three photomultiplier tubes (PMTs). The typical configuration of the dichroic mirrors was FF705 LP, FF495 LP and FF552 LP, with filters 439/154 (blue PMT R7600-U300, Hamamatsu) for the detection of collagen second-harmonic generation signal from bone, 525/50 (green PMT R7600-U300, Hamamatsu) for the detection of AML cells and tetracycline, and 607/70 (red PMT R7600-U20, Hamamatsu) for the detection of CellTracker Red and Di-8-ANEPPS (ThermoFisher Scientific) signals. An avalanche photodiode was also installed for collection of the confocal reflectance signal (used to visualize the in vivo cell isolation procedure in Supplementary Video 1).

### Procedure for intravital imaging

The procedure for intravital imaging of the calvarium bone marrow is described in detail elsewhere^61^. Prior to intravital microscopy and ablation, analgesics were given (buprenorphine at 0.05–0.1 mg kg^−1^ i.p.), mice were anesthetized using vaporized isoflurane (3–4% for induction, 1–2% for maintenance) and depth of anesthesia ensured by toe pinch. Hair around the incision site on the scalp was trimmed, and skin was made aseptic using a betadine scrub. The incision (~5 mm × 7 mm) was made using sterile surgical scissors, and the skin folded back to expose the skull bone, which was hydrated using sterile PBS. Mice were transferred to a mouse holder with integrated heating pad (37 °C), and a continuous stream of isoflurane supplied via a nose cone during in vivo imaging and ablation. Intravital microscopy experiments were carried out using the microscope and dichroic mirror–filter configuration detailed above, and image stacks were acquired with a 2 μm step size from the calvaria surface and by averaging 15 frames to obtain a single image. At the end of each imaging session the mice were either sacrificed or survival surgery was performed. For survival, the exposed skull was extensively irrigated with sterile saline and the scalp closed with surgical sutures (Ethicon 6-0 nylon monofilament, Ethicon). After closure, 0.25% bupivacaine (2 mg per kg animal weight) was administered to the surgical site via percutaneous infiltration to aid with pain management, and triple antibiotic ointment (Curad) was applied on the sutured area. The animal was returned to its cage and monitored until awake. Buprenorphine (0.05–0.1 mg per kg animal weight) was given i.p. or s.c. along with topical antibiotic ointment every 8–12 h for up to 2 days after surgery.

### In vivo cell aspiration

The site for cell extraction was identified by intravital imaging of the calvarium bone marrow (procedure detailed above). A volume of bone ~40 µm × 200 µm × 300 µm was removed using laser ablation (pulse energy 14 nJ). A circular channel was etched (diameter ~100 µm, depth 20–30 µm, pulse energy 10 nJ) by placing an iris in the intermediate image plane, and a micropipette (MPB-FP-20, Origio) was inserted through the channel and into the bone marrow using a micromanipulator (Sutter Instruments). The target cell was aspirated by suction with an Air Syringe (Cooper Surgical) and the procedure was visualized using a combination of multiphoton and confocal reflectance signals. The ablation procedure itself was performed at a rate of 0.25 µm per 670 ms along the *z* dimension, which corresponded to 10 passes per plane using the 15 frame per second imaging rate of the optical system.

### Image-guided cell isolation for flow cytometry and sequencing

#### In vivo imaging of calvarium

Prior to cell isolation the mice underwent intravital imaging, the sites for cell extraction were identified and their spatial position recorded with respect to the bregma and lambda reference points. Mice were retro-orbitally injected with either Di-8-ANEPPS (1.9 mg kg^−1^) or Brilliant Violet 421 anti-mouse CD31 antibody (1 mg kg^−1^, BioLegend) to aid in the visualization of the cell isolation procedure. Anesthesia was increased to 4%, the mice were transferred to a dissection tray and were transcardially perfused as follows.

1. For the isolation of hematopoietic cells: first with an ice-cold solution of 5 µM EDTA in PBS (ThermoFisher Scientific, flow rate 5 ml min^−1^, total volume 10 ml) and then with ice-cold PBS (ThermoFisher Scientific, no Ca or Mg, flow rate 5 ml min^−1^, total volume 10 ml).
2. For the isolation of stromal cells: first with a 37 °C solution of 5 µM EDTA in PBS (both ThermoFisher Scientific) at a flow rate of 5 ml min^−1^ and with a total volume of 10 ml, and then with an enzymatic digestion buffer at 37 °C (flow rate 5 ml min^−1^, total volume 10 ml). The mice were then incubated at 37 °C for 20 min. The enzymatic digestion buffer consisted of 450 U ml^−1^ Collagenase I (Sigma), 125 U ml^−1^ Collagenase XI (Sigma), 60 U ml^−1^ Hyaluronidase (Sigma) and 60 U ml^−1^ DNase I (Sigma) in 20 ml Medium-199 (Gibco).

Mice were transferred back to the microscope and samples were sequentially isolated from positions marked for cell extraction.

#### In situ imaging of tibia

Mice were perfused first with an ice-cold solution of 5 µM EDTA in PBS (ThermoFisher Scientific, flow rate 5 ml min^−1^, total volume 10 ml) and then with ice-cold PBS (ThermoFisher Scientific, no Ca or Mg, flow rate 5 ml min^−1^, total volume 10 ml). Tibia were then dissected and cleaned, and the tibial bone was thinned to a thickness of ~50 µm using a razor blade. Bones were mounted onto a microscope slide by fastening a piece of modeling clay to the glass slide and gently pressing the bone onto the modeling clay. The mounted bone was then transferred to the microscope for in situ imaging.

#### Image-guided cell aspiration (calvarium and tibia)

In each location the procedure was as follows: first, a volume of bone ~40 µm × 200 µm × 300 µm was removed using a pulse energy of 14 nJ; second, a channel with dimensions ~30 µm × 50 µm × 100 µm was created using a pulse energy of 10 nJ; third, the micropipette was inserted through this channel and positioned next to the target cells; and last, the cells were aspirated using a micropipette (MBB-FP-M-20, Origio) and transferred to an Eppendorf tube filled with 5 µl Medium-199 with 2% v/v FBS. Samples were kept on ice until they were either analyzed using flow cytometry (validation experiments), transferred to the 10x Chromium platform, or sorted into individual wells of a 96-well plate by flow cytometry (for library preparation by SMARTseq-v4). The ablation procedure itself was performed at a rate of 0.25 µm per 670 ms along the z dimension, which corresponded to 10 passes per plane using the 15 frame per second imaging rate of the optical system.

Prior to the experiment, micropipettes were coated with Sigmacote (flowed through the micropipette for 2 min at a rate of 200 µl min^−1^) to prevent cells from adhering to the glass surface, as well as with Qtracker 655 vascular labels (5 µl were pipetted up and down several times) to fluorescently coat the pipette and aid with visualization.

### Collection of whole bone marrow preparations

Calvaria were dissected and cut into smaller pieces. Tibia were dissected and cleaned. To aid in the release of the bone marrow, calvaria bone fragments or whole tibia bones were gently crushed in Medium-199 (Gibco) supplemented with 2% FBS (Gibco). The resulting cell suspension was subsequently passed over a 70 μm cell strainer (BD Falcon).

### Cell lines

#### Syngeneic leukemia model

The HoxA9/Meis1 and MLL-AF9 models have been described in detail elsewhere^62,63^. In brief, the HoxA9/Meis1 AML cell line was generated by retroviral transduction with an MSCV-HoxA9-IRES-Meis1 construct (originally designed by G. Sauvageau) into bone marrow mononuclear cells from a mouse expressing GFP under the control of the ubiquitin, and luciferase under the control of the β-actin promoter. The MLL-AF9 cell line used for the cell cycle experiments was generated by collecting bone marrow from a 5-fluorouracil-treated Cas9-GFP mouse, followed by two consecutive transfections with retroviral MLL-AF9. For both models the cells were transplanted into irradiated recipients, collected from terminally ill animals and re-transplanted into a second set of irradiated recipients, from which GFP-expressing cells were collected close to the disease endpoint. These cells were cultured in RPMI 1640 (Gibco) supplemented with 10% FBS (ThermoFisher Scientific), 100 IU ml^−1^ penicillin (Corning), 100 mg ml^−1^ streptomycin (Corning), 5 ng ml^−1^ interleukin 3 (IL-3, Peprotech), as well as 100 ng ml^−1^ stem cell factor (SCF, Peprotech) for the HoxA9/Meis1, and 20 ng ml^−1^ SCF and 10 ng ml^−1^ IL-6 (both from Peprotech) for the MLL-AF9. Recipient female mice (10–12 weeks old) were injected with 3 × 10^6^ cells in 200 µl PBS (HoxA9/Meis1) and 1 × 10^6^ cells in 200 µl PBS (MLL-AF9).

The MLL-AF9 model used for the experiments in Fig. 3 was generated by crossing MLL-AF9 knock-in mice^64^ with mice expressing GFP under the control of the ubiquitin, and luciferase under the control of the β-actin promoter. The bone marrow from a terminally ill male mouse was collected and cultured in vitro using RPMI 1640 (Gibco) supplemented with 10% FBS (Gibco), 100 IU ml^−1^ penicillin, 100 mg ml^−1^ streptomycin (both from Corning), 20 ng ml^−1^ recombinant mouse SCF (rmSCF), 10 ng ml^−1^ recombinant mouse IL-3 (rmIL-3) and 10 ng ml^−1^ rmIL-6 (all from R&D Systems). Recipient male mice (8–10 weeks old) were injected with 1 × 10^6^ cells in 100 µl saline.

For transplantation of DPP4-negative and DPP4-positive HoxA9/Meis1 leukemia, cells were isolated from the long bones and vertebral column 3 weeks after transplantation. The marrow underwent density gradient centrifugation (Ficoll-Paque Plus, Cytiva Life Sciences) at 400 ×*g* for 25 min at room temperature with no brake. The mononuclear layer was isolated and subsequently blocked in PBS with 2% FBS and murine Fc Block (BD Biosciences, dilution 1:50). Following this, the samples were stained with CD45-APC/Cy7 (BD Biosciences, dilution 1:100) and DPP4-PE (Biolegend, dilution 1:20). To exclude dead cells, samples were incubated with 7-aminoactinmycin D (7AAD, 0.25 µg, BD Biosciences) and then the 7AAD^−^GFP^+^CD45^+^DPP4^−^ and 7AAD^−^GFP^+^CD45^+^DPP4^+^ cells were sorted into separate tubes. A total of 1,000 cells of each phenotype were injected into 10–12-week-old recipient mice. Leukemia burden and DPP4 expression were assessed 10 days post-transplantation.

#### Simian virus 40 immortalized bone marrow stroma

Total bone marrow cells were isolated from the femurs and tibias of B6J.129(B6N)-Gt(ROSA)26Sortm1(CAG-cas9*,-EGFP)Fezh/J mice (Jackson Laboratories). The bones were crushed in PBS (ThermoFisher) with 2% FBS (Gibco) and the released marrow was filtered over a 40 μm strainer. Mononuclear cells were collected by density gradient centrifugation (Ficoll-Paque Plus, Cytiva Life Sciences). A total of 20 × 10^6^ mononuclear cells were put into culture with Alpha-MEM (Gibco) supplemented with 20% FBS (Gibco) and 1% Penicillin–Streptomycin (Gibco) in 150 mm dishes. Non-adherent cells were discarded around day 5 and the media changed every 5–7 days for approximately 3 weeks. At this point, colonies of large adherent fibroblasts were apparent. The cells were detached from the dishes with Trypsin-EDTA (Gibco), counted, and 50,000 cells seeded into two wells of a six-well plate. The following day, one well of cells was transduced with lentivirus in the presence of 8 μg ml^−1^ polybrene (Millipore, Sigma) to deliver the simian virus 40 (SV40) small and large T antigen. The plasmid pLenti CMV/TO SV40 small + Large T (w612-1) was a gift from Eric Campeau (Addgene plasmid 22298; http://n2t.net/addgene:22298; RRID: Addgene_22298). Despite the lack of a selectable marker, the transduced cells divided much more rapidly than the non-transduced primary stroma, and over the course of 2–3 passages, established an SV40 immortalized stromal cell line.

#### Isolation and in vitro stimulation of T cells

Spleens were collected from C57Bl/6J mice and a single-cell suspension was obtained by mechanical dissociation of the tissue over a 70 μm cell strainer (BD Falcon) in RPMI 1640 (ThermoFisher) supplemented with 10% FBS (Gibco) and 1% Penicillin–Streptomycin (Gibco). Red cells were lysed using ACK Lysing Buffer (Quality Biological) followed by removal of non-T-cell splenocytes through magnetic depletion. In brief, the cell suspension was adjusted to 10^8^ cells ml^−1^ and incubated with biotinylated antibodies directed against B220, CD19, Ter119, NK1.1, Cd11b and Gr1 (all from Biolegend, dilution of 1:100, Supplementary Table 6) at a concentration of 5 μg ml^−1^ for 10 min at room temperature on an orbital shaker. This was followed by the addition of 25 μl ml^−1^ streptavidin-conjugated Rapidspheres (Stem Cell Technologies) and an additional 5 min of incubation at room temperature on an orbital shaker. The samples were placed in an EasySep magnet (Stem Cell Technologies) for 5 min and the purified T cells were subsequently decanted into a fresh tube. Purity of the isolated T cells was confirmed to be >95% using FACS analysis. To induce T-cell proliferation, the isolated splenic T cells were plated with the T-cell activator Dynabeads CD3/CD28 (ThermoFisher) in a 1:1 ratio in leukemia cell line medium: RPMI 1640 (Gibco) supplemented with 10% FBS (Gibco), 100 IU ml^−1^ penicillin (Gibco), 100 mg ml^−1^ streptomycin (Gibco) and 5 ng ml^−1^ IL-3 (Peprotech), as well as 100 ng ml^−1^ SCF (Peprotech).

### Flow cytometry

#### Whole bone marrow and micropipette samples

Whole bone marrow and micropipette samples from calvarium and tibia were blocked with anti-mouse Fc Block (BD Biosciences, dilution 1:50) for 10 min at 4 °C. The cells were thereafter stained with blood cell lineage cocktail (Supplementary Table 6) for 30 min at 4 °C. For detection of dead cells 7AAD (BD Biosciences, 0.25 µg) was added to the sample prior to analysis. Flow cytometry was performed on a BD FACS Aria III sorter (BD Biosciences) and all data were analyzed using FlowJo (Treestar).

#### Flow sorting of AML cells for SMARTseq-v4

Prior to sorting, 1 ml PBS (ThermoFisher Scientific) was added to each sample tube, along with 0.1 µg DAPI (ThermoFisher Scientific). The sample was incubated for 10 min, gently vortexed and transferred to the flow cytometer (MoFlo Astrios EQ cell sorter). Single, live, GFP-positive AML cells (see Supplementary Fig. 1 for examples of gating strategy) were sorted into individual wells of a 96-well PCR plate filled with 2.6 µl Lysis Buffer (Takara Bio). Plates were sealed, spun down, snap-frozen and stored at −80 °C prior to preparation for complementary DNA synthesis using the SMARTseq-v4 assay.

#### Flow analysis and sorting of CXCL12^+^ stromal cells for SMARTseq-v4

Prior to sorting or analysis, samples were cell-surface stained with anti-CD45-BV421 (BioLegend, dilution 1:100) for 30 min at 4 °C in Medium-199 (Gibco) supplemented with 2% FBS. Prior to sorting, 1 ml PBS (ThermoFisher Scientific) was added to each sample tube, along with 0.1 µg DAPI (ThermoFisher Scientific). The sample was incubated for 10 min, gently vortexed and transferred to the flow cytometer (MoFlo Astrios EQ cell sorter). Flow cytometry data were analyzed using FlowJo (Treestar). Single, live, CD45^−^DsRed^+^ stromal cells (see Extended Data Fig. 6a for example of gating strategy) were sorted into individual wells of a 96-well PCR plate filled with 2.6 µl Lysis Buffer (Takara Bio). Plates were sealed, spun down, snap-frozen and stored at −80 °C prior to preparation for cDNA synthesis using the SMARTseq-v4 assay.

#### Leukemia burden and DPP4

Leukemic bone marrow was blocked using anti-mouse Fc Block (BD Biosciences, dilution 1:50) for 10 min at 4 °C in Medium-199 supplemented with 2% FBS. Surface staining was thereafter performed with CD45-APC/Cy7 (BD Biosciences, dilution 1:100) and DPP4-PE (BioLegend, dilution 1:20) for 30 min at 4 °C. The cells were then washed and resuspended in Medium-199 supplemented with 2% FBS with 0.25 µg 7AAD (BD Biosciences). Flow cytometry was performed on a BD FACS Aria III sorter (BD Biosciences) and all data were analyzed using FlowJo (Treestar). Extended Data Fig. 8c shows the gating strategy used to distinguish DPP4high, DPP4int and DPP4neg cells. Note that DPP4-positive cells were defined as DPP4high and DPP4int.

#### Leukemia cluster cell surface markers

Leukemic bone marrow was blocked using anti-mouse Fc Block (BD Biosciences, dilution 1:50) for 10 min at 4 °C in Medium-199 supplemented with 2% FBS. Surface staining was thereafter performed with a leukemia cluster cocktail (Supplementary Table 6) for 30 min at 4 °C. The cells were then washed and resuspended in Medium-199 supplemented with 2% FBS with 0.25 µg 7AAD (BD Biosciences). Flow cytometry was performed on a BD FACS Aria III sorter (BD Biosciences) and all data were analyzed using FlowJo (Treestar).

#### Intracellular DPP4 staining

Bone marrow from leukemia-bearing mice was incubated with anti-mouse Fc Block (BD Biosciences, dilution 1:50) for 10 min at 4 °C followed by surface staining with anti-CD45-APC/Cy7 (BD Biosciences, dilution 1:100). Samples were washed and stained with LIVE/DEAD fixable viability dye (ThermoFisher) in accordance with the manufacturer’s instructions. The cells were thereafter fixed with Cytofix/Cytoperm (BD Biosciences) for 20 min at 4 °C. 1× Perm/Wash buffer (BD Biosciences) was then used to wash the cells and the cells were incubated with either anti-DPP4 or an isotype control antibody (Biolegend, dilution 1:20 for both) that were both conjugated to Alexa Fluor 647 in-house (Abcam) for 30 min at room temperature. The cells were then washed one last time in Perm/Wash buffer (BD Biosciences) and resuspended in Medium-199 supplemented with 2% FBS for analysis. Flow cytometry was performed on an LSR II instrument (BD Biosciences) and all data were analyzed using FlowJo (Treestar).

#### Cell cycle analysis

Bone marrow isolated from leukemic mice was blocked with anti-mouse Fc Block (BD Biosciences, dilution 1:50) for 10 min at 4 °C. Surface staining with anti-CD45-APC/Cy7 (dilution 1:100) and DPP4-PE (dilution 1:20) was performed at 4 °C for 30 min. Following this, the samples were washed in Medium-199 supplemented with 2% FBS and then fixed with Cytofix/Cytoperm (BD Biosciences) for 20 min at 4 °C. The fixed cells were thereafter washed with 1× Perm/Wash buffer (BD Biosciences) and resuspended in Perm/Wash buffer containing anti-Ki67-AF647 at a 1:10 dilution for a 30 min incubation. The samples were washed one more time with 1× Perm/Wash buffer (BD Biosciences) and then incubated in 1× Perm/Wash buffer (BD Biosciences) with 2 μg ml^−1^ DAPI (Biolegend) for 10 min. Finally, the samples were spun down to remove the DAPI-containing buffer and resuspended in Medium-199 supplemented with 2% FBS for analysis. Flow cytometry was performed on a BD FACS Aria III sorter (BD Biosciences) and all data were analyzed using FlowJo (Treestar).

#### Analysis of DPP4 expression following co-culture

A total of 250,000 MLL-AF9 or HoxA9-Meis1 leukemia cells were plated at a 1:1 ratio with the following stromal cell lines: NIH-3T3 (American Type Culture Collection, ATCC), MS-5 (RIKEN), MLO-A5 (Kerafast), MC-3T3-E1 (ATCC) and SV40 immortalized bone marrow stroma (Supplementary Information). The cells were grown in RPMI 1640 (Gibco) supplemented with 1% Penicillin–Streptomycin (Gibco), and 10% FBS (Gibco). MLL-AF9 cultures were supplemented with 20 ng ml^−1^ SCF, 10 ng ml^−1^ IL-3 and 10 ng ml^−1^ IL-6 (all cytokines from Peprotech). HoxA9-Meis1 cell cultures were instead grown in 100 ng ml^−1^ SCF and 5 ng ml^−1^ IL-3. The cells were co-cultured for 3 days. For flow cytometry of DPP4, the co-cultures were trypsinized and subsequently blocked with murine Fc Block (dilution 1:50) for 10 min at 4 °C. Surface staining with anti-CD45-APC/Cy7 (dilution 1:100) and DPP4-PE (dilution 1:20) was then carried out for 30 min at 4 °C. The cells were thereafter washed and resuspended in Medium-199 supplemented with 2% FBS with 0.25 µg 7AAD (BD Biosciences) for analysis. Flow cytometry was performed on an LSR II instrument (BD Biosciences) and all data were analyzed using FlowJo (Treestar). For the analysis of DPP4 mean fluorescence intensity, each sample was normalized to a corresponding isotype control antibody-stained sample.

#### Myeloid lineage markers

Bone marrow collected from C57Bl/6J mice was stained with a hematopoietic stem and progenitor cell cocktail (Supplementary Table 6) for 45 min at 4 °C. The cells were then washed and resuspended in Medium-199 supplemented with 2% FBS with 0.25 µg 7AAD (BD Biosciences). Flow cytometry was performed on a BD FACS Aria III sorter (BD Biosciences) and all data were analyzed using FlowJo (Treestar).

### Reverse transcription with quantitative PCR

RT–qPCR was performed to determine levels of *Dpp4* mRNA. A total of 1 × 10^6^ leukemia cells or T cells were lysed and the RNA was extracted using the RNeasy Plus mini kit isolation kit (Qiagen). RNA was subsequently reverse transcribed into cDNA with the SuperScript IV First-Strand Synthesis System (ThermoFisher). The qPCR analysis was performed using iTaq Universal SYBR Green Supermix (Biorad) with primers specific for *Dpp4* (forward, ACCGTGGAAGGTTCTTCTGG; reverse, CACAAAGAGTAGGACTTGACCC) and *Gapdh* (forward, TGTGTCCGTCGTGGATCTGA; reverse, TTGCTGTTGAAGTCGCAGGAG). Threshold values (C_T_) were estimated using CFX Maestro (Biorad) and transcript levels were normalized by subtracting the corresponding *Gapdh* values. The relative amount of RNA is presented as 2^−ΔΔCt^.

### Droplet-based single-cell RNA sequencing

WCBM and Image-seq samples were counted in a hemocytometer and encapsulated for a maximum output of 8,000 cells into emulsion droplets using the Chromium Controller (10x Genomics). scRNA sequencing libraries were subsequently prepared using Chromium Single Cell 3 v2 Reagent kits (10x Genomics). Reverse transcription and library preparations were done on a Biorad T100 Thermo Cycler (Biorad). cDNA libraries and final libraries were quantified on an Agilent BioAnalyzer (Agilent Technologies) using a High Sensitivity DNA kit (Agilent Technologies). Libraries were diluted to 4 nM and pooled before sequencing on the NextSeq 500 Sequencing system (Illumina). Pools were sequenced with 75 cycle run kits (26 bp Read1, 8 bp Index1 and 55 bp Read2) to a saturation level of ~70–80%.

### SMARTseq-v4 library preparation and sequencing

Libraries were prepared using the MANTIS Liquid Handler (Formulatrix) and the Biomek FXP Single Arm System with Span-8 Pipettor (Beckman Coulter). Full-length cDNA was prepared using the SMARTseq-v4 Ultra Low Input RNA Kit for Sequencing (Takara Bio) and sequencing libraries prepared using the Nextera XT DNA library preparation kit (Illumina).

The SMARTseq-v4 assay utilizes the SMART technology switching mechanism at the 5ʹ end of the RNA template to generate full-length cDNA from as little as 10 pg total RNA. The cDNA was assessed for concentration using the Quant-iT Picogreen dsDNA assay kit (Invitrogen, P7589) on the SpectraMax i3 Multi-Mode Detection Platform (Molecular Devices) and normalized to 0.2 ng µl^−1^ prior to library preparation. Full-length cDNA was fragmented using the Nextera technology in which DNA is simultaneously tagged and fragmented. Tagmented samples were enriched and indexed using 18 cycles of amplification with PCR primers, which included dual 8 bp index sequences to allow for multiplexing (Nextera XT Index Kit). Excess PCR reagents were removed through magnetic bead-based cleanup using PCRClean DX beads (Aline Biosciences) on a Biomek FXP Single Arm System with Span-8 Pipettor (Beckman Coulter). The resulting libraries were assessed using a 4200 TapeStation (Agilent Technologies) and quantified using qPCR (Roche Sequencing). Libraries were pooled and sequenced on a NextSeq Mid Output flow cell using paired, 75 bp reads (Illumina).

### 10x scRNA-Seq data processing

For the 10x scRNA-Seq data, fastq files were obtained using bcl2fastq (v1.8.4). Reads were aligned to the mm10 mouse reference genome using the Cellranger pipeline (v3.0.2, 10x Genomics) with default parameters. The obtained read count matrices were further filtered based on two quality metrics: the number of total UMI counts per cell (>800); and the mitochondrial transcript ratio per cell (<0.2). We used Conos (v1.4.1, https://github.com/kharchenkolab/conos)^21^ to integrate multiple scRNA-seq datasets. Each individual dataset was first normalized using the basicP2proc function in pagoda2 (v1.0.10) using default parameters (https://github.com/kharchenkolab/pagoda2/releases/tag/v1.0.10). Different samples were then aligned using Conos with default parameter settings (PCA space with 30 components, angular distance, mNN matching, k = 15, k.self=5), and UMAP embedding was estimated using default parameter settings. Leiden clustering (conos::findCommunities) was used to determine joint cell clusters across the entire dataset collection. Quality parameters for the 10x scRNA-seq data are listed in Supplementary Table 7 and Extended Data Fig. 5.

#### Differential expression

For differential expression analysis between cell types in the 10x scRNA-seq data, a Wilcoxon rank sum test, implemented by the getDifferentialGenes() function from Conos R, was used to identify statistically significant marker genes that were expressed in each cell cluster (Supplementary Table 1). The genes were considered differentially expressed if the *P* value-determined Z score was greater than 3. For differential expression analysis between Image-seq and WCBM (for example, Image-seq monocytes versus WCBM monocytes), the getPerCellTypeDE() function in Conos was utilized.

#### Cell annotation

Annotation of the cluster communities was done using marker gene expression. Initial annotations were obtained by entering the top 100 differentially expressed genes in each cluster (ordered by logFoldChange) into the CellKb database^65^ and further refined by consulting the primary literature referenced therein along with other relevant publications. Specificity of selected markers was additionally confirmed by evaluating expression in the Haemopedia^66^ and CellMarker^67^ bone marrow datasets. We identified markers to classify ten major cell types: B cell (*Ms4a1*, *Ly6d*, *Cd79a*), pre/pro-B cell (*Vpreb1*, *Vpreb3*, *Dntt*), basophil (*Mcpt8*, *Prss34*, *Ms4a2*), dendritic cell (*Bst2*, *Irf8*, *Siglech*, *Cox6a2*), erythroblast (*Car2*, *Hemgn*, *Ctse*, *Cpox*, *Atpif*), monocyte (*Lyz2*, *Ctss*, *s100a4*), monocyte progenitor (*Ly6c2*, *S100a10*), diverse progenitors (*Cd34*, *Prtn3*, *Mpo*, *Elane*, *Mpl*), granulocyte progenitor (*S100a8*, *S100a9*, *Cebpe*, *Fcnb*) and neutrophil (*Mmp8*, *Ifitm6*, *S100a11*, *S100a8*, *S100a9*).

### SMARTseq-v4 sequencing data analysis

SMARTseq-v4 sequencing data were aligned with hisat2 (ref. ^68^) (v4.8.2), using the genome reference mm10. We used featureCounts^69^ (v1.6.4) to calculate read counts. The quality of cells was then assessed based on the number of total counts per cell (library size), requiring at least 500,000 reads per cell. A total of 84 AML and 43 stroma cells were retained for downstream analysis. Seurat was used to analyze the SMARTseq-v4 data, and the quality parameters are listed in Supplementary Table 7 and Extended Data Fig. 5d,e. In brief, we first performed data normalization using the NormalizeData function with default settings. The top 30 principal components were used to construct shared nearest neighbors graphs and UMAP embeddings. The FindAllMarkers function from Seurat was used to identify differentially expressed (marker) genes for clusters or subtypes. Mesenchymal stromal and endothelial cells were annotated using well-established marker genes: for mesenchymal stromal cells the marker genes were Cxcl12, Pcolce, Ogn and Adipoq; and for the endothelial cells the markers were Eng, Kdr, Plvap and Egfl7 (see also the heatmap in Extended Data Fig. 6d, as well as ref. ^27^).

#### Cell cycle signature score

To assess cell states in different cell subsets and conditions we used a gene set signature score to measure the relative difference of cell cycle states. The signature scores were calculated as average expression values of the genes in a given set. The signature gene list was downloaded from Whitfield et al.^70^. Hierarchical clustering of cell cycle signature score was used to group AML cells. A two-sided Student’s *t*-test was used to assess differential expression of selected cell cycle genes between proliferating (P) and non-proliferating (NP) cells (Supplementary Table 4).

#### Regressing out cell cycle genes

Seurat^71^ (v4.0.6) was used to regress out cell cycle genes. First, we assigned each cell a score, based on its expression of G2/M and S phase markers with the CellCycleScoring function. Then we applied the ScaleData function to regress out the cell cycle genes. The scaled residuals of this model represent a ‘corrected’ expression matrix that can be used downstream for dimensionality reduction. UMAP embedding and graph-based shared nearest neighbor clustering were used with five principal components, and Seurat::FindClusters() was used to identify AML cell sub-clusters. Seurat::FindAllMarkers, which utilizes a two-sided Wilcoxon rank sum test to assess statistical significance, was used to find the differentially expressed genes within each sub-cluster (Supplementary Table 2).

#### Analysis of differentially expressed genes

DESeq2 (ref. ^60^) was used to analyze differentially expressed genes between P and NP cells (Fig. 5a and Supplementary Table 3), as well as between P + IM (that is, intermediate) and NP cells (Supplementary Table 5).

#### GO term enrichment

To test for enriched GO biological processes in gene sets, the ClusterProfiler^72^ (v4.0.0) package was used to evaluate the enrichment of GO categories in the sets of upregulated and downregulated genes and rank them by adjusted *P* value (Fig. 5h and Extended Data Figs. 5d,e and 8g). The set of all expressed genes was used as a background.

### Analysis of human AML data

A regular gene expression correlation analysis was applied to two published, bulk RNA-seq datasets (531 patients for the Oregon Health and Science University, OHSU dataset^48^, and 188 patients for The Cancer Genome Atlas, Firehose dataset^49^) collected from https://www.cbioportal.org/. Spearman correlation coefficients for each gene with DPP4 were calculated. The top 300 positively correlated genes (based on strength of the correlation coefficient) were determined for each of the two datasets (Firehose and OHSU). Interestingly, we observed a high degree of overlap between the top 300 positively correlated genes from the two datasets. The overlapping gene set was then used for GO analysis.

### Multiphoton imaging experiments

#### Image acquisition

To image the AML cell distribution in different cavities, as well as CellTracker CMTPX retention, two-photon excitation at 900 nm was used, and the emission was collected at 340–460 nm to detect the second-harmonic generation signal of collagen (bone), while 500–550 nm was used to detect the AML-GFP signal. The bone front staining and CMTPX were excited at 775 nm and the resulting fluorescence collected using 525/50 nm (tetracycline) and 617/73 nm (Alizarin Red, CMTPX) filters. All image stacks were acquired using a previously described microscope^51,61^, with a 2 μm step size from the calvaria surface, and 20 frames from the live scanning microscope (30 frames per second) were averaged to acquire a single image.

For imaging the stroma, β-actin-GFP mice, DPP4 expression in vivo, as well as for quantifying AML proliferation, the home-built microscope described above was used, with an imaging wavelength of 980 nm. Emission signals were collected with filter 439/154 for the second-harmonic generation signal, filter 525/50 for GFP and filter 607/70 for the AF568 signal with a combination of FF705 LP, FF495 LP and FF552LP dichroic mirrors. For imaging the CXCL12-DsRed stroma, two-photon excitation at 980 nm was used, and emission signals were collected using the filter 439/154 for the second-harmonic generation signal (blue channel, shown as gray in the Figures) and 585/40 for DsRed (red channel) with the same dichroic mirrors as listed above. Image stacks were acquired with a step size of 2 µm, as well as a 15-frame average.

For displaying the data, some images were background-subtracted with the mode of the stack histogram (corresponding to the noise pixel intensity) and subsequently filtered using the 3D Fast Filters (median) function in FIJI with an *x*, *y* and *z* radius unit of 1. The brightness and contrast of images in the figures were adjusted, but in all cases the image analysis was performed using the raw data.

#### Image stitching and maximum intensity projections

Large area images were obtained by stitching together images from individual microscope fields of view sequentially for each *z* plane using the Grid/Collection stitching plugin in Fiji and using an overlap of 30%. Maximum intensity projection images were obtained using the Z Project function (Fiji).

#### Characterization of P, NP and IM cells

The same bone marrow cavities in the same animals were imaged both on day 1 and day 3 after transplantation of AML cells. Identical cavities on day 3 were found using the recorded coordinates with respect to the bregma and lambda reference points, and comparison of the signal distribution and specific landmarks in the second-harmonic generation channel. The number of AML cells was quantified at both timepoints, and the fold-change in AML cells between day 1 and day 3 was calculated for each cavity:Fold-change = (no. of cells_day 3_ − no. of cells_day 1_)/(no. of cells_day 1_).

Cells were then grouped based on their fold-change as either proliferating (P, fold-change >2), intermediate (IM, fold-change >0 and ≤2), or non-proliferating (NP, fold-change ≤0), which corresponded to average cell numbers of 29.6 (with a 99.9% confidence interval of 21.7 to 37.4 cells), 13.2 (with a 99.9% confidence interval of 5.5 to 20.8 cells), and 2.1 (with a 99.9% confidence interval of −1.2 to 5.4 cells), respectively, on day 3.

#### Analysis of cell tracker labeling

AML cells were labeled with CellTracker Red (CMTPX, 10 μM, ThermoFisher Scientific) before transplantation. In brief, the cell suspension (in Ca^2+^/Mg^2+^ free PBS containing 10 μM CMTPX) was incubated at 37 °C for 45 min. Cells were then pelleted to remove the staining solution and resuspended in 300 μl PBS for retro-orbital injection. The mean CellTracker intensity was measured on day 3 after transplantation and measured at the brightest plane of the cell. The cells were considered positive for CMTPX when the measured signal was greater than 12.5 (the background noise in the marrow cavity). The number of CMTPX-positive cells was then divided by the total cell number sampled from the bone marrow cavities harboring the same cell counts (*n* = 7 mice).

### Analysis of DPP4 expression in vivo

DPP4 antibody (BioLegend) and Isotype control antibody (BioLegend) were conjugated to AF568 using the Lightning Link kit (Abcam). Antibody and isotype were injected retro-orbitally 1 h prior to the imaging session at a dosage of 1 mg kg^−1^. Images were collected with an excitation wavelength of 980 nm (1.6 nJ pulse energy, filter set detailed above), using a z-step of 2 µm with a 15-frame average on the custom-built microscope described above.

AML cells were segmented based on the GFP signal in the obtained images. For this, seeds were generated using the interactive watershed tool (Fiji) and used as input for the 3D-Watershed segmentation plugin (Fiji). The images were thresholded to generate a mask. This was used to calculate the total AF568 signal, as well as the total GFP signal, in each cell using the red and green channels of the images, respectively, as input for the 3D Object counter plugin (Fiji). The ratio of red to green fluorescence (multiplied by 10) for each cell was plotted in Fig. 5c both for the DPP4 and isotype control cells.

#### Distribution of AML cells in D-, M- and R-type cavities

The protocols to determine the bone remodeling status have been described previously^51^. The first calcium-chelating reagent dye 1 (Tetracycline, Sigma, 35 mg kg^−1^) was given i.p. 48 h prior to imaging to label the bone fronts and track the bone resorption activities over the course of 2 days. The second calcium-chelating reagent dye 2 (Alizarin Red, 40 mg kg^−1^) was injected on the day of imaging to label all of the bone fronts. The bone remodeling status was then defined based on the ratio of dye 1 to dye 2 in a single bone marrow cavity (the concave endosteum), and therefore the bone marrow cavities were classified as: deposition type (D-type; dye 1 : dye 2 ratio > 75%); resorption type (R-type; dye 1 : dye 2 ratio < 25%), or mixed type (M-type; dye 1 : dye 2 ratio 25–75%. The distributions of seeding and expansion of AML cells were then mapped with respect to the D-, M- and R- type cavities on day 0 (3 h after transplantation), day 1 and day 3 after transplantation. The same mouse was followed up longitudinally on day 0 and day 1 and the cavity type was defined on day 0. A separate cohort of animals was used for day 3 to avoid unwanted inflammatory responses from the survival surgical procedures.

### Animal handling

Male and female 8-week-old C57Bl6/J mice (cat. no. 000664) or male and female CXCL12-DsRed mice (cat. no. 022458) were ordered from the Jackson Laboratory, housed in our animal facility for at least 2 weeks and used for experiments at between 10 and 14 weeks of age. The 8-week-old female Fezh/J mice B6J.129(Cg)-Gt(ROSA)26Sortm1.1(CAG-cas9*,-EGFP)Fezh/J were ordered from the Jackson Laboratory (cat. no. 026179) and used in experiments. Male and female β-actin-GFP mice (Jackson Laboratory, cat. no. 006567) were bred in-house and used between 10 and 16 weeks of age. Male and female β-actin-DsRed mice (Jackson Laboratory, cat. no. 006051) were bred in-house and used between 10 and 16 weeks of age. The β-actin luciferase (βact) mice from Taconic (cat. no. 11977) were bred with ubiquitin-c-GFP (UcGFP) mice from the Jackson Laboratory (cat. no. 004353) to generate βact-UcGFP transgenic mice. An 8-week-old female βact-UcGFP mouse was then used to generate the HA9M1 cell line. All mice were housed in the pathogen-free Massachusetts General Hospital (MGH) Animal Facilities, which were equipped with ventilated micro-isolator cages. Sentinel programs and veterinary oversight were in place. Mice were given standard chow and drinking water ad libitum. An automated 12 h dark–12 h light cycle was observed and mice were housed at a fixed temperature (21 °C) and humidity (66%). The MGH Animal Facility is under the supervision of the MGH Center for Comparative Medicine. All facilities are fully accredited by the Association for Assessment and Accreditation of Laboratory Animal Care International (000809) and meet the National Institutes of Health standards as set forth in the *Guide for Care and Use of Laboratory Animals* by the Department of Health and Human Services. All procedures involving animals were carried out in agreement with protocols 2012N000190, 2007N000148 or 2016N000085 approved by the Institutional Animal Care and Use Committee of Massachusetts General Hospital.

### Statistics and reproducibility

*P* < 0.05 was considered significant unless specified otherwise. The number of biological replicates and independent experiments for each graph, along with the test statistic, are listed in each Figure and Extended Data Figure and legend. A list of statistical parameters for these, including confidence intervals, degrees of freedom, mean, standard deviation and effect sizes can be found in the Supplementary Information. The number of biological replicates and independent mice for all images that are shown in the Figures and Extended Data Figures are also included in the respective Figure legends.

### Reagents

A list of antibodies is given in Supplementary Table 6 and a list of reagents is given in Supplementary Table 8.

### Reporting summary

Further information on research design is available in the Nature Research Reporting Summary linked to this article.

## Online content

Any methods, additional references, Nature Research reporting summaries, source data, extended data, supplementary information, acknowledgements, peer review information; details of author contributions and competing interests; and statements of data and code availability are available at 10.1038/s41592-022-01673-2.

### Supplementary information

Supplementary Information Supplementary statistics, Supplementary Fig. 1 and Supplementary Image-seq protocol.

Reporting Summary

Supplementary Tables Table 1: Differentially expressed genes within each cluster from Fig. 3b, ordered by z-score. Table 2: Differentially expressed genes within each cluster from Fig. 4h, sorted by *P* value. Table 3: Differentially expressed genes between P and NP cells, sorted by log_2_FoldChange. Table 4: A list of cell cycle-related genes that are differentially expressed between P and NP cells. Table 5: Differentially expressed genes between P + IM and NP cells, sorted by log_2_FoldChange. Table 6: A list of all antibodies used in the experiments described in the manuscript. Table 7: Quality parameters for all Sequencing Data (SMARTseq-v4 and 10x). Table 8: A list of reagents used for the experiments described in the manuscript.

Supplementary Video 1 **In vivo cell aspiration video**. Confocal reflectance signal (red) is used to visualize the micropipette, bone marrow, vasculature and bone. Two-photon microscopy is used to visualize the target leukemia cell (cyan). We generated an opening to the bone marrow with plasma-mediated ablation (see the text for details) prior to recording the video. The video shows insertion of the micropipette through this opening and into the bone marrow, positioning of the micropipette next to the target cell, and aspiration of this cell in the micropipette. The micropipette is then removed for processing of the sample. The outline of the bone marrow cavity is highlighted in white, and an arrow points to a blood vessel where we can see active blood flow, demonstrating that the procedure is being carried out in a live animal.

Supplementary Video 2 **Stromal cell aspiration video**. Multiphoton microscopy is used to visualize the bone (second-harmonic generation, gray) and CXCL12-DsRed cells (red). Prior to recording the video, the micropipette (red, outlined in white) has been inserted through the opening in the bone and positioned next to the target cells. The video shows aspiration of the target stromal cells into the micropipette by suction with an air syringe.

Supplementary Video 3 **Video of cell aspiration in the tibia**. Multiphoton microscopy is used to visualize the bone (second-harmonic generation, gray) and β-actin-GFP^+^ bone marrow cells (green). Prior to recording the video, the micropipette (red) has been inserted through the opening in the bone and positioned next to the target cells. The video shows aspiration of the target bone marrow cells into the micropipette by suction with an air syringe.

## Data Availability

The 10x-seq and SMARTseq-v4 data generated in this work have been deposited in the Gene Expression Omnibus (GEO) database GSE188902 (https://www.ncbi.nlm.nih.gov/geo/query/acc.cgi?acc=GSE188902), which is publicly available. The mouse mm10 reference genome was downloaded from 10x Genomics (https://support.10xgenomics.com/single-cell-gene-expression/software/downloads/latest?). Public, bulk RNA-seq AML datasets from OHSU (https://cbioportal-datahub.s3.amazonaws.com/aml_ohsu_2018.tar.gz) and TCGA (https://cbioportal-datahub.s3.amazonaws.com/laml_tcga_pan_can_atlas_2018.tar.gz) were download from cbioportal. Due to the extremely large file sizes accompanying the extensive imaging data, raw image data are available from the corresponding authors upon request. Cell lines are available from the authors upon request and mouse lines are commercially available at the Jackson Laboratory. Source data files for all graphs in the Figures and Extended Data Figures are linked to the online version of the manuscript. Source data are provided with this paper.

## Source data

Source Data Fig. 2 2b: Viability data for Fig. 2b. 2d: Averaged cell proportions from flow cytometry for individual bone marrow (BM) hematopoietic populations listed as percentage of live cells.
Source Data Fig. 3 3d: Coordinates for each cell from UMAP embeddings shown in Fig. 3b (Image-seq) and 3c (WCBM). 3d: Correlation between Image-seq and WCBM data. 3e: Expression of marker genes within the annotated cell clusters. 3f: Number of expressed genes and total UMI in each cell from Image-seq and WCBM samples. 3g: GFP expression in each cell from high-burden and low-burden samples. 3h: Coordinates for each cell from UMAP embeddings of high-burden and low-burden samples using the annotations derived in Fig. 3b,c.
Source Data Fig. 4 4b: The number of HA9M1 cells detected per cavity on day 0, 1 and 3 after transplantation of 3 million cells. 4c: Number of cells per cavity on day 3 after transplantation of 3 million cells. 4f: Number of P, NP and IM cells that were classified as being in the (S, G1/S), (G0) and (G2, G2/M) phases of the cell cycle. 4g: Distribution of P, NP and IM cells between AML-GMP, AML-AP1 and AML-mono clusters. 4h: Coordinates for each cell from the UMAP embedding of AML SMARTseq cells. 4i: Expression levels of genes in each AML cell that are shown in the heatmap from Fig. 4i.
Source Data Fig. 5 5a: Differentially expressed genes between P and NP cells, ordered by adjusted *P* value. 5b: Percentage of leukemia cells that were DPP4-positive as a function of disease progression (time). 5c: Ratio of red (that is, DPP4-AF568) to green (that is, AML-GFP) fluorescence (multiplied by 10) is listed for P and NP cells that were imaged after injection of AF568 antibody, as well as control cells labeled with isotype-AF568 antibody. 5e: Percentage of DPP4-positive and DPP4-negative AML cells in different phases of the cell cycle as analyzed by Ki-67 staining and flow cytometry in the HA9M1 leukemia model at day 7 after transplantation. 5f: Percentage of DPP4-positive and DPP4-negative AML cells in different phases of the cell cycle as analyzed by Ki-67 staining and flow cytometry in the MLL-AF9 leukemia model at day 7 after transplantation. 5g: DPP4-correlated genes from OHSU and Firehose datasets. 5h: Top GO terms for overlapping genes from Fig. 5g, along with the associated *P* values.
Source Data Fig. 6 6a: DPP4 mean fluorescence intensity ratio (sample/isotype) analyzed by cell surface staining and flow cytometry of HA9M1 and MLL-AF9 cells after 3 day co-culture with stromal cells. 6b: The number of HA9M1 cells detected per D, M and R cavity on day 0, 1 and 3 after transplantation of 3 million cells.
Source Data Extended Data Fig. 3 Cell proportions (percentage of live cells) for each hematopoietic cell population from flow cytometry analysis of individual micropipette and whole bone marrow samples used to derive the statistics in Extended Data Fig. 3b.
Source Data Extended Data Fig. 4 4a: Proportions of the major hematopoietic populations averaged over all Image-seq and WCBM samples. 4b,c: Proportions of the major hematopoietic populations in each Image-seq and WCBM sample. 4d: Top GO terms obtained from upregulated genes in Image-seq samples. 4e: Top GO terms obtained from downregulated genes in Image-seq samples.
Source Data Extended Data Fig. 5 5a: Percentage of cells that passed quality control in each 10x run. 5b: Number of expressed genes and total UMI in each cell from each Image-seq and WCBM run. 5d: The number of expressed genes and total mapped reads in each cell from our WCBM (10x), Image-seq (10x) and Image-seq SMART-seqv4 AML data. 5e: Number of expressed genes and the total number of mapped reads (that is, molecules) in each AML cell processed with the Smartseq-v4 protocol.
Source Data Extended Data Fig. 6 6a: Coordinates for each cell from UMAP embedding of stroma cells including cell annotation. 6c: Expression levels of genes in each stromal cell that are shown in the heatmap from Extended Data Fig. 4c. 6d: Expression levels of literature marker genes (DOI: 10.1016/j.cell.2019.04.040) in each stromal cell.
Source Data Extended Data Fig. 7 7a: Percentage of cell tracker+ cells as a function of the number of cells per cavity. 7b: Expression levels of selected cell cycle-relevant genes from Supplementary Table 7. 7c: Scaled average expression of gene sets in the G2, G2/M, G1/S and S phase of the cell cycle in each AML cell.
Source Data Extended Data Fig. 8 8a: Percentage of DPP4-positive and DPP4-negative AML cells in different phases of the cell cycle as analyzed by Ki-67 staining and flow cytometry in the HA9M1 leukemia model at day 13 after transplantation. 8d: Percentage of DPP4high-int-neg cells isolated from recipients transplanted with 1,000 DPP4high or DPP4neg HA9M1 cells and analyzed on day 10 after transplantation. 8e: Mean fluorescence intensity of Itgb7, Flt3 and CD48 detected in DPP4high-neg HA9M1 cells by cell surface staining and flow cytometry 3 weeks after transplantation. 8f: DPP4, Itgb7, Flt3 and CD48 expression levels for each cell in the three AML clusters from Fig. 4h. 8g: Top GO terms associated with differentially expressed genes between P and NP cells.
Source Data Extended Data Fig. 9 9a: Percentage of DPP4-positive cells in various myeloid cell populations.
Source Data Extended Data Fig. 10 10b: Analysis of DPP4 expression (mRNA) by qPCR on in vitro cultured HA9M1 and MLL-AF9 cells. 10d: Analysis of DPP4 expression (mRNA) by qPCR on AML cells collected from the bone marrow 3 weeks after transplantation.

## Extended data

are available for this paper at 10.1038/s41592-022-01673-2.

## Code availability

The codes generated during this study are available at the GitHub repository (https://github.com/shenglinmei/Image-seq) and Zenodo (https://zenodo.org/record/7067661).
