# Supplementary material for: Image-seq: spatially resolved single-cell sequencing guided by in situ and in vivo imaging
Source: Nat Methods. 2022 Nov 24;19(12):1622–33. doi: 10.1038/s41592-022-01673-2 (PMC9718684; doi:10.1038/s41592-022-01673-2)
Supplement: Supplementary file 1 — Supplementary statistics, Supplementary Fig. 1 and Supplementary Image-seq protocol. [file 41592_2022_1673_MOESM1_ESM.pdf]

nature methods

Article

https://doi.org/10.1038/s41592-022-01673-2

# Image-seq: spatially resolved single-cell sequencing guided by in situ and in vivo imaging

In the format provided by the authors and unedited

# Table of Contents

**<u>Included in this pdf:</u>**


1) **Supplementary Statistics**
   A list of statistical parameters for graphs from the main manuscript Figures and Extended Data Figures, including confidence intervals, degrees of freedom, mean, standard deviation and effect sizes.
2) **Supplementary Figure 1**
   Gating strategy used for sorting AML cells into individual wells.
3) **Supplementary Image-seq protocol.**
   Detailed protocol describing the steps involved in the Image-seq procedure.


**<u>Available online</u>:**


4) **Supplementary Video 1**
   In vivo cell aspiration video.
5) **Supplementary Video 2**
   Stromal cell aspiration video.
6) **Supplementary Video 3**
   Video of cell aspiration in the tibia.
7) **Supplementary Table S1**
   Differentially expressed genes within each cluster from Figure 3b, ordered by z-score.
8) **Supplementary Table S2**
   Differentially expressed genes within each cluster from Figure 4h, sorted by p value.
9) **Supplementary Table S3**
   Differentially expressed genes between P and NP cells, sorted by log2FoldChange.
10) **Supplementary Table S4**
   A list of cell-cycle related genes that are differentially expressed between P and NP cells.
11) **Supplementary Table S5**
   Differentially expressed genes between P+IM and NP cells, sorted by log2FoldChange.
12) **Supplementary Table S6**
   A list of all antibodies used for the experiments described in the manuscript.
13) **Supplementary Table S7**
   Quality parameters for all Sequencing Data (SMARTseq-v4 and 10x).
14) **Supplementary Table S8**
   A list of reagents used for the experiments described within the manuscript.

## 1) Supplementary Statistics

Confidence intervals (CI), degrees of freedom (DOF), mean, standard deviation (SD) and effect sizes (ES) for graphs from the main manuscript Figures and Extended Data Figures (wherever applicable) are listed below.

**Figure 3: f)** LEFT: ES 0.17, 95%-CI 0.16 to 0.18. RIGHT: ES 97.5, 95%-CI 83 to 112.

**Figure 4: b)** Day 0: mean 2.80 SD 2.08, Day 1: mean 2.82 SD 1.89, Day 3: mean 7.06 SD 6.53. **c)** *IM vs NP*: ES 11.1 95%-CI 5.2 to 16.9, DOF 36.6. *P vs IM*: ES 16.4 95%-CI 8.4 to 24.4 DOF 106.5.

**Figure 5: b)** Day 3: mean 0.00 SD: 0.00; Day 4: mean 0.00, SD: 0.00; Day 5: mean 15.20, SD: 7.14; Day 7: mean 6.93, SD: 2.22; Day 9: mean 11.75, SD 1.69; Day 11: 15.45, SD 4.58; Day 13: mean 26.60, SD: 6.20; 2 weeks: 41.00, SD: 7.31; 4 weeks: mean 46.97, SD: 18.92. **c)** *P vs NP*: ES 0.24 U 909. **e)** DOF: 14. *G1*: ES -14.0 95%-CI -17.2 to -10.8; *S/G2/M*: ES 13.64, 95%-CI:11.22 to 16.06. **f)** DOF: 4.00. *G0* ES: 0.38 95%-CI 0.11 to 0.66; *G1*: ES -60.27, 95%-CI -65.76 to -54.77; *S/G2/M*: ES 52.47, 95%-CI 43.75 to 61.18.

**Figure 6: a)** DOF 24. **LEFT**: *BM stroma*: ES -0.78, 95%-CI -1.46 to -0.10; *MC3T3*: ES-1.46, 95%-CI -2.29 to -0.62. **RIGHT**: *BM stroma*: ES -0.68, 95%-CI -1.14 to -0.22; *MC3T3*: ES -1.22, 95%-CI -1.79 to -0.66. **b)** *D3 vs D1*: ES 2.0, U 51; *M3 vs M0*: ES 4.0 U 1897; *M3 vs M1*: ES 3.0 U 1695; *R3 vs R0*: ES 4.0 U 219; *R3 vs R1*: ES 4.0 U 110.

**Extended Data Figure 3: b)** Two-way ANOVA. *Macrophage M vs W*: ES -0.87, 95%-CI -1.25 to -0.48; *B-cell C vs T*: ES -3.38, 95%-CI -5.38 to -1.41; *Pre-B-cell M vs W*: ES -5.00, 95%-CI -7.22 to -2.77; *Pro-B-cell M vs W*: ES -0.38, 95%-CI -0.48 to -0.27; *Pre-pro-B-cell C vs T*: ES -2.48, 95%-CI -3.54 to -1.41.

**Extended Data Figure 5: d) LEFT**: *SMARTseq vs WCBM:* ES 3.02 95%-CI 2.97 to 3.07, *SMARTseq vs 10xImage-seq* ES 2.86 95%-CI 2.80 to 2.91. **RIGHT**: *SMARTseq vs WCBM*: ES 7179.5 95%-CI 6947 to 7412, *SMARTseq vs 10xImage-seq:* ES 7049.5 95%-CI 6815 to 7284. **e) LEFT**: mean 8053, SD 1519. **RIGHT**: mean 6.35, SD 0.13.

**Extended Data Figure 7: b)** *Prdx1*: DOF 63, ES -0.22 95%-CI -0.37 to -0.07. *Arpp19*: DOF 52, ES -0.16 95%-CI -0.31 to 0. *Cdc123*: DOF 56, ES -0.19 95%-CI -0.35 to -0.03. *Cdk20*: DOF 33, ES -0.085 95%-CI -0.15 to -0.023. *Eps8*: DOF 35, ES -0.090 95%-CI -0.15 to -0.025.

**Extended Data Figure 8: a)** DOF: 12, *S/G2/M*: ES 2.91, 95%-CI: 0.29 to 5.54. **d) LEFT**: *DPP4*$^{neg}$: mean 0.045, SD 0.039; *DPP4*$^{high}$: mean 0.048, SD 0.023. **RIGHT**: DOF: 5, *DPP4*$^{neg}$: ES -19.70, 95%-CI -37.61 to -1.79. **e)** DOF: 12. *Itgb7 DPP4*$^{neg}$ *vs DPP4*$^{high}$: ES -1595, 95%-CI -1826 to -1365; *Itgb7 DPP4*$^{int}$ *vs DPP4*$^{high}$: ES -1570, 95%-CI -1800 to -1339; *Flt3 DPP4*$^{neg}$ *vs DPP4*$^{high}$: ES -168.4, 95%-CI -224.1 to -112.7; *Flt3 DPP4*$^{int}$ *vs DPP4*$^{high}$: ES -167.8, 95%-CI -223.5 to -112.1; *CD48 DPP4*$^{neg}$ *vs DPP4*$^{high}$: ES -3470, 95%-CI -4524 to -2417; *CD48 DPP4*$^{int}$ *vs DPP4*$^{high}$: ES -3390, 95%-CI -4444 to -2337. **f)** *Dpp4 AML-mono vs AML-GMP*: ES 2.13e-05 95%-CI 4.76e-05 to -4.99e-06, *DPP4 AML-mono vs AML-AP1*: ES 1.89e-05 95%-CI 3.78e-05 to -7.39e-05. *Itgb7 AML-mono vs AML-GMP*: ES 0.46 95%-CI 0.79 to 0.12. *Itgb7 AML-mono vs AML-AP1:* ES 0.33 95%-CI 0.65 to 4.30e-05. *Flt3 AML-mono vs AML-GMP*: ES 0.63 95%-CI 0.87 to 0.38, *Flt3 AML-mono vs AML-AP1*: ES 0.56 95%-CI 0.83 to 0.28. *Cd48 AML-mono vs AML-GMP*: ES 0.30 95%-CI 0.49 to 0.11, *CD 48 AML-mono vs AML-AP1*: ES 0.36 95%-CI 0.59 to 0.12.

**Extended Data Figure 9: a)** *LKS* mean 41.27, SD 2.67; *MEP* mean 12.09, SD 3.03; *CMP* mean 6.75 SD 1.26; *StemGMP* mean 7.01, SD 1.57; *MonoGMP* mean 2.37, SD 0.50; *GrGMP* mean 0.43, SD 0.25; *Granulocytes* mean 0.081, SD 0.021; *Inflammatory monocytes* mean 1.56, SD 0.69; *Patrolling monocytes* 4.08, SD 0.56; *Macrophages* mean 25.53, SD 2.33; *Lymphoid cells* mean 68.68, SD 2.48.

**Extended Data Figure 10: b)** *Spleen*: mean 1, SD 0; *MLL-AF9*: mean 0.0015, SD: 0.00022; *HA9M1*: mean 0.00, SD 0.00. **d)** DOF: 5. *T cells day 0 vs day 4*: ES 0.025, 95%-CI -0.194 to 0.143; *day 0 DPP4+ vs DPP4-*: ES 0.69, 95%-CI 0.524 to 0.860; *DPP4+ day 0 vs day 4*: ES 0.68, 95%-CI 0.515 to 0.852; *day 4 DPP4+ vs DPP4-*: ES 0.012, 95%-CI -0.157 to 0.180.

**Figure S1: Gating strategy used for sorting AML cells into individual wells.** a) Examples of GFP gating strategy used to isolate proliferating and non-proliferating HA9M1 cells for cell lysis and SMARTseq-v4 library preparation. b) Gating strategy for exclusion of DAPI-labeled (dead) cells.

**a** **Non-proliferating** **Proliferating**

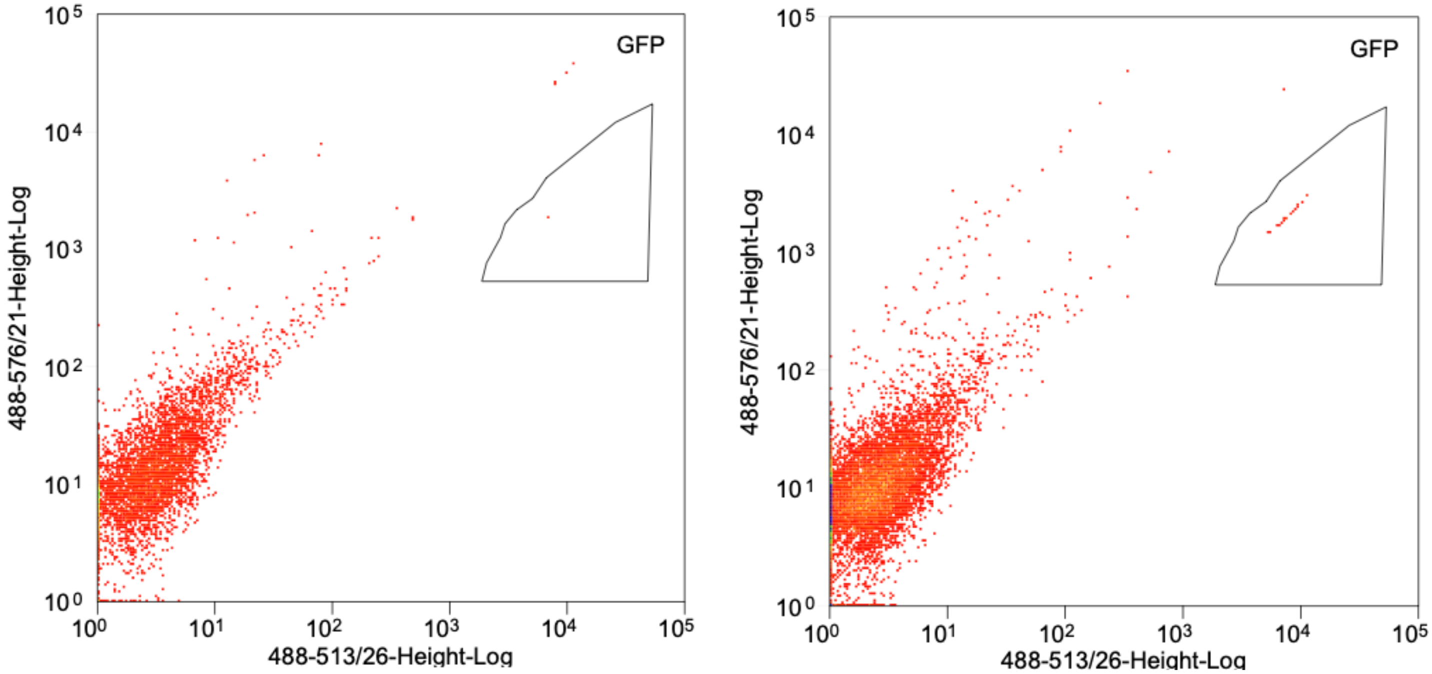


**b** **DAPI**

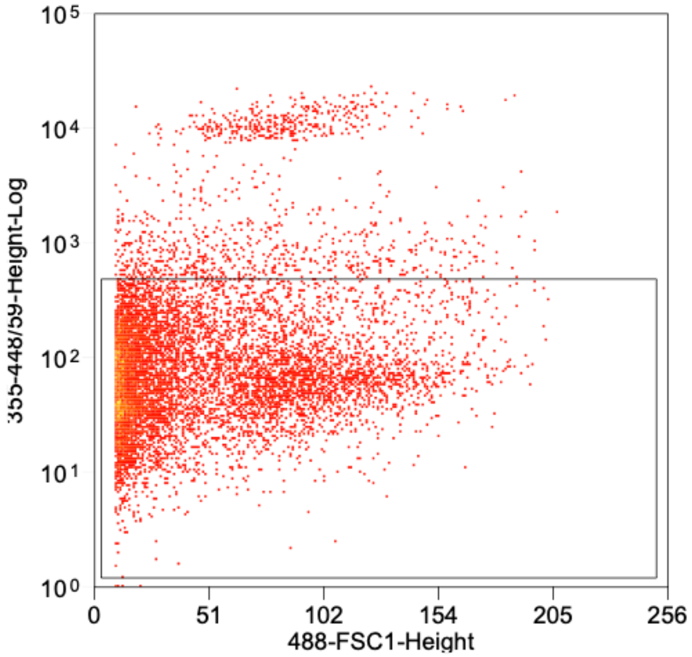

# Detailed protocol for Image-seq: spatially-resolved single cell sequencing guided by in situ and in vivo imaging


## Abstract

Tissue function depends on proper cellular organization. While the properties of individual cells are increasingly being deciphered using powerful single-cell sequencing technologies, understanding their spatial organization and temporal evolution remain a major challenge. Here, we present a detailed protocol for Image-seq, a technology that provides single-cell transcriptional data on cells that are isolated from specific spatial locations under image guidance, thus preserving the spatial information of the target cells. It is compatible with in situ and in vivo imaging and can document the temporal and dynamic history of the cells being analyzed. Cell samples are isolated from intact tissue and processed with state-of-the-art library preparation protocols. The technique therefore combines spatial information with highly sensitive RNA sequencing readouts from individual, intact cells. We describe how the technique can be combined with high-throughput, droplet-based sequencing using 10x genomics, as well as SMARTseq-v4 library preparation for in-depth sequencing of rare cells. Furthermore, Image-seq's ability to isolate viable, intact cells should make it compatible with a range of downstream single-cell analysis tools including multi-omics protocols.


## Introduction

Spatial transcriptomics is a rapidly advancing field, encompassing a range of different technologies, capable of spatially-resolved gene expression analysis [1–9]. In contrast to single-cell RNA sequencing, which provides deep insights into heterogeneities within cell populations, but does not preserve the spatial relationship among individual cells, techniques such as MERFISH[9], seqFISH[4], and Slide-seq[8] can link these heterogeneities to differences in spatial composition and cellular proximity. However, apart from Niche-seq[7], they have either been applied only in vitro, or rely on generating tissue sections[3–5,8,10], which has confined their applicability to tissues that are easily sectioned. They map the gene expression profiles onto two-dimensional images, and extrapolation to three-dimensional architecture of intact tissues is still limited. For example, 3D expression profiles and cell segmentation have been demonstrated in tissue sections[11,12] but the section thickness is limited by mRNA probe diffusion. In addition, tissue sections can provide only static images, necessitating the use of indirect methods such as pseudo-time analysis to infer cellular trajectories over time[13,14], and none of the currently available spatial transcriptomics technologies have been combined with in vivo imaging.

Here, we present a detailed protocol for using Image-seq, a new platform that enables image-guided cell isolation for single-cell RNA sequencing. The technology is compatible with high-throughput platforms (i.e. 10X Genomics) which enable characterization of abundant bone marrow cell types such as immune cells, along with highly sensitive analysis protocols (i.e. Smartseq) that enable studying rare stromal cell subsets and hematopoietic cell populations. The core of the Image-seq platform is a multiphoton microscope with two optical paths, one for imaging and one for laser micromachining, which creates an access channel in tissue through which a micropipette is brought to the target location and aspirates cells under image guidance. Because it captures viable cells, it can be combined with state-of-the-art library preparation protocols, leading to higher mRNA detection efficiencies and broader transcript coverage than other spatial sequencing technologies. In addition, standard computational tools can be used for data analysis.

# Table of Contents

1. System requirements
2. Visualizing the tissue for Image-seq
3. IACUC approval
4. In situ imaging of long bones
5. In vivo imaging (calvarium description only, although other tissues possible)
6. Image-seq procedure
7. 10x procedure
8. SMARTseq-v4 procedure
9. Data analysis
10. References

## 1. System Requirements

### Imaging Modality

Either a confocal or multiphoton microscope can be used to visualize the 3D spatial organization of bone marrow (BM) tissue.

### Ablation Modality

Requires a scanning device that steers the laser ablation beam across the microscope field of view. This has similar characteristics as the scanning optics used in some multiphoton microscopes (in fact, in our system, the same scanning optics are used for imaging and laser ablation). Consult with your microscope core facility or with us on how to install such a system. The ablation laser should fulfill the following requirements: repetition frequency between 100 kHz to 5 MHz (the latter is used on our system) and a pulse energy of ~50-100 nJ. Note that while we typically use between 10-20 nJ pulse energy for the Image-seq procedure, the laser itself should have higher pulse energies because typical optical systems incur energy losses as the beam propagates to the image plane of the microscope. If you cannot control the ablation geometry by arbitrarily positioning the scanning angle of the device, a variable aperture should be inserted into the intermediate image plane to control the ablation geometry.

### Flushing system

A flushing system should be installed on the objective lens of the multiphoton/confocal microscope. Drawings of the holder are available upon request. The holder secures two blunt-tip needles (22G, Grainger scientific, 5FVC4) across from one another (efflux from one needle is the influx to the other needle, see Figs. 1 and 10c) so that a steady stream of PBS or saline can flow across the sample during the ablation procedure and remove any gas and debris that is generated. Flow is achieved using a dual-channel peristaltic pump (Cole-Parmer, EW-78001-58) with tubing (ISMATEC, SC0330), needles are attached to one end of the tubing.

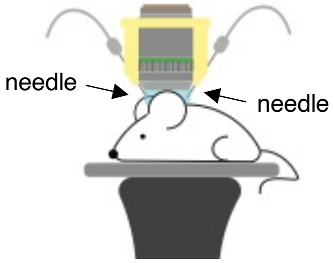


**Figure 1**: **Flushing system.** A 3D-printed holder secures two blunt-tipped needles on the objective lens. They are used to flush a continuous stream of PBS across the sample (in this case a mouse skull) during the laser ablation procedure.

### Micromanipulator for Micropipette

The micropipette holder should be mounted onto a motorized micromanipulator (Sutter Instrument, MPC-385), and connected to an air syringe (Cooper Surgical, 6-34-520) with polythene tubing (Cooper Surgical, 6-34-536). To aid with quickly translating the micropipette to the bone marrow and pulling it back out to eject the collected sample into a tube, the micromanipulator should be mounted onto a sliding stage. A sliding stage can be made by removing the actuator from a translational stage and sliding in a post (L=50 mm) as a spacer, thereby achieving an IN (post in) or OUT (post out) position (see Fig. 2 below). We recommend using a 50 mm travel linear translation stage (Thorlabs, XR50P). It should be positioned so that the micropipette appears in the center of the field-of-view when the micromanipulator is moved to the "IN" position.

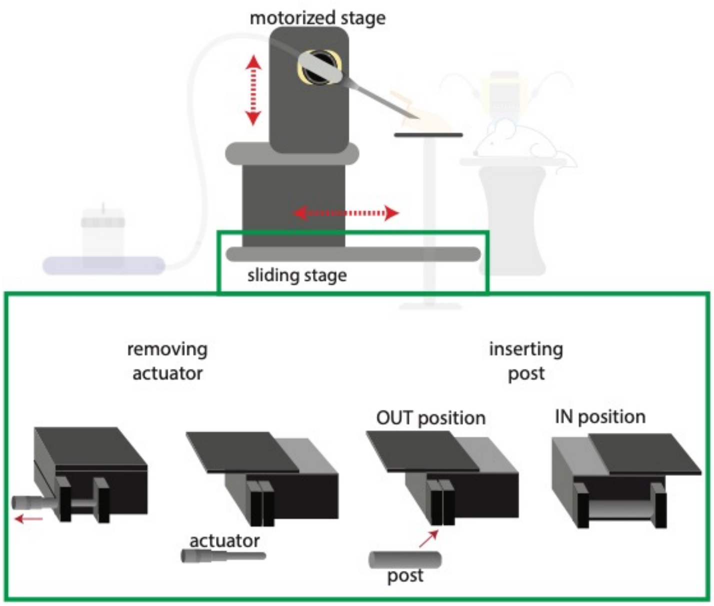


**Figure 2**: **Micromanipulator that is mounted on a sliding stage.** The sliding stage has an IN (post IN and micropipette IN the microscope field of view), as well as an OUT position (post is OUT, along with the micropipette). It is created by removing the actuator from a translational stage (see text for details).

2. **Visualizing the tissue for Image-seq**
Multiple contrast mechanisms can be used to visualize the procedure and reconstruct the 3D spatial position of the extracted cell sample, including autofluorescence, confocal reflectance or labeling with fluorescent membrane dyes such as DiD/DiR/DiI/Di8-ANEPPS or a fluorescent antibody. If a *single cell* or a specific cell population is to be isolated, then either transgenic expression or fluorescent antibody labeling of this subpopulation is necessary so that after aspiration of the target cell(s) and 100-400 surrounding cells, the target cells can be isolated by flow cytometric sorting. For transplantation models, cells can be labeled prior to injection. If not working with a fluorescent reporter mouse or using confocal reflectance to visualize the tissue, we recommend injecting Di8-ANEPPS (1.9 mg/kg animal weight) prior to the Image-seq procedure.

3. **IACUC approval**
Prior to performing any of the experiments detailed below, an animal protocol describing these procedures should be submitted to and approved by the institutional animal care and use committee (IACUC). Experiments should be carried out in accordance with the guidelines set forth for the procedures on rodents at the home institution. Administer analgesics in accordance with your institution's IACUC policy and consult an OAR/IACUC veterinarian.

4. **In situ imaging of long bones**

**Materials**
- Standard surgical kit (for example Fisher Scientific, 50-822-920)
- Kimtech wipes (Fisher Scientific, 06-666)
- Microscope slide (Fisher Scientific, 12-550-15)
- Modelling clay (Amazon, B0025Z8H7Q)
- Tibia (or femur) bone

- Razor blade (ThermoFisher, S17302)

A. Euthanize the animal and dissect the tibia (or femur bone, or any other type of bone) from the animal. Use Kimtech wipes to remove excess muscle tissue from the isolated bone.

B. Carefully scrape off superficial bone tissue from the bone surface with a razor blade (or a micro drill) until the bone marrow becomes visible (see Figs. 3 and 4 below), this typically occurs at a bone thickness of ~50 $\mu$m. Hydrate the bone using a drop of PBS to maintain cell viability.
*Note*:
- Make sure the bone marrow is not damaged during this step.
- Aim for a bone tissue thickness of ≈50 µm or less (see Fig. 5).

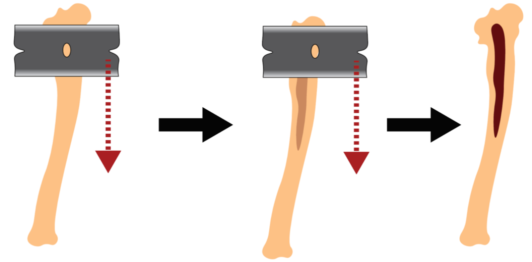

**Figure 3**: **Bone thinning procedure (schematic).** A razor blade is advanced parallel to the bone surface in a single direction to remove bone material. This step is repeated until the red bone marrow is visible and the desired bone thickness is achieved.

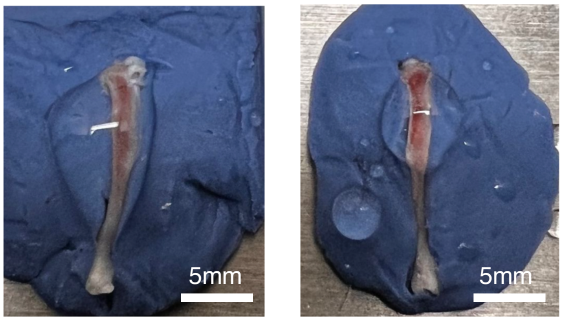


**Figure 4**: **Camera images of un-thinned (left) and thinned (right) tibia bone**. The bone marrow has a redder hue after thinning. Note: PBS drops have been added to the bone to ensure sample integrity.

C. Mount bone sample on a microscope slide by gently pressing the bone into the modelling clay (blue in Fig. 4 above). Deform the modelling clay so that the area to be imaged is parallel to the image plane of the microscope and the glass slide (see Fig. 6).
*Note:*
Alternatively, instead of modelling clay, paraffin can be used as a mounting material. However, once the paraffin has hardened it is more challenging to deform.

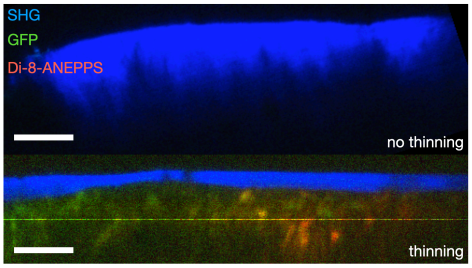


**Figure 5**: **Cross-section of a two-photon microscopy image from an un-thinned (top) and thinned (bottom) tibia bone**. Bone marrow cells cannot be resolved for an un-thinned sample. Scale bar: 50 µm.

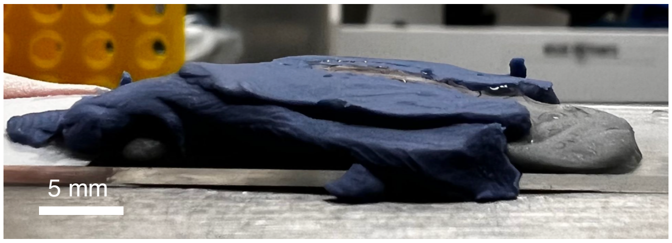


**Figure 6**: **Side-view of the bone mount.** Region of interest is positioned parallel to the surface of the glass slide.

## 5. In vivo imaging of the calvarium

Detailed online protocols and videos have been published on the procedure for in vivo imaging of the calvarium[15–17]. We therefore provide only a brief outline below.

### Materials

- Standard surgical kit (for example Fisher Scientific, 50-822-920)
- Anesthetics and analgesics in accordance with your institution's IACUC
- Mouse holder with integrated heating pad (drawing available upon request)
- Razor blade (ThermoFisher, S17302)
- Ethilon 6-0 nylon monofilament sterile sutures (Zogo Medical, ETH-1956G)

- Curad antibiotic ointment (ATC medical, PF11102)
- Betadine swabsticks (Fisher Scientific, 19-061617)
- Sterile cotton swabs (Fisher Scientific, 18-366-472)
- Phosphate-buffered saline, PBS (ThermoFisher Scientific, 10010002)

**Procedure**

1. Prior to beginning the experiment, install the correct dichroic mirrors and filters for visualizing and separating the emission spectra of the fluorophores of interest. Tools like ThermoFisher's Fluorescence spectra viewer (https://www.thermofisher.com/order/fluorescence-spectraviewer/) or BioLegend's spectra analyzer (https://www.biolegend.com/en-us/spectra-analyzer) can be used to find optimal choices.
2. Administer analgesics ~30-60 min prior to imaging (we use buprenorphine IP at 0.05-0.1 mg/kg animal weight). Anesthetize the mouse (we use vaporized isoflurane at 3-4% for induction and 1-2% for maintenance), ensuring depth of anesthesia by toe pinch. Hydrate the mouse's eyes.
3. Transfer the mouse to a holder with integrated heating pad and anesthesia supply.
4. Trim the hair around the incision site using a sterile razor blade or surgical scissors and scrub with betadine.
5. Make a ~ 5 mm x 7 mm incision into the skin (see Fig. 7) using sterile surgical scissors, taking care to point their tip upwards to avoid damaging the skull or eyes.
6. Fold back the skin flap, secure it to the back of the skull using a drop of antibiotic ointment and expose the skull bone. Hydrate it with a drop of sterile PBS, taking care to keep the skin flap hydrated as well.
7. Remove the periosteum by gently rubbing two sterile cotton swabs across the skull in a concerted motion, starting from the interfrontal/sagittal suture and moving the swabs outwards.
8. Transfer the mouse to the multiphoton/confocal microscope. Secure in such a manner that the skull surface is mounted parallel to the image plane of the microscope.
9. Record the position of lambda and bregma (see Fig. 8): they will serve as reference points for longitudinal imaging experiments.

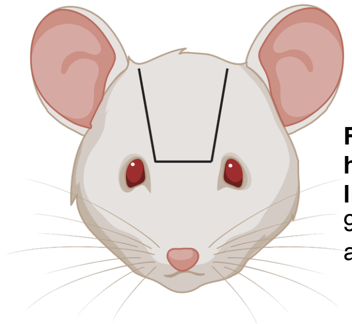

**Figure 7**: **Schematic of mouse head with incision site (black lines)**. Typical dimensions are 7-9 mm in length and 3-5 mm across.

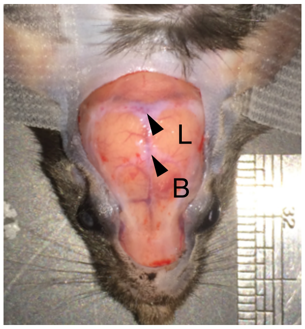


**Figure 8**: **Camera image of mouse skull**. Position of Lambda (L) and Bregma (B) marked by arrowheads.

10. Perform IVM experiments and record the position of the imaging site with respect to Bregma and Lambda. We often record z-stacks with a 2 $\mu$m step size and 15-30 frame average. To study short-term dynamics, it is helpful to perform time-lapse microscopy and record images or short z-stacks with a time interval of a few seconds or minutes, or up to a few hours. If this is a terminal experiment, sacrifice the animal after finishing the imaging session.

    *For survival experiments*:

11. Once the imaging session is completed, flush the skull extensively with sterile saline. Close the scalp with surgical sutures and infiltrate 0.25% Bupivacaine (2mg/kg) into the surgical site to aid with pain management. Finally, apply triple antibiotic ointment (Curad) on the sutured area. Return the animal to its cage and monitor it until awake. Administer buprenorphine (0.05-0.1mg/kg animal weight) either IP or SQ along with topical antibiotic ointment every 8-12 hours for up to two days after the surgery.

## 6. Image-Seq procedure

**Equipment**

- Peristaltic pump (Cole-Parmer, EW-78001-58)
- Confocal/Multiphoton microscope
- Ablation modality (see section 1)
- Air syringe (Coopersurgical, 6-34-520)
- Polythene tubing (Cooper Surgical, 6-34-536)
- Micromanipulator (Sutter Instrument, MPC-385)
- Sliding stage (see section 1)

**Materials**

- Sigmacote (Sigma-Aldrich, SL2-100ML)
- Non-stick, RNAse free microfuge tubes (ThermoFisher Scientific, AM12450)
- Micropipette: 20°-angled blastomere biopsy pipette, 32-37 $\mu$m ID (Origio/Cooper Surgical, MBB-FP-M-20)
- DNAse/RNAse-free deionized water (ThermoFisher Scientific, 10977015)
- 70kDa MW fluorescent dextran, for example Tetramethylrhodamine (ThermoFisher Scientific, D1819)
- Falcon tubes (Fisher Scientific, 14-432-22 and 05-527-90)
- Medium 199 (Gibco, 11150059)
- Fetal bovine serum (FBS, Gibco, A3160501)
- RNAse away (Fisher Scientific, 21-236-21)
- Di8-ANEPPS (ThermoFisher Scientific, D3167)
- Phosphate-buffered saline, PBS (ThermoFisher Scientific, 10010002)
- Sterilized (autoclaved) surgical equipment and perfusion tubing (ISMATEC, SC0330)
- Collagenase I (Sigma, C0130)
- Collagenase XI (Sigma, C7657)
- Hyaluronidase (Sigma, H3506)
- DNase I (Sigma, D5319)
- EDTA (ThermoFisher Scientific, AM9260G)
- 25G Hypodermic Needles, (Fisher Scientific, 14-826AA)
- Permanent marker

**Prior to executing the Image-seq cell isolation**

A. *Coat the micropipette*

To prevent cells from sticking to the glass surface of the micropipette it is necessary to coat it with a siliconizing agent that prevents protein adhesion. It is also recommended to fluorescently coat the pipette for visualization by multiphoton/confocal imaging. This can be achieved as follows:

- Install a fresh set of tubing onto both channels of the peristaltic pump described above, attaching the micropipette to one end. Hold the micropipette into a Falcon tube filled with 1-5 mL of Sigmacote. Allow the Sigmacote to flow **into** the micropipette through its tip, setting the flow rate to ~200$\mu$l/min, and allowing it to flow continuously for 2 min. The waste can be collected by inserting the other end of the tubing into an empty Falcon tube.
- Transfer the micropipette to the other channel and flush it with sterile, deionized (RNAse-free) water using the same flow speed and direction. We typically coat many micropipettes at once and store them for future use.
- Prior to your experiment, install the micropipette on your multiphoton microscope, and flow a fluorescent dextran with your color of choice into the micropipette using the air syringe. Pipette the dextran up and down for 2-3 min before expelling the liquid and gently drying the tip with a piece of lens tissue. The micropipette is now ready for use. Note that we do this final step directly before the experiment.

B. *Prepare microfuge tubes for ejecting the samples:* Clean the workspace with RNAse Away. Pipette 5µl of Medium-199 supplemented with 2% FBS into enough microfuge tubes to collect all micropipette samples. Store them on ice. Clean the microfuge holder (Fig. 11) with RNAse Away.

C. *Visualization*: If you are not working with a fluorescent reporter mouse/fluorescent transplant model, it is necessary to inject either Di8-ANEPPS (1.9 mg/kg animal weight) or a fluorescent antibody to aid in the visualization of the Image-seq procedure and/or label the target cell population. Wait at least 30 min after injection to ensure proper labeling.

D. *Perfusion*:
Unless doing *in vivo cell isolation*, mice should be perfused prior to sample isolation. This minimizes the RBC content and further processing steps. General procedures for rodent perfusion have been described in detail elsewhere, along with relevant videos[18]. We detail our adaption below:

- Prepare 40ml of 5mM EDTA/PBS in a Falcon tube, along with 40ml of regular PBS (w/o Ca/Mg).
- Store both solutions on ice.
- Set the flow rate of the peristaltic pump to 5ml/min and attach a 25G needle to one end of the tubing
- Use a permanent marker to mark the needle ~5mm from its tip (note that the exact distance depends on the size, and therefore the age and gender, of the mouse heart). This is to ensure that the needle is not injected too deeply into the mouse heart during the perfusion procedure.
- Transfer mouse to a dissection tray and set the anesthesia to 4-5% vaporized isoflurane.
- Shave the chest.
- Place one end of the peristaltic tubing into the solution of EDTA/PBS, turn on the pump and wait until liquid is flowing out of the syringe. Turn off the pump.
- Scrub the chest with betadine, make an incision and cut off the skin in the region surrounding the ribcage (Fig. 9a below). This is to aid in the visualization of the procedure.
- Cut open the ribcage parallel to the sternum, at a distance of ~10 mm on either side. Fold up the ribcage towards the mouse head and gently move the beating heart with a pair of tweezers to expose the right atrium. Make an incision into the right atrium and ensure that blood is flowing into the chest cavity.
- Use a pair of tweezers to gently reposition the heart and facilitate insertion of the needle into the apex of the left ventricle. Turn on the pump and hold the needle in place with a pair of tweezers (Fig. 9b).
- Perfuse with 10 ml of ice-cold PBS/EDTA followed by 10 ml of ice-cold PBS.
- Mount the mouse in a holder or dissect the calvarium/tibia and mount it onto a glass slide as described in section 4.

a) b)

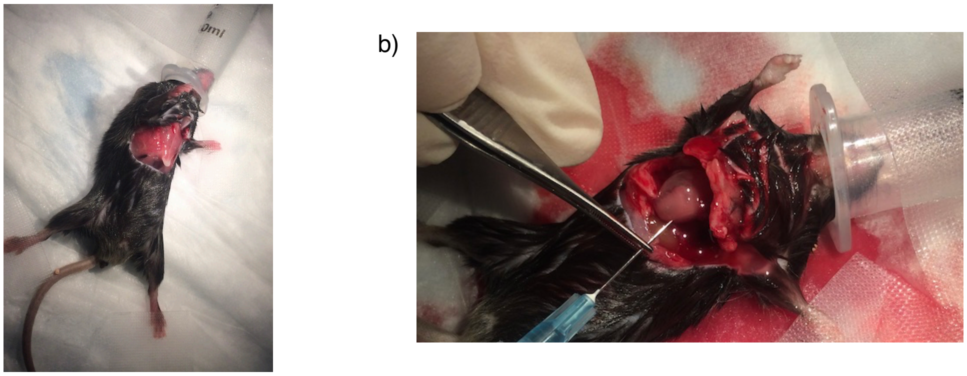

**Figure 9**: **Camera images of perfusion procedure**. a) Skin has been removed to aid in the visualization of the surgical procedure. b) Needle is inside the left ventricle and the mouse is being perfused.

E. *Perfusion for the isolation of stromal cells*:
This procedure is nearly identical to the one described above. However, instead of perfusing with ice-cold EDTA/PBS, the mouse is first perfused with 10ml EDTA/PBS at 37°C, then with 10ml of an enzymatic digestion buffer at 37°C. This buffer consists of 450 U/mL Collagenase I (Sigma), 125 U/ml

Collagenase XI (Sigma), 60 U/ml Hyaluronidase (Sigma), 60 U/ml DNase I (Sigma) in 20 ml of Medium-199 (Gibco). The mouse is then incubated at 37°C for 20 min and the calvarium or long bone dissected and mounted.

**Laser ablation and cell aspiration by micropipette**

After positioning the sample (this can be either a live mouse or a mounted calvarium/long bone) in the image plane of the microscope and finding the target location, turn on the peristaltic pump and flow PBS across the sample at a flow rate of 10 ml/min.

A. Check the thickness of the bone by translating the sample along the z-dimension, using the second-harmonic generation signal (SHG signal, collected at half the wavelength of the multiphoton laser) to visualize the bone.
B. Set the laser pulse energy to 14 nJ at the sample and thin a ~200x300 $\mu$m area of bone, leaving a layer that has a minimum thickness of 20$\mu$m, thereby ensuring minimal damage to the bone marrow located beneath (see Fig. 10b for a 3D representation of what the generated ablation crater typically looks like). The ablation procedure itself is performed at a rate of 0.25 $\mu$m/670 ms along the z-dimension, which corresponds to 10 passes per plane using the 15 frame per second imaging rate of our optical system.
C. Set the laser pulse energy to 11 nJ and ablate a ~60x100 $\mu$m microchannel (Fig. 10a,b) that creates access to the bone marrow tissue, taking care to remove all bone but to avoid ablating bone marrow tissue. In doing so, make sure to position the top of the ablation crater at a distance of 20-30$\mu$m from the target cell(s) (see Fig. 10a).
D. Move the sample down (z-dimension) and slide in the micropipette ("IN" position), visualizing its tip in the microscope field-of-view.
E. Move the sample (=mouse in Fig. 10c) back up and position the micropipette exactly above the microchannel but without touching it. Using the micromanipulator, insert the micropipette through the microchannel and position it next to the target cell(s) (see Fig. 10a). Note that the micropipette has a ~20° angle with respect to the sample plane (see Figs. 10a, c).

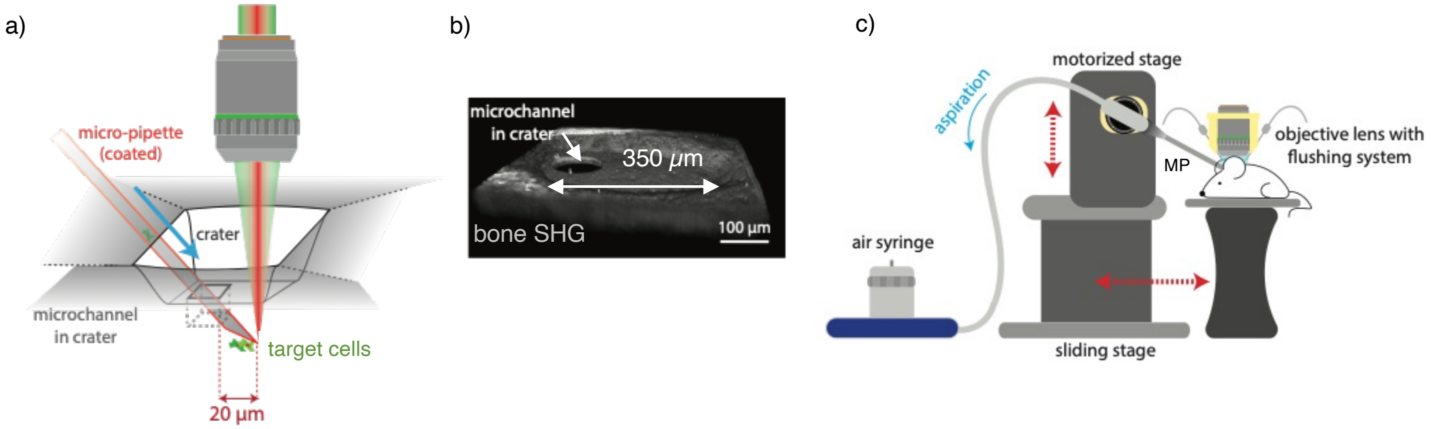


**Figure 10**: **Laser ablation and insertion of micropipette for cell aspiration**. a) Placement of the microchannel at a 20$\mu$m distance to the target cells, along with inserted micropipette. b) 3D representation of a 3D volume of bone after the laser ablation procedure. Bone signal is visualized by second harmonic generation (SHG) of the incident wavelength. c) Schematic of a micropipette (MP) that has been inserted into the bone marrow, along with the air syringe used for cell aspiration.

F. Aspirate the target cells while recording a video by gently suctioning with the air syringe (Fig. 10c). The amount by which the air syringe is rotated controls the overall volume that is displaced and therefore the total number of cells that are aspirated.
G. Move the sample down and slide out the micropipette ("OUT" position).
H. Place a microfuge tube filled with Medium-199/FBS onto a holder and insert the micropipette into the tube liquid. Expel the aspirated cell sample, directly generating a single-cell suspension (see Fig. 11 below).
I. Remove the tube and place it on ice.

J. Repeat the procedure to collect more Image-seq samples. To ensure high-quality single-cell data, ensure that the entire procedure (from mouse perfusion to multiple sample pickings) does not take longer than ~2.5h. In this time-frame we typically collect 4-6 samples.

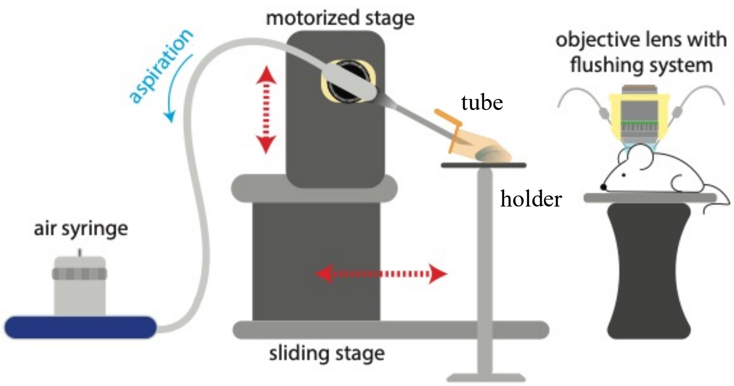


**Figure 11**: **Ejecting the BM aspirate into a microfuge tube**. After aspiration, the micropipette is removed from the sample using the sliding stage and the aspirated BM is ejected into a microfuge tube.

In general, the number of Image-seq samples that are required to obtain statistical significance is given by the size of the biological effect and the observed standard deviation. If there is no sequencing data available in the literature to estimate the size of the biological effect, we recommend running a small pilot with N=3 Image-seq samples to quantify it along with the standard deviation, and, if necessary, to collect additional samples until statistical significance is achieved. Ideally samples should be collected from at least 3 different mice. Depending on your biological question, use either the 10x genomics platform for cell encapsulation and library preparation or the SMARTseq-v4 protocol.

## 7. 10x procedure

### Materials

- Image-seq sample(s) isolated in section 6
- Medium 199 (Gibco, 11150059)
- Ambion UltraPure Bovine serum albumin (Fisher Scientific, AM2616)
- Gibco Trypan Blue solution, 0.4% (Fisher Scientific, 15-250-061)
- Hemocytometer (iNCYTO, DHC-B02)
- Low binding pipette tips, p20 (Rainin, 30389226)
- 10X Chromium Single Cell analysis kit of choice

### Procedure

A. All of the droplet-based sequencing data generated for the Image-seq paper used 10X Genomics v2 kits that are no longer available for purchase. Depending on your sequencing needs consult with your local sequencing core or 10X Genomics representative regarding what type of 10X Chromium Single Cell analysis kit will best suit your needs.

B. Check the final volume in each microfuge tube from the Image-seq cell isolation procedure described above. At this stage there are two considerations. Firstly, the sample should be in a volume that is compatible with your 10X Chromium Single-Cell analysis kit of choice. Therefore, consult your kit's user manual (https://www.10xgenomics.com/). Secondly, the overall cell number needs to be determined. For counting cells in the disposable hemocytometer, the minimal required volume is 5μl. Adjust the volume of your samples so that 5μl can be safely removed without depleting the sample for the downstream sequencing step. If sample size is not a limiting factor, a larger volume of the sample can be used to perform the cell count.

C. Mix the cell suspension in a 1:1 ratio with 0.4% trypan blue.

D. Load the cells onto the hemocytometer (iNCYTO) and count live and dead cells. Also, take note if there are cell aggregates. If there is extensive aggregation, additional dissociation of the sample could be required to avoid clogging the 10x Chromium Chip.

E. Consult the cell loading table in the Chromium Single Cell 3' user guide. Using 0.4mg/ml ultra-pure BSA in Medium 199 adjust the cell concentration accordingly.

F. Place the cells on ice until ready to load on the 10X Chromium Chip.

G. For preparation of your scRNA-seq samples, follow the instructions provided by 10X Genomics for your kit of choice (https://www.10xgenomics.com/).

H. In order to prepare scRNA-seq libraries for sequencing, consult with your sequencing core or provider to determine preferred submission format.

**8. SMARTseq-v4 procedure**

If SMARTseq-v4 library preparation is chosen for processing the samples, it is necessary to first sort single cells into individual wells of a 96-well plate using a flow cytometer or microfluidic sorting device. To aid with drawing the correct gates, it is recommended to collect whole bone marrow preparations from one of the bones that were not processed by Image-seq. For our experiments, the Bauer Core at Harvard University performed library preparation and sequencing using the SMARTseq-v4 Ultra Low Input RNA kit. We recommend using your core facility or a company to perform library preparation and sequencing since the procedure can be time-consuming and tedious.

**Equipment**

- Flow cytometer
- Any equipment detailed in the SMART-Seq® v4 Ultra® Low Input RNA Kit for Sequencing User Manual (https://www.takarabio.com/a/114896).

**Materials**

- Image-seq sample(s) isolated in section 6
- Optional: antibody for staining a subpopulation of cells
- Medium-199 (Gibco, 11150059)
- FBS (Gibco, A3160501)
- DAPI (BioLegend, 422801)
- Phosphate buffered saline, PBS (ThermoFisher Scientific, 10010002)
- SMARTseq-v4 Ultra Low Input RNA Kit for Sequencing (Takara Bio, 634891)
- Any further reagents listed in the SMART-Seq® v4 Ultra® Low Input RNA Kit for Sequencing User Manual (https://www.takarabio.com/a/114896).

**Sort**

A. Pipette sample up and down several times to generate a single-cell suspension.
B. Stain sample with antibody for 30 minutes at 4°C in Medium 199 supplemented with 2% FBS, using the antibody concentration recommended by its manufacturer.
C. Add 1 ml of PBS to the sample, as well as 0.1 $\mu$g of DAPI and incubate the sample for 10min.
D. Gently vortex the cell sample and transfer it to the flow cytometer (MoFlo Astrios EQ cell sorter for our experiments).
E. Use a whole bone marrow preparation processed in the same manner to draw initial gates for flow cytometric sorting.
F. Sort single, live cells into individual wells of a 96-well PCR plate filled with 2.6 $\mu$l of lysis buffer (Takara Bio USA, Inc.).
G. After completing the sort, seal plates, spin them down and snap freeze them.

H. Store plates at -80°C prior to preparation for cDNA synthesis using the SMARTseq-v4 assay. Note that it is possible to bank samples until a sufficient number of cells have been collected and perform library preparation on all of the collected cells simultaneously.

**Library preparation**

A detailed description of the cell-lysis, reverse transcription, library preparation and indexing steps can be found in the SMART-Seq® v4 Ultra® Low Input RNA Kit for Sequencing User Manual by Takara Bio (https://www.takarabio.com/a/114896).

**Sequencing**

In order to prepare scRNAseq libraries for sequencing, consult with your sequencing core or provider to determine preferred submission format.

## 9. Data Analysis

The 10X scRNA-seq data and SMARTseq-v4 data described in the Image-seq manuscript have been deposited into the Gene Expression Omnibus (GEO) database under GSE188902. Custom code that was used in the study can be found on github at: https://github.com/shenglinmei/Image-seq or Zenodo (https://zenodo.org/record/7067661).

**Operating system**

This protocol assumes users have a Unix-like operating system (i.e., Linux or MacOS X), with a bash shell or similar. All commands given here are meant to be run in a terminal window. The R introductory message will start with a '>' prompt.

**Align the scRNA-seq reads to the genome**

For the 10X scRNA-Seq data, Reads were aligned to the mm10 reference genome (https://support.10xgenomics.com/single-cell-gene-expression/software/downloads/latest?) using the Cellranger pipeline (version 3.0.2, 10x Genomics). For SMARTseq-v4 data, reads were aligned with hisat2[19] and featureCounts[20] was used to calculate read counts.

A. Map the 10X scRNA-seq reads for each sample to the reference genome and read counts quantification:

```
FQ = =${INPUTDIR}
cellranger count --id $FQ \
    --fastqs=$FQ / \
    --transcriptome=refdata-cellranger-mm10-1.2.0 \
    --localcores=50 \
    --localmem=250
```

B. Map the SMARTseq-v4 sequencing data for each cell to the reference genome and read counts quantification:

```
OUTPUT=${SAMPLE}.genome
FQ1=${INPUTDIR}/${SAMPLE}_R1_001.fastq.gz
FQ2=${INPUTDIR}/${SAMPLE}_R2_001.fastq.gz

hisat2 -t \
    -x ${REF} \
    -1 ${FQ1} \
    -2 ${FQ2} \
    --rg-id=${SAMPLE} --rg SM:${SAMPLE} --rg LB:${SAMPLE} \
    --rg PL:ILLUMINA --rg PU:${SAMPLE} \
    --new-summary --summary-file ${OUTPUT}.log \
    --met-file ${OUTPUT}.hisat2.met.txt --met 5 \
    -k 10 \
    --secondary \
```

```
--seed 12345 \
-p 4 -S ${OUTPUT}.bam

featureCounts -T 5 -p -t exon -g gene_name  -a $gtf -o  ${SAMPLE}
${INPUT_DIR}/${SAMPLE}.genome.bam
```

**10X scRNA-seq data integration**
We used Conos[21] (https://github.com/kharchenkolab/conos Version 1.4.6) to integrate multiple scRNA-seq datasets together. Each individual dataset was first normalized using basicP2proc function in pagoda2[22] with default parameters. Different samples were then aligned using Conos with default parameter settings.

```
>   library(pagoda2)
>   library(conos)
>   raw <-  readRDS('raw.rds')  # read list object of count matrices
>   p2lis2 <- lapply(raw,function(x) basicP2proc(x,n.cores = 10,min.transcripts.per.cell =0))
>   con <- Conos$new(datlp2, n.cores = 10)
>   con$buildGraph()
>   con$findCommunities()
>   con$embedGraph(method = "UMAP", spread = 7)
>   def <- con$getDatasetPerCell()  # get differentially expressed genes
```

**SMARTseq-v4 data integration**
Seurat (https://satijalab.org/seurat/ Version 4.0.6) was used to analyze the SMARTseq-v4 data.

```
>   library(Seurat)
>   marrow <- CreateSeuratObject(counts = cds)
>   marrow <- NormalizeData(marrow)
>   marrow <- FindVariableFeatures(marrow, selection.method = "vst")
>   marrow <- ScaleData(marrow, features = rownames(marrow))
>   marrow <- RunPCA(marrow, features = VariableFeatures(marrow), ndims.print = 6:10, nfeatures.print =
    10)
>   marrow <- FindNeighbors(marrow, dims = 1:30)
>   marrow <- FindClusters(marrow, resolution = 1)
>   marrow <- RunUMAP(marrow, dims = 1:30)
>   DimPlot(marrow, reduction = "umap")
```

**Regressing out cell cycle genes**
Seurat (version 3) was used to regress out cell cycle genes. First, we assigned each cell a score, based on its expression of G2/M and S phase markers with the CellCycleScoring function. Then we applied the ScaleData function to regress out cell cycle genes.

```
>   library(Seurat)
>   exp.count <- readRDS('exp.count.rds')
>   marrow <- CreateSeuratObject(counts = exp.count)
>   s.genes <- cc.genes$s.genes
>   g2m.genes <- cc.genes$g2m.genes
>   marrow <- NormalizeData(marrow)
>   marrow <- FindVariableFeatures(marrow, selection.method = "vst")
>   marrow <- ScaleData(marrow, features = rownames(marrow))
>   marrow <- CellCycleScoring(marrow, s.features = s.genes, g2m.features = g2m.genes, set.ident =
    TRUE)
```

```
> marrow <- ScaleData(marrow, vars.to.regress = c("S.Score", "G2M.Score"), features =
  rownames(marrow))
> marrow <- RunPCA(marrow, features = VariableFeatures(marrow), nfeatures.print = 10)
> marrow <- FindNeighbors(marrow, dims = 1:10)
> marrow <- FindClusters(marrow, resolution = 1)
> marrow <- RunUMAP(marrow, dims = 1:5)
```

**Differential expression**

DESeq2 (pubmed: 25516281) was used for analyzing differentially expressed genes between proliferating (P) and non-proliferating (NP) cells

```
> counts <-  readRDS('count.rds') ## read raw counts
> group <-  group[group %in% c('P','NP')]
> cm <-  as.matrix(counts[,names(group)])
> meta <- data.frame(sample.id = colnames(cm), group =group)
> dds1 <- DESeq2::DESeqDataSetFromMatrix(round(cm,0), meta, design = ~group)
> meta$group <- relevel(meta$group, ref = 'NoPro')
> dds1 <- DESeq2::DESeq(dds1)
> res <- DESeq2::results(dds1, cooksCutoff = FALSE, independentFiltering = FALSE)
> res <- as.data.frame(res)
> res <- res1[order(res $padj, decreasing = FALSE), ]
```

**Visualization of differentially expressed genes**

```
> library(EnhancedVolcano)
> # create custom key-value pairs for 'high', 'low', 'mid' expression by fold-change
> keyvalssize <- ifelse(
>   res$log2FoldChange <= -2 & res$padj < 0.01, 3,
>   ifelse(res$log2FoldChange >= 2 & res$padj < 0.01, 3,
>   1))

> EnhancedVolcano(res, lab = rownames(res), x = 'log2FoldChange', y = 'padj', title=NULL,
> subtitle=NULL, caption=NULL,
> selectLab = rownames(res)[which(names(keyvals) %in% c('high', 'low'))],
> xlab = bquote(~Log[2]~ 'fold change'),
> ylab = bquote(~Log[10]~ 'padj'),
> pCutoff = 0.001,
> FCcutoff = 2,
> pointSize = keyvalssize, #3.5,
> labSize = 4.5,
> #shape = c(6, 4, 2, 11),
> colCustom = keyvals,
> colAlpha = 1,
> legendPosition = 'none',
> legendLabSize = 15,
> legendIconSize = 5.0,
> drawConnectors = TRUE,
> widthConnectors = 1.0,
> colConnectors = 'black',
> arrowheads = FALSE,
> gridlines.major = TRUE,
> gridlines.minor = FALSE,
> border = 'partial',
```

```
>   borderWidth = 1.5,
>   borderColour = 'black')
```

**Clustering of cell cycle signature score**

To assess cell states in different cell subsets and conditions, we used a gene set signature score to measure the relative difference of cell cycle states. The signature scores were calculated as average expression values of the genes in a given set. The signature gene list was downloaded from Whitfield et al.[23] Hierarchical clustering of the cell cycle signature score was used to group AML cells.

```
>   df <-  read.csv(' CellCycleGeneList.txt',sep='\t',header=T)
>   allscore=NULL
>   gl <-  list() #gene list
>   for (i in c("M/G1"  ,  "G2"    ,  "G2/M"  ,  "G1/S"  ,  "S phase" )){
>       tmp1 <-  df[df$PHASE==i,]
>     gs <-  apply(tmp1,1,function(x) strsplit(x['NAME'],' ')[[1]][1])
>     gs <-  gs[gs!='']
>     Gsano <- readRDS('gene.ano.rds') # human and mouse homolog genes
>     index12 <- (gs %in% gsano$Gene_Name)
>     index12 <- match(gs[index12],gsano$Gene_Name)
>     gs2 <-  Toch(gsano[index12,'mouse_homolog_gene'])
>     gl[[i]]=gs2
>     allscore <- cbind(allscore,rowMeans(as.matrix(exp[gs2,])))
>   }
>   Annot <- data.frame('cluster'=tr3,'fraction'=dtype2[names(tr3)],row.names=names(tr3))
>   colnames(allscore) <-  c("M/G1"  ,  "G2"    ,  "G2/M"  ,  "G1/S"  ,  "S phase" )
>   a <- pheatmap::pheatmap(allscore,annotation_row = annot)
>   cluster <- cutree(a$tree_row,k=3)
```

## 10. References


1. Maniatis, S., Petrescu, J. & Phatnani, H. Spatially resolved transcriptomics and its applications in cancer. *Current Opinion in Genetics and Development* vol. 66 70–77 (2021).
2. Hu, K. H. *et al.* ZipSeq: barcoding for real-time mapping of single cell transcriptomes. *Nat Methods* **17**, 833–843 (2020).
3. Baccin, C. *et al.* Combined single-cell and spatial transcriptomics reveal the molecular, cellular and spatial bone marrow niche organization. *Nat Cell Biol* **22**, 38–48 (2020).
4. Linus Eng, C.-H. *et al.* Transcriptome-scale super-resolved imaging in tissues by RNA seqFISH+. *Nature* **568**, 235–239 (2019).
5. Vickovic, S. *et al.* High-definition spatial transcriptomics for in situ tissue profiling. *Nat Methods* **16**, 987–990 (2019).
6. Lee, J. H. *et al.* Fluorescent in situ sequencing (FISSEQ) of RNA for gene expression profiling in intact cells and tissues. *Nature Protocols 2015 10:3* **10**, 442–458 (2015).
7. Medaglia, C. *et al.* Spatial reconstruction of immune niches by combining photoactivatable reporters and scRNA-seq. *Science (1979)* **358**, 1622–1626 (2017).
8. Rodriques, S. G. *et al.* Slide-seq: A scalable technology for measuring genome-wide expression at high spatial resolution. *Science (1979)* **363**, 1463–1467 (2019).
9. Chen, K. H., Boettiger, A. N., Moffitt, J. R., Wang, S. & Zhuang, X. Spatially resolved, highly multiplexed RNA profiling in single cells. *Science (1979)* **348**, (2015).
10. Moffitt, J. R. *et al.* High-performance multiplexed fluorescence in situ hybridization in culture and tissue with matrix imprinting and clearing. *Proc Natl Acad Sci U S A* **113**, 14456–14461 (2016).
11. Wang, X. *et al.* Three-dimensional intact-tissue sequencing of single-cell transcriptional states. *Science (1979)* **361**, (2018).
12. Petukhov, V. *et al.* Cell segmentation in imaging-based spatial transcriptomics. *Nature Biotechnology* **40** 345-354 (2022).

13. Trapnell, C. *et al.* The dynamics and regulators of cell fate decisions are revealed by pseudotemporal ordering of single cells. *Nat Biotechnol* **32**, 381–386 (2014).
14. La Manno, G. *et al.* RNA velocity of single cells. *Nature 2018 560:7719* **560**, 494–498 (2018).
15. Wu, J. W., Runnels, J. M. & Lin, C. P. Intravital imaging of hematopoietic stem cells in the mouse skull. *Methods in Molecular Biology* **1185**, 247–265 (2014).
16. Lo Celso, C., Lin, C. P. & Scadden, D. T. In vivo imaging of transplanted hematopoietic stem and progenitor cells in mouse calvarium bone marrow. *Nature Protocols 2010 6:1* **6**, 1–14 (2010).
17. Scott, M. K., Akinduro, O. & Lo Celso, C. In Vivo 4-Dimensional Tracking of Hematopoietic Stem and Progenitor Cells in Adult Mouse Calvarial Bone Marrow. *JoVE (Journal of Visualized Experiments)* e51683 (2014).
18. Gage, G. J., Kipke, D. R. & Shain, W. Whole Animal Perfusion Fixation for Rodents. *JoVE (Journal of Visualized Experiments)* e3564 (2012).
19. Kim, D., Paggi, J. M., Park, C., Bennett, C. & Salzberg, S. L. Graph-based genome alignment and genotyping with HISAT2 and HISAT-genotype. *Nature Biotechnology 2019 37:8* **37**, 907–915 (2019).
20. Liao, Y., Smyth, G. K. & Shi, W. featureCounts: an efficient general purpose program for assigning sequence reads to genomic features. *Bioinformatics* **30**, 923–930 (2014).
21. Barkas, N. *et al.* Joint analysis of heterogeneous single-cell RNA-seq dataset collections. *Nature Methods 2019 16:8* **16**, 695–698 (2019).
22. Fan, J. *et al.* Characterizing transcriptional heterogeneity through pathway and gene set overdispersion analysis. *Nature Methods 2015 13:3* **13**, 241–244 (2016).
23. Whitfield, M. L. *et al.* Identification of Genes Periodically Expressed in the Human Cell Cycle and Their Expression in Tumors. *Mol Biol Cell* **13**, 1977–2000 (2002).
