# Supplementary material for: Image-seq: spatially resolved single-cell sequencing guided by in situ and in vivo imaging
Source: Nat Methods. 2022 Nov 24;19(12):1622–33. doi: 10.1038/s41592-022-01673-2 (PMC9718684; doi:10.1038/s41592-022-01673-2)
Supplement: Supplementary file 2 — Reporting Summary [file 41592_2022_1673_MOESM2_ESM.pdf]

nature portfolio

Corresponding author(s): Charles Lin, Christa Haase

Last updated by author(s): 09/15/2022

Reporting Summary

Nature Portfolio wishes to improve the reproducibility of the work that we publish. This form provides structure for consistency and transparency in reporting. For further information on Nature Portfolio policies, see our Editorial Policies and the Editorial Policy Checklist.

Statistics

For all statistical analyses, confirm that the following items are present in the figure legend, table legend, main text, or Methods section.

n/a	Confirmed	
☐	☒	The exact sample size (*n*) for each experimental group/condition, given as a discrete number and unit of measurement
☐	☒	A statement on whether measurements were taken from distinct samples or whether the same sample was measured repeatedly
☐	☒	The statistical test(s) used AND whether they are one- or two-sided<br>*Only common tests should be described solely by name; describe more complex techniques in the Methods section.*
☒	☐	A description of all covariates tested
☐	☒	A description of any assumptions or corrections, such as tests of normality and adjustment for multiple comparisons
☐	☒	A full description of the statistical parameters including central tendency (e.g. means) or other basic estimates (e.g. regression coefficient) AND variation (e.g. standard deviation) or associated estimates of uncertainty (e.g. confidence intervals)
☐	☒	For null hypothesis testing, the test statistic (e.g. *F*, *t*, *r*) with confidence intervals, effect sizes, degrees of freedom and *P* value noted<br>*Give P values as exact values whenever suitable.*
☒	☐	For Bayesian analysis, information on the choice of priors and Markov chain Monte Carlo settings
☐	☒	For hierarchical and complex designs, identification of the appropriate level for tests and full reporting of outcomes
☐	☒	Estimates of effect sizes (e.g. Cohen's *d*, Pearson's *r*), indicating how they were calculated

Our web collection on statistics for biologists contains articles on many of the points above.

Software and code

Policy information about availability of computer code

Data collection	2-photon and confocal microscopy was performed either with the Image-seq platform (detailed description in the manuscript) or on a custom- built two-photon excitation microscope equipped with a Ti: Sapphire laser oscillator (Spectra-Physics InSight X3) and coupled with an Olympus 60x water-immersion objective lens (previously described in Yeh et al, Nat. Commun. 2022; Christodoulou et al, Nature 2020; Spencer et al, Nature 2014). Laser scanning and acquisition were controlled by a custom-built software. Spatially-resolved cell isolation was carried out with the Image-seq platform. The Illumina HiSeq 4000 Sequencing System and bcl2fastq (v1.8.4) were used to acquire the 10X data. The sequenced 10X libraries were mapped to the mm10 genome using Cell Ranger software (v 3.0.2). The NextSeq 500 Sequencing System (Illumina) was used to acquire SMART-seqv4 data. SMART-seqv4 sequencing data were aligned with hisat2 (v 4.8.2). Flow cytometry data were acquired on an LSRII flow cytometer, a BD FACS Aria III and a MoFlo Astrios EQ.
Data analysis	We used python and R to perform analysis of sequencing data. Code related to this manuscript can be found at https://github.com/shenglinmei/Image-seq.<br>Cell Ranger software (version 3.0.2)<br>featureCounts (v1.6.4)<br>bcl2fastq (v1.8.4)<br>hisat2 (v4.8.2)<br>DEseq2 (v1.32.0)<br>Seurat (v4.0.6)<br>Pagoda2 (v1.0.10)<br>Conos (v1.4.1)<br>ClusterProfiler (4.0.0)<br>R 4.1.1<br>python 3.7<br>FlowJo 10.8.0<br>Fiji (ImageJ) Version 2.1.0/1.53g was used for image analysis.

GraphPad Prism9 was used for statistical analysis of flow cytometry and imaging data.
CFX Maestro software (Biorad, v1.1) was used for the analysis of QPCR data.

For manuscripts utilizing custom algorithms or software that are central to the research but not yet described in published literature, software must be made available to editors and reviewers. We strongly encourage code deposition in a community repository (e.g. GitHub). See the Nature Portfolio guidelines for submitting code & software for further information.

Data

Policy information about availability of data

All manuscripts must include a data availability statement. This statement should provide the following information, where applicable:

- Accession codes, unique identifiers, or web links for publicly available datasets
- A description of any restrictions on data availability
- For clinical datasets or third party data, please ensure that the statement adheres to our policy

The 10X-seq and SMART-seqv4 data generated in this work have been deposited into the Gene Expression Omnibus (GEO) database GSE188902 (https://www.ncbi.nlm.nih.gov/geo/query/acc.cgi?acc=GSE188902) which is publicly available. The mouse mm10 reference genome was downloaded from 10X genomics (https://support.10xgenomics.com/single-cell-gene-expression/software/downloads/latest?). Public, bulk RNA-seq AML datasets from TCGA (https://cbioportal-datahub.s3.amazonaws.com/aml_ohsu_2018.tar.gz) and OHSU (https://cbioportal-datahub.s3.amazonaws.com/laml_tcga_pan_can_atlas_2018.tar.gz) were download from cbioportal. Due to the extremely large file sizes accompanying the extensive imaging data, raw image data is available from the corresponding authors upon request. Cell lines are available from the authors upon request and mouse lines are commercially available at the Jackson Laboratory. Source data files for all graphs presented in the Figures and Extended Data Figures are linked to the online version of the manuscript.

Field-specific reporting

Please select the one below that is the best fit for your research. If you are not sure, read the appropriate sections before making your selection.

☒ Life sciences ☐ Behavioural & social sciences ☐ Ecological, evolutionary & environmental sciences

For a reference copy of the document with all sections, see nature.com/documents/nr-reporting-summary-flat.pdf

Life sciences study design

All studies must disclose on these points even when the disclosure is negative.

Sample size	For sequencing data: Since this is a new technology and we did not test any hypotheses, no sample size calculation for statistical power of hypothesis testing was performed. Sample sizes were chosen based on the data obtained from a small pilot for each experiment. For flow cytometry data: Sample size was approximated and derived from extensive publications within the hematopoiesis field which are based on previous a priori power testing. No statistical methods were used to pre-determine sample sizes but our sample sizes are in agreement with those reported in previous publications (Yusuf et al. Blood 2020), (van Gastel et. al Cell Metabolism 2020) and (Shah et. al. Nat. Biomed. Eng. 2020). For imaging data: Sample size was approximated by consulting published intravital microscopy datasets of the calvarium bone marrow. The final sample sizes were adequate based on consistency of measured results in each group and are in agreement with those reported in previous publications (Yeh et. al Nat. Commun. 2022), (Christodoulou et al. Nature 2020), and (Spencer et. al. Nature 2014).
Data exclusions	No data was excluded.
Replication	Experimental findings were reliably reproduced. To verify reproducibility of the findings the vast majority of experiments were repeated at least three independent times.
Randomization	We did not allocate samples/organisms into experimental groups, so no randomization was necessary.
Blinding	Analysis of flow cytometry and qPCR data is not subjective and therefore blinding was deemed unnecessary. For the imaging: It is not possible to perform the imaging analysis during the experiment, so it was not possible to pre-select any regions that could have produced a preferred result. Similarly, it is not possible to perform the sequencing data analysis during the experiment, so it was not possible to select any preferred regions for Image-seq cell isolation. In addition, partial blinding was performed since cell isolation, library preparation, sequencing and data analysis were performed by different people.

Reporting for specific materials, systems and methods

We require information from authors about some types of materials, experimental systems and methods used in many studies. Here, indicate whether each material, system or method listed is relevant to your study. If you are not sure if a list item applies to your research, read the appropriate section before selecting a response.

Materials & experimental systems

n/a	Involved in the study
☐	☒ Antibodies
☐	☒ Eukaryotic cell lines
☒	☐ Palaeontology and archaeology
☐	☒ Animals and other organisms
☒	☐ Human research participants
☒	☐ Clinical data
☒	☐ Dual use research of concern

Methods

n/a	Involved in the study
☒	☐ ChIP-seq
☐	☒ Flow cytometry
☒	☐ MRI-based neuroimaging

Antibodies

Antibodies used

Marker Fluorophore Dilution Vendor Catalog# Clone Lot#
CD71 PE/Cy7 1 to 200 Biolegend 113812 RI7217 B259648
Ter119 APC 1 to 100 Biolegend 116212 Ter119 B286863
CD3 BUV737 1 to 100 BD Biosciences 612771 145-2C11 133181
CD11b AF700 1 to 200 Biolegend 101222 M1/70 B308254
CD19 FITC 1 to 200 Biolegend 115506 6D5 8038942
B220 BUV395 1 to 200 B D Biosciences 563793 RA3-6B2 6320735
cKit BV785 1 to 200 Biolegend 105841 2B8 B340832
IgM PE 1 to 200 eBioscience 12-5890-82 eB121-15F9 2134180
F4/80 APC/Cy7 1 to 200 Biolegend 123118 BM8 B274179
Ly6C BV570 1 to 200 Biolegend 128030 HK1.4 B310124
Ly6G BV421 1 to 200 BD Biosciences 562737 1A8 B301980
FC block N/A 1 to 50 BD Biosciences 553142 2.4G2 1279889
CD45 BV421 1 to 100 BioLegend 103134 30-F11 B287242
CD45 APC/Cy7 1 to 100 BD Biosciences 557659 30-F11 1046784
DPP4 PE 1 to 20 Biolegend 137804 H194-112 B319006
Flt3 BV421 1 to 50 Biolegend 135314 A2F10 B329451
Itgb7 BV711 1 to 20 Biolegend 321240 FIB504 B312128
CD48 BUV737 1 to 200 BD Biosciences 565240 HM48-1 8043935
Rat IgG2a k Isotype PE 1 to 20 Biolegend 400508 RTK2758 B290861
NK1.1 APC 1 to 200 Biolegend 108710 PK136 B31103
CD3 APC 1 to 100 eBioscience 17-0031-82 145-2C11 4277755
CD4 APC 1 to 200 Biolegend 100412 GK1.5 B340887
CD8 APC 1 to 200 Invitrogen 17-0081-83 53-6.7 1978219
B220 APC 1 to 200 BD Biosciences 553092 RA3-6B2 B350149
CD19 APC 1 to 200 Biolegend 152409 1D3 B285507
Ter119 APC 1 to 100 eBioscience 17-5921-82 Ter119 E07331-1635
Ly6G AF700 1 to 200 BD Biosciences 561236 1A8 72104
cKit BUV395 1 to 200 BD Biosciences 564011 2B8 337172
CD16/32 BUV7373 1 to 300 BD Biosciences 612783 2.4G2 302289
CD34 FITC 1 to 33 BD Biosciences 553733 RAM34 7083721
DPP4 PE 1 to 20 Biolegend 137804 H194-112 B319006
Sca1 PE/Cy7 1 to 200 Biolegend 108114 D7 B260282
F4/80 Pacific Blue 1 to 200 Biolegend 123124 BM8 B280040
Ly6C BV570 1 to 200 Biolegend 128030 HK1.4 B310124
CD11b BV785 1 to 200 Biolegend 101243 M1/70 B311639
CD115 APC/Cy7 1 to 100 Biolegend 135532 AFS98 B236643
Rat IgG2a k Isotype PE 1 to 20 Biolegend 400508 RTK2758 B290861
Marker Conjugate Dilution Vendor Catalog number Clone Lot #
Ter119 Biotin 1 to 100 Biolegend 116204 Ter119 B336476
CD11b Biotin 1 to 100 Biolegend 101204 M1/70 B340052
Gr1 Biotin 1 to 100 Biolegend 108404 RB6-8C5 B351067
NK1.1 Biotin 1 to 100 Biolegend 108704 PK136 B359276
CD19 Biotin 1 to 100 Biolegend 553784 1D3 553784
BB20 Biotin 1 to 100 BD Biosciences 553086 RA3-6B2 2279903
DPP4 later conjugated to AF568 1 mg/kg (in vivo) BioLegend 137802 H194-112 B316439
IgG2a, kappa Isotype later conjugated to AF568 1 mg/kg (in vivo) BioLegend 400502 RTK2758 B318086
CD31 BV421 1 mg/kg (in vivo) BioLegend 102424 390 B347937

Validation

Only commercially available antibodies were used in this study. All antibodies used for flow cytometry and microscopy were previously validated for the respective application by the vendor. Individual listings of citations using these reagents in the respective imaging or cytometric application and can be found on the manufacturer page which can be accessed using the catalog numbers provided above.

Eukaryotic cell lines

Policy information about cell lines

Cell line source(s)	The HoxA9/Meis1 cell line was kindly provided by the lab of Dr. David Scadden. The MLL/AF9 cell lines were kindly provided by the lab of Dr. David Scadden. NIH3T3 cells, and MC-3T3-E1 cells were purchased from ATCC. MS-5 cells were obtained from RIKEN and MLO-A5 cells were bought from Kerafast.
Authentication	No authentication was performed
Mycoplasma contamination	Cell lines tested negative for mycoplasma contamination.
Commonly misidentified lines (See ICLAC register)	No commonly misidentified cell lines were used in this study.

Animals and other organisms

Policy information about studies involving animals; ARRIVE guidelines recommended for reporting animal research

Laboratory animals	Male and female 8wk-old C57Bl6/J mice (#000664) or male and female CXCL12-DsRed mice (#022458) were ordered from the Jackson Laboratory, housed in our animal facility for at least 2 weeks and used for experiments between 10-14 weeks of age. 8-week old female Fezh/J mice (B6J.129(Cg)-Gt(ROSA)26Sortm1.1(CAG-cas9*,-EGFP), catalog number 026179) were ordered from the Jackson laboratory and used for experiments. Male and female beta-actin-GFP mice (Jackson #006567) were bred in house and used between 10-16 weeks of age. Male and female beta-actin-DsRed mice (Jackson #006051) were bred in house and used between 10-16 weeks of age. β-actin luciferase (βact) mice from Taconic (#11977) were bred with ubiquitin-c-GFP (UcGFP) mice from the Jackson laboratory (#004353) to generate βact-UcGFP transgenic mice. An 8-week old female βact-UcGFP mouse was then used for generating the HA9M1 cell line. All mice were housed in the pathogen-free MGH Animal Facilities, which were equipped with ventilated micro-isolator cages. Sentinel programs and veterinary oversight were in place. Mice were provided with standard chow and drinking water ad libitum. An automated 12h dark - 12h light cycle was observed and mice were housed at a fixed temperature (70 Fahrenheit) and humidity (66%). The MGH Animal Facility is under supervision of the MGH Center for Comparative Medicine. All facilities are fully accredited by AAALAC (#000809) and meet NIH standards as set forth in the "Guide for Care and Use of Laboratory Animals" (DHHS).
Wild animals	No wild animals were used in this study.
Field-collected samples	No field-collected samples were used in this study.
Ethics oversight	All procedures involving animals were carried out in agreement with protocols 2012N000190, 2007N000148 or 2016N000085 that were approved by the Institutional Animal Care and Use Committee of Massachusetts General Hospital and followed the guidelines set forth in the US National Institute of Health Guide for the Care and Use of Laboratory Animals. Animals were housed in a 12h light/dark cycle at a constant temperature and humidity and with ad libitum access to food and water.

Note that full information on the approval of the study protocol must also be provided in the manuscript.

Flow Cytometry

Plots

Confirm that:

☒ The axis labels state the marker and fluorochrome used (e.g. CD4-FITC).

☒ The axis scales are clearly visible. Include numbers along axes only for bottom left plot of group (a 'group' is an analysis of identical markers).

☒ All plots are contour plots with outliers or pseudocolor plots.

☒ A numerical value for number of cells or percentage (with statistics) is provided.

Methodology

Sample preparation	Flow cytometry analysis of WBM and micropipette<br>WBM and micropipette samples from calvarium and tibia were blocked with anti-mouse Fc block (BD Biosciences, dilution 1 to 50) for 10 minutes at 4°C. The cells were thereafter stained with Blood cell lineage cocktail (Table S6) for 30 minutes at 4°C. For detection of dead cells 7AAD (BD Biosciences, 0.25µg) was added to the sample prior to analysis. Flow cytometric analysis was performed on a BD FACS Aria III sorter (BD Biosciences) and all data was analyzed using FlowJo software package (Treestar).<br><br>Flow sorting of AML cells for SMARTseq-v4<br>Prior to sorting, 1ml of PBS (ThermoFisher Scientific) was added to each sample tube, along with 0.1µg of DAPI (ThermoFisher Scientific). The sample was incubated for 10 min, gently vortexed and transferred to the flow cytometer (MoFlo Astrios EQ cell sorter). Single, live, GFP+ AML cells (see Figure S1 for examples of gating strategy) were sorted into individual wells of a 96-well PCR plate filled with 2.6µl of Lysis Buffer (Takara Bio USA, Inc.). Plates were sealed, spun down, snap-frozen and stored at -80°C prior to preparation for cDNA synthesis using the SMARTseq-v4 assay.

Flow analysis and sorting of CXCl12+ stromal cells for SMARTseq-v4
Prior to sorting or analysis, samples were cell-surface stained with anti-CD45-BV421 (BioLegend, dilution 1 to 100) for 30 minutes at 4°C in Medium 199+ (Gibco) supplemented with 2% FBS. Prior to sorting, 1ml of PBS (ThermoFisher Scientific) was added to each sample tube, along with 0.1µg of DAPI (ThermoFisher Scientific). The sample was incubated for 10 min, gently vortexed and transferred to the flow cytometer (MoFlo Astrios EQ cell sorter). Flow cytometric data was analyzed using the FlowJo software package (Treestar). Single, live, CD45-DsRed+ stromal cells (see Extended Data Figure 6a for example of gating strategy) were sorted into individual wells of a 96-well PCR plate filled with 2.6µl of Lysis Buffer (Takara Bio USA, Inc.). Plates were sealed, spun down, snap-frozen and stored at -80°C prior to preparation for cDNA synthesis using the SMARTseq-v4 assay.

Flow cytometry analysis of leukemia burden and DPP4
Leukemic bone marrow was blocked using anti-mouse Fc Block (BD Biosciences, dilution 1 to 50) for 10 minutes at 4°C in Medium 199+ 2% FBS. Surface staining was thereafter performed with CD45-APC/Cy7 (BD Biosciences, dilution 1 to 100) and DPP4-PE (BioLegend, dilution 1 to 20) for 30 minutes at 4°C. The cells were then washed and resuspended in Medium 199+ 2% FBS with 0.25µg 7AAD (BD Biosciences). Flow cytometric analysis was performed on a BD FACS Aria III sorter (BD Biosciences) and all data was analyzed using FlowJo software package (Treestar). See Extended Data Figure 8c for gating strategy used to distinguish DPP4high, DPP4int and DPP4neg cells. Note that DPP4+ cells were defined as DPP4high and DPP4int.

Flow cytometry analysis of leukemia cluster cell surface markers
Leukemic bone marrow was blocked using anti-mouse Fc Block (BD Biosciences, dilution 1 to 50) for 10 minutes at 4°C in Medium 199+ 2% FBS. Surface staining was thereafter performed with Leukemia cluster cocktail (Table S6) for 30 minutes at 4°C. The cells were then washed and resuspended in Medium 199+ 2% FBS with 0.25µg 7AAD (BD Biosciences). Flow cytometric analysis was performed on a BD FACS Aria III sorter (BD Biosciences) and all data was analyzed using FlowJo software package (Treestar).

Flow cytometry analysis of intracellular DPP4 staining
Bone marrow from leukemia bearing mice was incubated with anti-mouse Fc block (BD Biosciences, dilution 1 to 50) for 10 minutes at 4°C followed by surface staining with anti-CD45-APC/Cy7 (BD Biosciences, dilution 1 to 100). Samples were washed and stained with LIVE/DEAD fixable viability dye (ThermoFisher) in accordance with the manufacturer's instructions. The cells were thereafter fixed with Cytofix/Cytoperm (BD Biosciences) for 20 minutes at 4°C. 1x Perm/Wash buffer (BD Biosciences) was then used to wash the cells and the cells were incubated with either anti-DPP4 or an isotype control antibody (Biolegend, dilution 1 to 20 for both) that were both conjugated to Alexa Fluor 647 in house (Abcam) for 30 minutes at room temperature. The cells were then washed one last time in Perm/Wash buffer (BD Biosciences) and resuspended in Medium 199+ 2% FBS for analysis. Flow cytometric analysis was performed on an LSR II instrument (BD Biosciences) and all data was analyzed using the FlowJo software package (Treestar).

Flow cytometric analysis of cell cycle
Bone marrow isolated from leukemic mice was blocked with anti-mouse Fc block (BD Biosciences, dilution 1 to 50) for 10 minutes at 4°C. Surface staining with anti-CD45-APC/Cy7 (dilution 1 to 100) and DPP4-PE (dilution 1 to 20) was performed at 4°C for 30 minutes. Following this, the samples were washed in Medium 199+ 2% FBS and then fixed with Cytofix/Cytoperm (BD Biosciences) for 20 minutes at 4°C. The fixed cells were thereafter washed with 1x Perm/Wash buffer (BD Biosciences) and resuspended in Perm/Wash buffer containing anti-Ki67-AF647 at a 1:10 dilution for a 30-minute incubation. The samples were washed one more time with 1x Perm/Wash buffer (BD Biosciences) and then incubated in 1x Perm/Wash buffer (BD Biosciences) with 2µg/ml DAPI (Biolegend) for 10 minutes. Finally, the samples were spun down to remove the DAPI-containing buffer and resuspended in Medium 199+ 2% FBS for analysis. Flow cytometric analysis was performed on a BD FACS Aria III sorter (BD Biosciences) and all data was analyzed using FlowJo software package (Treestar).

Analysis of DPP4 expression following co-culture
250,000 MLL-AF9 or HoxA9-Meis1 leukemia cells were plated at a 1:1 ratio with the following stromal cell lines: NIH3T3 (ATCC), MS-5 (RIKEN), MLO-A5 (Kerafast), MC-3T3-E1 (ATCC), and SV40 immortalized bone marrow stroma (see separate methods section). The cells were grown in RPMI-1640 (Gibco) supplemented with 1% Penicillin/Streptomycin (Gibco), and 10% FBS (Gibco). MLL-AF9 cultures were supplemented with 20 ng/ml SCF, 10 ng/ml IL-3 and 10 ng/ml IL-6 (all cytokines from Peprotech). HoxA9-Meis1 cell cultures were instead grown in 100 ng/ml SCF and 5 ng/ml IL-3. The cells were co-cultured for 3 days. For flow cytometric analysis of DPP4, the co-cultures were trypsinized and subsequently blocked with murine Fc block (dilution 1 to 50) for 10 minutes at 4°C. Surface staining with anti-CD45-APC/Cy7 (dilution 1 to 100) and DPP4-PE (dilution 1 to 20) was then carried out for 30 minutes at 4°C. The cells were thereafter washed and resuspended in Medium 199 +2% FBS with 0.25µg 7AAD (BD Biosciences) for analysis. Flow cytometric analysis was performed on a LSR II instrument (BD Biosciences) and all data was analyzed using FlowJo software package (Treestar). For the analysis of DPP4 mean fluorescence intensity (MFI), each sample was normalized to a corresponding isotype control antibody-stained sample.

Flow cytometry analysis of myeloid lineage markers
Bone marrow collected from C57Bl/6J mice was stained with HSPC cocktail (Table S6) for 45 minutes at 4°C. The cells were then washed and resuspended in Medium 199+ 2% FBS with 0.25µg 7AAD (BD Biosciences). Flow cytometric analysis was performed on a BD FACS Aria III sorter (BD Biosciences) and all data was analyzed using FlowJo software package (Treestar).

Instrument

BD FACS Aria III, BD LSRII and MoFlo Astrios EQ

Software

FlowJo 10.8.0

Cell population abundance

The identity of single sorted leukemia cells was validated by the subsequent RNAseq analysis.

Gating strategy

For all gating strategies cells are discriminated based on SSC and FSC followed by selection of live singlets. For gating strategies for individual panels please see corresponding figures.

☒ Tick this box to confirm that a figure exemplifying the gating strategy is provided in the Supplementary Information.
